# Investigation of boron–fluorine bond reactivity in *meso*-aryl-substituted BODIPYs towards carborane *O*-nucleophiles

**DOI:** 10.3762/bjoc.22.100

**Published:** 2026-09-09

**Authors:** Andrei V Zaitsev, Alexander F Smol’yakov, Victoria M Alpatova, Elena G Kononova, Anatolii Botezatu, Valentina A Ol’shevskaya

**Affiliations:** 1 A. N. Nesmeyanov Institute of Organoelement Compounds of Russian Academy of Sciences, 28 Bld. 1 Vavilov Street, 119334 Moscow, Russian Federation

**Keywords:** BODIPY, carboranes, fluoride substitution, synthesis

## Abstract

In this work *meso*-aryl-substituted BODIPYs (Ar = Ph, *p*-Br-C_6_H_4_, C_6_F_5_) were used as starting compounds for modification of their boron atoms with carborane *O*-nucleophiles to afford novel boron-substituted carborane BODIPY derivatives. This type of reactions has not been used previously to prepare carborane structures. The reactions proceeded via the cleavage of boron–fluorine bonds in BODIPYs under the action of AlCl_3_ followed by the treatment with carborane *O*-nucleophiles such as 1-hydroxymethyl-*o*-carborane or 1,2-dihydroxymethyl-*o*-carborane. While reactions of *meso*-aryl-substituted BODIPYs with 1-hydroxymethyl-*o*-carborane proceeded selectively, forming mono- or dicarborane-substituted BODIPYs, the reactions of 1,2-bis(hydroxymethyl)-*o*-carborane were not selective yielding a mixture of products. The structures of novel compounds were confirmed by ^1^H and ^11^B NMR and mass spectrometry. The structures of some prepared carborane BODIPY compounds were further supported by their single X-ray crystal structures.

## Introduction

BODIPYs, 4,4-difluoro-4-bora-3a,4a-diaza-*s*-indacenes, are important compounds that have been intensively studied since their discovery by A. Treibs and F. H. Kreuzer in 1968 [1]. They have a number of valuable physicochemical properties, such as excellent chemical and photostability, high molar absorption coefficients, high fluorescence quantum yields, relatively small Stokes shifts, narrow absorption and emission peaks [2–3] which enable their use in a variety of applications [4–8]. Based on these excellent photophysical properties functionalized BODIPY systems have found applications in various fields, such as cell imaging [9], fluorescent probes [10], luminescent devices [11], organic light-emitting diodes (OLEDs), laser dyes [12], molecular switches [13], and non-linear optical materials [14]. These compounds are also actively used in biomedical applications as triplet photosensitizers [15], agents for photodynamic (PDT) and photothermal (PTT) antitumor therapies [16–17], agents for bioimaging and disease diagnostics [18] as well as sensors for monitoring a variety of biological processes [19]. An important factor determining the potential of using outstanding BODIPY characteristics is their chemical diversity since several strategies have been developed to introduce a variety of functional groups at different sites on the BODIPY core. The starting BODIPYs are readily prepared by reacting dipyrromethenes with boron trifluoride etherate [20] with the required dipyrromethenes accessible through condensation of either aldehydes with pyrroles and oxidation of the dipyrromethane intermediates or directly through condensation of acyl chlorides or anhydrides with pyrroles [20]. It has been shown that the introduction of substituents has a significant impact on the electronic and photophysical properties of BODIPY and methods have been developed to functionalize every position of the BODIPY core [21]. There are three main specific sites in the BODIPY skeleton: the *meso*-position (C8 carbon atom), the carbon atoms of the pyrrole fragments and the boron site whose modifications enable subtle tuning of the compounds’ physicochemical and biological properties, solubility, and reactivity affording a variety of derivatives with multiple advantages [22]. To increase the application potential of BODIPYs the modification of the *meso*-position could be important. *Meso*-substituents at the C8 site of BODIPY influence the photophysical, electrochemical, and structural properties exhibiting pronounced effects on fluorescence wavelengths, quantum yields, and Stokes shifts as well as biomedical applications [23]. Due to the wide variety of functional groups that can be attached to the C8 atom the introduction of a particular functionality at this position mainly depends on the intended area of application. Moreover, the free rotation of the *meso*-substituents in the BODIPY acts as a primary control for its optical and photophysical properties. Pyrrole units also play an important role in BODIPY structures defining their unique photophysical properties, such as optical stability, intense fluorescence, and high extinction coefficients as well as versatile sites for chemical modification [24]. In addition to functionalization of the BODIPY core at the *meso*- and pyrrole carbon sites, the substitution of fluorine atoms in the BF_2_ unit was realized to give a wide range of new derivatives. The boron center of BODIPY provides free rotation of substituents as well as the ability to construct asymmetric dyes by introducing nucleophilic substituents at the boron atom through replacement of the boron-bound fluorine which can lead to an increase in fluorescence quantum yields and Stokes’ shift of the BODIPY fluorophore, improving chemical stability, and increasing water solubility [25]. There are reasons to assume that the same structure fragment can exert different properties in BODIPY according to its site of attachment [24,26]. Depending on the direction of BODIPYs applications, a search for new molecular structures is underway. Among the various BODIPY derivatives that have been studied over the years, conjugates with carborane polyhedra have demonstrated promising efficacy in improving the photophysical properties with influences on other conjugate characteristics such as cellular uptake in PDT [27] or in optoelectronic applications, including organic light-emitting diodes (OLEDs) and sensors [28]. The conjugation of icosahedral carborane clusters to the BODIPY core has been of great interest because of their special features such as remarkable chemical and thermal stability along with high hydrophobicity and lipophilicity, outstanding heat-oxidation and metabolic stability. Carboranes can be regarded as three-dimensional quasi-aromatic systems with a high level of delocalization of their skeleton electrons on a cage framework [29]. This unique physical property determines various functions of hybrid systems containing carborane clusters as substituents and carborane hybrid structures have found a broad range of applications in materials science, medicine, organic synthesis, and other areas [30–32]. Over the years, carborane compounds have been intensively studied as fluorophores [33], enzyme inhibitors [34], and potential agents for boron neutron capture therapy (BNCT), a binary method for cancer treatment that employs the thermal neutron capture reaction of ^10^B isotope to kill tumor cells by means of an intracellular fission process [35]. We have been interested in the synthesis of carborane BODIPY derivatives for PDT applications by functionalization of the *meso* or pyrrole sites of BODIPY with carborane polyhedral compounds [36–38]. In continuation of our work in this area, the functionalization of the BF_2_ fragment in BODIPY with carborane clusters via the substitution of fluorine with the corresponding carborane *O*-nucleophiles was studied. Such reactions were not described in the literature.

## Results and Discussion

### Synthesis

**Scheme 1:**
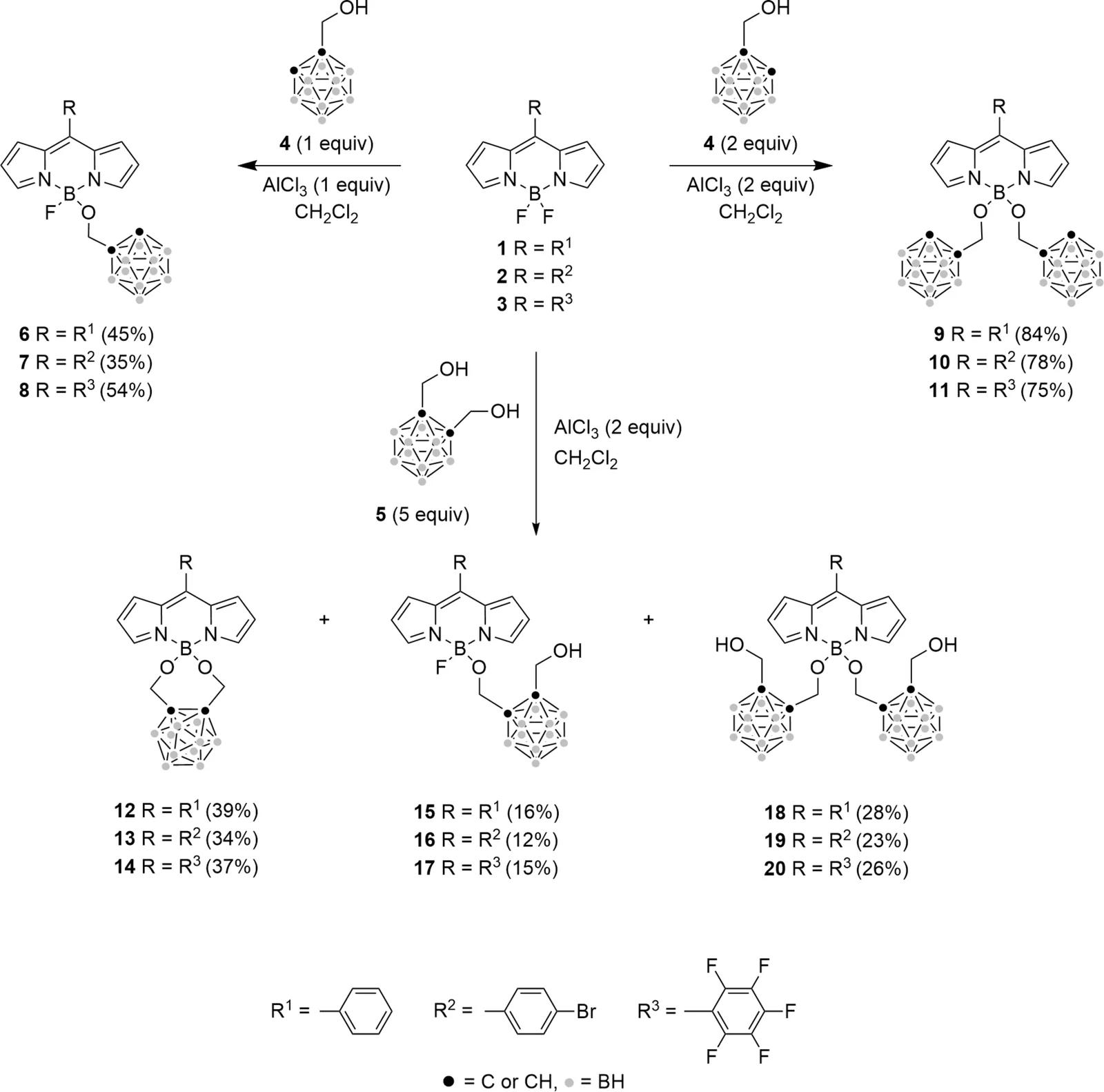
Synthesis of carborane-substituted BODIPY derivatives **6**–**20**.

To study the introduction of carborane *O*-nucleophiles to the BODIPY boron center available pyrrole-unsubstituted 8-aryl-substituted BODIPYs such as 8-phenyl-4,4-difluoro-4-bora-3a,4a-diaza-*s*-indacene (**1**), 8-*p*-bromophenyl-4,4-difluoro-4-bora-3a,4a-diaza-*s*-indacene (**2**), and 8-pentafluorophenyl-4,4-difluoro-4-bora-3a,4a-diaza-*s*-indacene (**3**) prepared according to reported procedures [39–41] were initially used as the starting compounds for the envisaged functionalization with 1-hydroxymethyl-*o*-carborane (**4**) or 1,2-bis(hydroxymethyl)-*o*-carborane (**5**). The reactions of BODIPYs **1**–**3** with carborane **4** (molar ratio 1:1) were conducted in CH_2_Cl_2_ under argon at room temperature via the activation of the BODIPY B–F bond with AlCl_3_ as Lewis acid catalyst [42–43] within 15–20 min followed by the treatment of the reaction mass with carborane **4** to form dark orange 4-carborane-substituted BODIPY derivatives **6**–**8** in 35–54% yields (Scheme 1). Using the same synthetic method, the reactions of BODIPYs **1**–**3** also successfully proceeded with carborane **4** (molar ratio 1:2) (Scheme 1) in CH_2_Cl_2_ to produce the 4,4-dicarborane-substituted BODIPY derivatives **9**–**11** in 75–84% yields (Scheme 1).

Under the conditions developed for the preparation of carborane-substituted BODIPYs from carborane **4**, the reaction of carborane **5** with BODIPYs **1**–**3** in CH_2_Cl_2_ at ambient temperature proceeded less selectively and led to mixtures of boronated BODIPYs despite the increased amount of carborane **5** used in the reactions (Scheme 1).

**Scheme 2:**
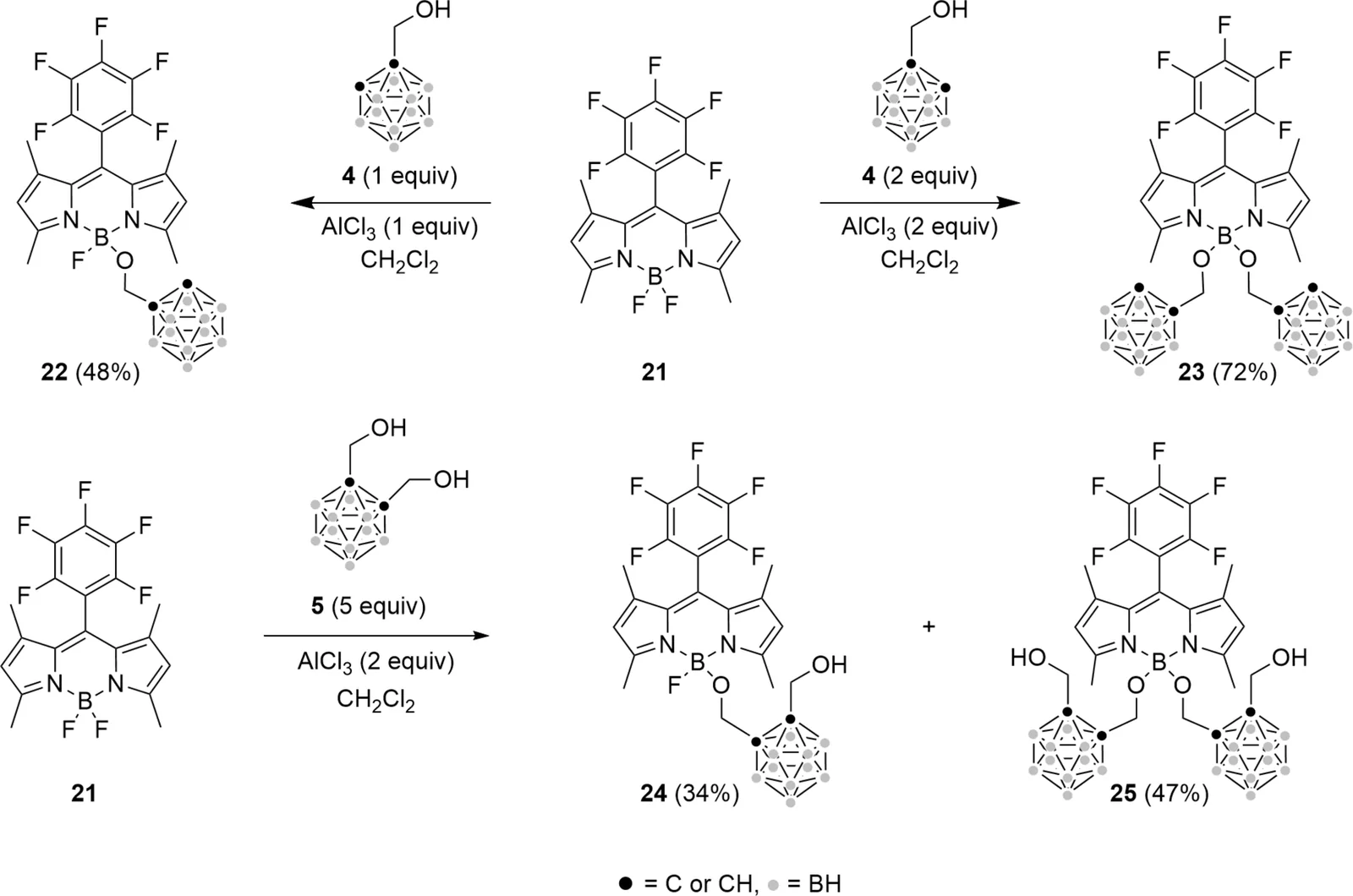
Synthesis of carborane-substituted BODIPY derivatives **22**–**25**.

As a result, BODIPYs **12**–**14** formed via the intramolecular cyclization of diol **5** with the boron center of BODIPY were obtained in yields of 34–39% along with monocarborane-substituted BODIPYs **15**–**17** in 12–16% yield and bis(carboranyl)-substituted BODIPYs **18**–**20** in 23–28% yield, respectively. For the isolation of pure compounds column chromatography on silica gel was performed and all compounds were obtained and characterized (Supporting Information File 1). We also studied the reactivity of the pyrrole-substituted BODIPY 8-pentafluorophenyl-1,3,5,7-tetramethyl-4,4-difluoro-4-bora-3a,4a-diaza-*s*-indacene (**21**) [44] towards carboranes **4** and **5**. The reaction between BODIPY **21** and carborane **4** (at a molar ratio of 1:1 or 1:2) proceeded cleanly in CH_2_Cl_2_ at ambient temperature after the activation of the B–F bond with AlCl_3_ and was completed in 10 min to give the 4-monosubstituted derivative **22** in 48% yield while the disubstituted compound **23** was obtained with a yield of 72% (Scheme 2).

When performing the substitution reaction of the fluoride in BODIPY **21** with carborane **5** using the same reaction conditions a mixture of monosubstituted carborane BODIPY **24** (34% yield) and dicarborane-substituted BODIPY **25** (47% yield) was obtained (Scheme 2). It should be noted that in the case of pyrrole-substituted BODIPY **21** at any ratio of AlCl_3_ (from 1 to 2 equivalents) and carborane **5** (from 1 to 5 equivalents), no formation of the cyclized product through reaction of both hydroxy groups of the carborane with the boron atom in BODIPY was observed. The reason for the difference in reactivity when using BODIPY **21** is apparently related to steric hindrance by methyl groups in the BODIPY 3,5-positions.

### Spectroscopic data

All synthesized compounds were isolated by column chromatography and their structures were fully characterized by UV–vis, IR and ^1^H, ^11^B, ^11^B{^1^H} NMR spectroscopies and ESI mass spectrometry. The IR spectra of compounds **6**–**11** and **12**–**20** showed stretching bands in the region 2573–2590 cm^−1^ and 1378–1390 cm^−1^ attributed to BH and B–O groups, respectively. The IR spectra of BODIPYs **15**–**20**, **24**, **25** containing an unsubstituted carborane hydroxy group display a characteristic OH absorption band at 3362–3446 cm^−1^. Absorption bands at 1556–1580 cm^−1^ correspond to C=C and C=N stretching vibrations and bands in the range of 1158–1186 cm^−1^ are attributable to B–F stretching vibrations (compounds **6**–**8**, **15**–**17**, **22**, **24**). The ^1^H NMR spectra further supported the structure of the synthesized compounds. The hydroxy protons of compounds **15**–**20** as well as for compounds **24** and **25** appear as broad singlets at δ = 4.10–4.42 ppm. The signals of the carborane CH protons in compounds **6**–**11** and **22** were observed at δ = 4.02–4.25 ppm as broad signals. The ^1^H NMR spectra show the signals of the CH_2_OB protons at 3.39–4.38 ppm as singlets for compounds **6**–**14**. The expected signals with appropriate multiplicities were observed for pyrrole CH protons in the range of δ = 6.59–8.01 ppm and manifested themselves as singlets, doublets or as doublet of doublets (see Supporting Information File 1 for details). The protons of pyrrole methyl groups in BODIPYs **22**–**25** appeared as singlets in the region of δ = 2.46–2.60 ppm. The ^11^B{^1^H} NMR spectra also confirmed the formation of the expected compounds, showing broad resonances in the region from δ = 0.5 to −13.9 ppm for carborane BODIPY derivatives **6**–**20** and **22**–**25**. The ^11^B NMR spectra of the prepared BODIPY derivatives display doublets or broad singlets between δ = 0.5–1.8 ppm.

**Figure 1:**
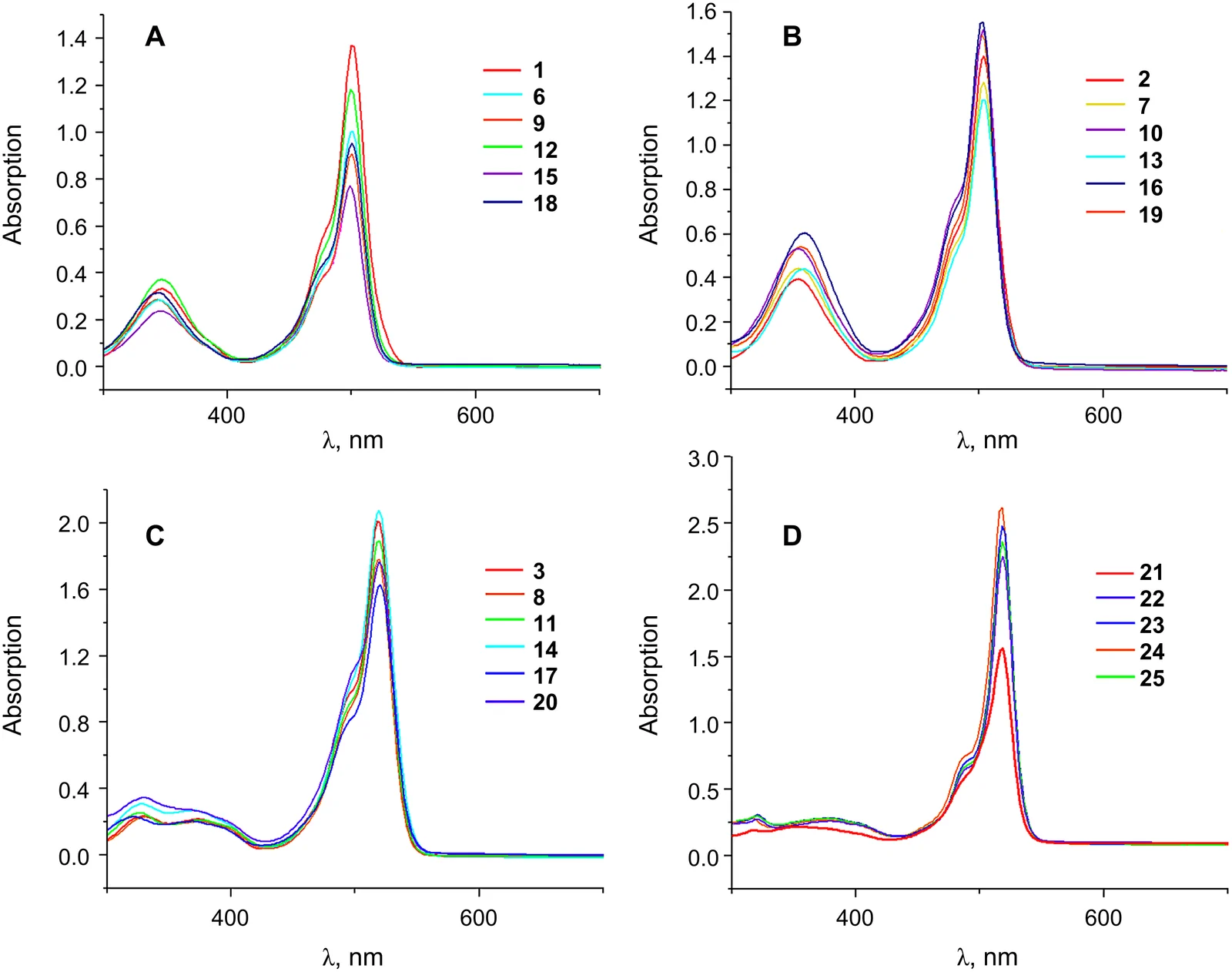
Absorption spectra of compounds **1**–**3**, **6**–**25** in CH_2_Cl_2_.

The UV–vis spectra in CH_2_Cl_2_ of all the prepared compounds are characterized by the presence of two absorption bands (Figure 1). However, the bands of compounds containing a pentafluorophenyl substituent (**8**, **11**, **14**, **17**, **20**, **22**–**25**) are red-shifted (460–480, 518–520 nm) compared to those with phenyl or *p*-bromophenyl fragments (**6**, **7**, **9**, **10**, **12**, **13**, **15**, **16**, **18**, **19**). The absorption bands of the latter compounds are observed in the ranges of 340–360 and 500–505 nm. This phenomenon reflects the influence of fluorine atoms on the HOMO–LUMO energy gap. Modifications of the starting BODIPYs **1**, **2**, **3** and **21** with carboranes **4** or **5** did not lead to a significant change in the position of the absorption bands but in the case of products **22**–**25** an increase of the extinction coefficient ε value by one and a half times was observed.

**Figure 2:**
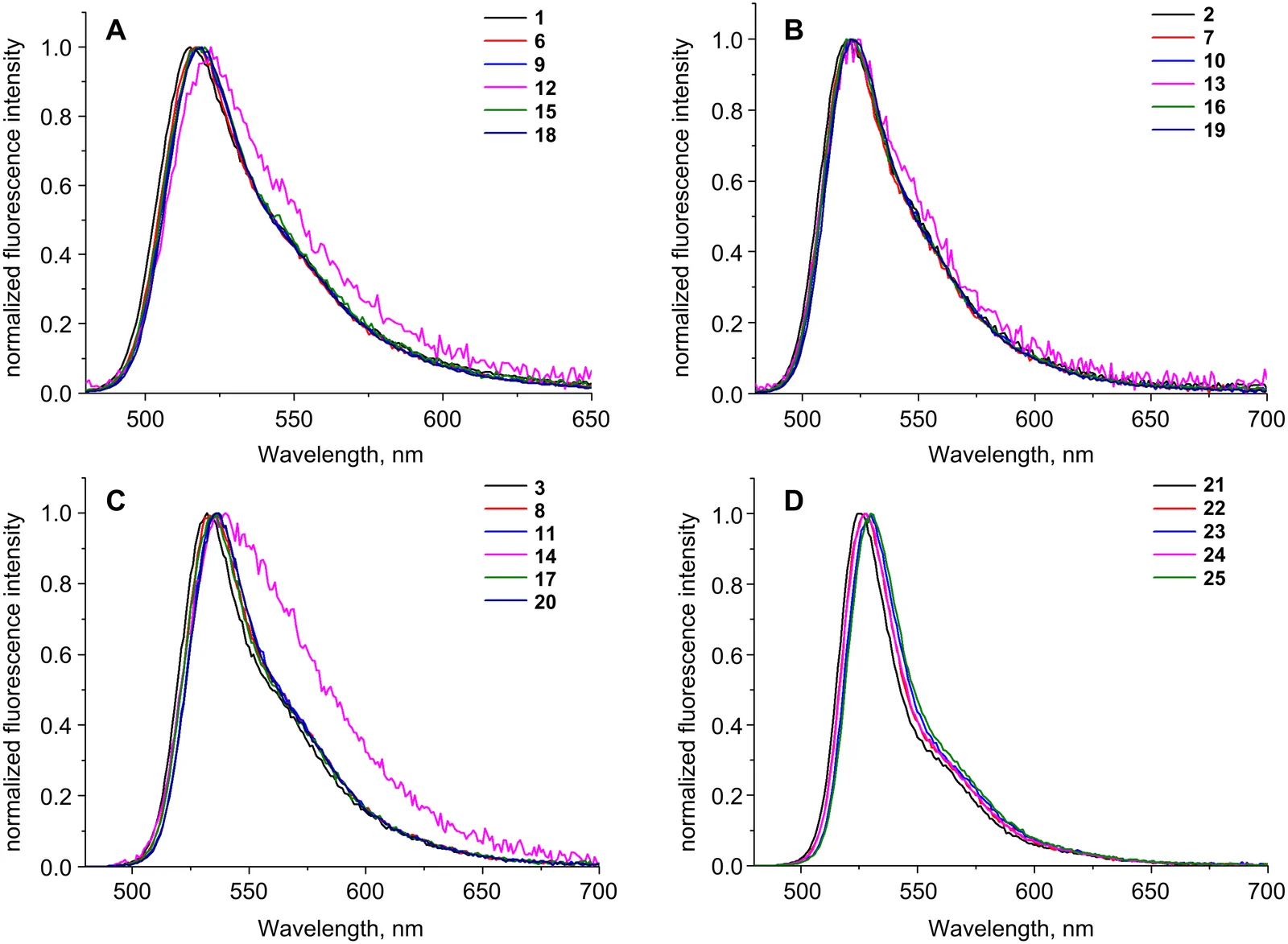
Normalized fluorescence spectra of compounds **1**–**3**, **6**–**25** in acetonitrile.

To determine the fluorescence quantum yields (Φ_fl_) and Stokes shifts, additional UV–vis (Figure S67, Supporting Information File 1) and fluorescence (Figure 2) spectra were recorded in acetonitrile at ambient temperature for all compounds. The data are summarized in Table 1. The consistently higher fluorescence quantum yields observed for BODIPY derivatives with the 8-pentafluorophenyl group (**8**, **11**, **14**, **17**, **20**, **22**–**25**) compared to their phenyl and *p*-bromophenyl analogs (**6**, **7**, **9**, **10**, **12**, **13**, **15**, **16**, **18**, **19**) can be explained by the strong electron-withdrawing character of the pentafluorophenyl group. This suppresses the photoinduced electron transfer from the aryl ring in the *meso*-position to the excited BODIPY core. Additionally, the steric bulk of the *ortho-*fluorine substituents in compounds **8**, **11**, **14**, **17**, **20**, **22**–**25** restricts rotation of the *meso*-aryl ring, minimizing non-radiative vibrational relaxation and further increasing the emission efficiency [45]. Notably, the unsubstituted phenyl derivatives **6**, **9**, **12**, **15**, **18** showed intermediate fluorescence quantum yields between the values for the *p*-bromophenyl analogs **7**, **10**, **13**, **16**, **19** and pentafluorophenyl analogs **8**, **11**, **14**, **17**, **20**, **22**–**25**. The lower fluorescence quantum yield of the former relative to the phenyl analogs can be explained by the effect of the heavy bromine atom, which strengthens the spin–orbit coupling and promotes the intersystem crossing to the triplet manifold.

**Table 1:** Optical properties of studied compounds in acetonitrile.

Compound	λ_max_^abs^ (nm)	λ_max_^fl^ (nm)	Φ_fl_	Stokes shift (cm^−1^)

**1**	496	515	0.17	744
**2**	499	521	0.05	847
**3**	512	532	0.92	734
**6**	498	517	0.13	738
**7**	500	522	0.04	843
**8**	515	536	0.72	761
**9**	498	518	0.14	775
**10**	503	521	0.03	687
**11**	515	537	0.88	796
**12**	496	522	0.02	1004
**13**	495	524	0.01	1118
**14**	510	540	0.24	1089
**15**	495	520	0.03	971
**16**	501	519	0.11	692
**17**	515	535	0.70	726
**18**	500	519	0.16	732
**19**	500	521	0.09	806
**20**	516	537	0.84	758
**21**	513	526	0.98	482
**22**	514	528	0.92	516
**23**	516	530	0.77	512
**24**	514	528	0.91	516
**25**	516	530	0.98	512

The Stokes shifts ranged from 482 to 1118 cm^−1^, falling within the typical range reported for BODIPY derivatives [46]. The smallest Stokes shifts were observed for the 8-pentafluorophenyl derivatives **8**, **11**, **14**, **17**, **20**, **22**–**25**, which is consistent with the restricted rotation of the *meso*-aryl ring discussed above. The largest Stokes shifts were found for compounds **12**–**14**, which contain the 1,2-di(oxymethyl)-*o*-carborane moiety. This observation aligns with the low fluorescence quantum yields of these derivatives, as the substantial Stokes shifts indicate extensive excited-state reorganization and enhanced non-radiative decay. The rigid cyclic linkage in compounds **12**–**14** creates steric strain that distorts the BODIPY core, promoting energy dissipation through non-radiative channels [47].

**Figure 3:**
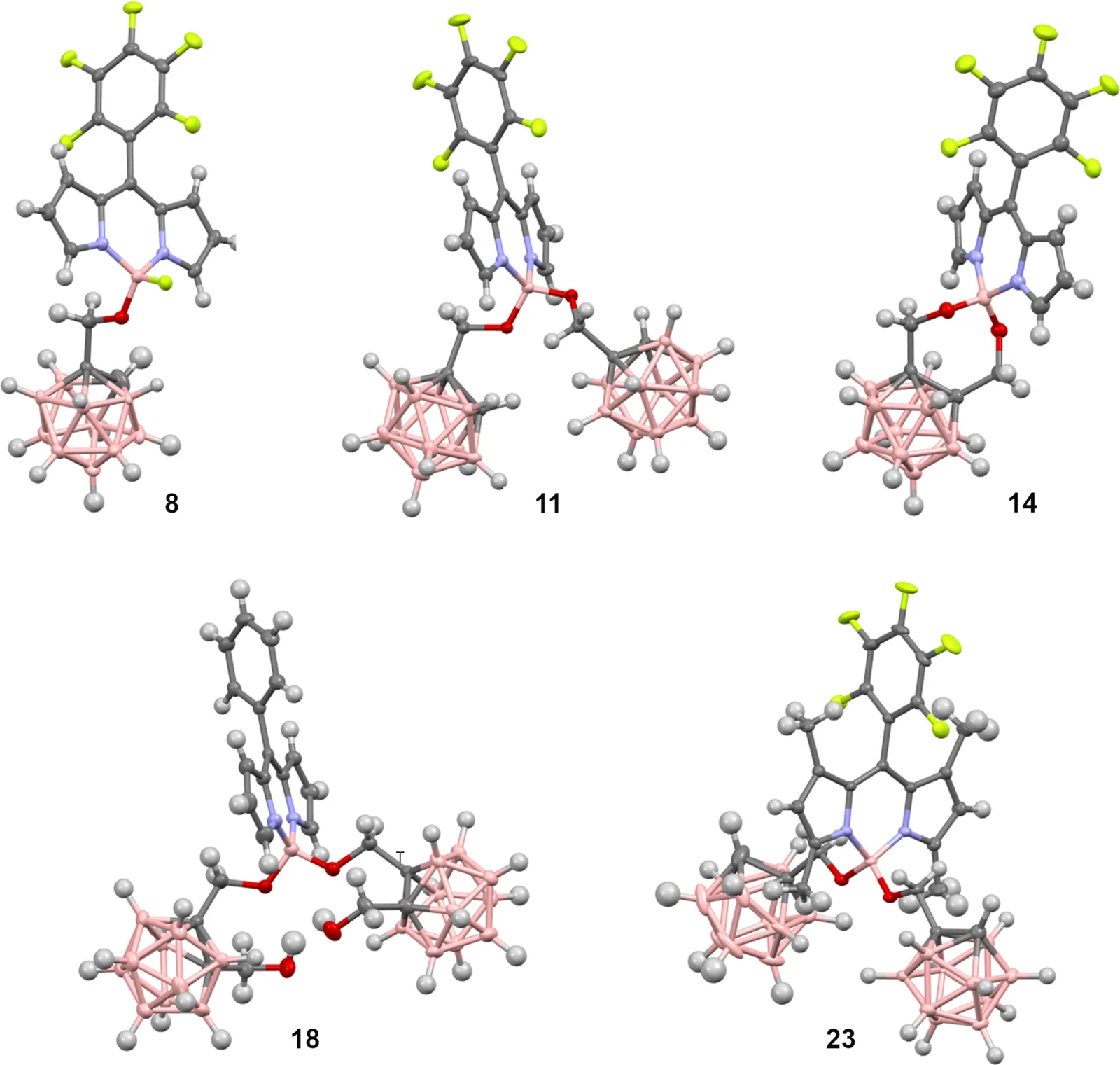
Molecular structure of compounds **8**, **11**, **14**, **18** and **23**.

The molecular structure of compounds **8**, **11**, **14**, **18** and **23** was determined by means of single crystal X-ray diffraction studies (Figure 3). Crystal data and structure refinement parameters of compounds **8**, **11**, **14, 18** and **23** are presented in Table S1 (Supporting Information File 1).

## Conclusion

In summary, carborane BODIPY-based compounds have attracted attention due to their promising potential of applications ranging from materials to biology and medicine. In this work a series of carborane-substituted BODIPYs was synthesized for the first time by the substitution of the fluoride atoms in BODIPYs with carborane *O*-nucleophiles. The synthetic method includes the cleavage of boron–fluorine bonds in BODIPYs under the action of AlCl_3_ and treatment of the reaction mass with 1-hydroxymethyl- or 1,2-dihydroxymethyl-*o*-carboranes in CH_2_Cl_2_. The structure of all prepared compounds was fully characterized. It has been revealed that reaction selectivity depends on the carborane structure and is non-selective in the case of 1,2-dihydroxymethyl-*o*-carborane. The results described in this work demonstrated the way for modification of BODIPY core with carborane polyhedra via the employment of fluoride atoms substitution.

## Supporting Information

File 1 Experimental procedures, characterization and plots of NMR and mass spectra for synthesized compounds **6**–**20** and **22**–**25** and crystal data for compounds **8**, **11**, **14**, **18** and **23**.

File 2 Crystallographic information files (CIF) of compounds **8**, **11**, **14**, **18** and **23**.

## Data Availability

All data that supports the findings of this study is available in the published article and/or the supporting information of this article.The crystal data have been deposited in the Cambridge Crystallographic Data Centre (https://www.ccdc.cam.ac.uk/) deposition numbers CCDC 2547857–2547861.
