# Supplementary material for: Investigation of boron–fluorine bond reactivity in meso-aryl-substituted BODIPYs towards carborane O-nucleophiles
Source: Beilstein J Org Chem. 2026 Sep 9;22:1237–45. doi: 10.3762/bjoc.22.100 (PMC13575341; doi:10.3762/bjoc.22.100)
Supplement: Supplementary file 1 [file Beilstein_J_Org_Chem-22-1237-s001.pdf]

BEILSTEIN JOURNAL OF ORGANIC CHEMISTRY

Supporting Information

for

Investigation of boron–fluorine bond reactivity in *meso*-aryl-substituted BODIPYs towards carborane *O*-nucleophiles

Andrei V. Zaitsev, Alexander F. Smol'yakov, Victoria M. Alpatova, Elena G. Kononova, Anatolii Botezatu and Valentina A. Ol'shevskaya

Beilstein J. Org. Chem. **2026,** *22,* 1237–1245. doi:10.3762/bjoc.22.100

Experimental procedures, characterization and plots of NMR and mass spectra for synthesized compounds 6–20 and 22–25 and crystal data for compounds 8, 11, 14, 18 and 23

License and Terms: This is a supporting information file under the terms of the Creative Commons Attribution License (https://creativecommons.org/licenses/by/4.0). Please note that the reuse, redistribution and reproduction in particular requires that the author(s) and source are credited and that individual graphics may be subject to special legal provisions.
The license is subject to the *Beilstein Journal of Organic Chemistry* terms and conditions: (https://www.beilstein-journals.org/bjoc/terms)

Contents

Experimental procedures, characterization and copies of NMR and mass spectra for synthesized compounds **6**–**20** and **22**–**25** and crystal data for compounds **8**, **11**, **14, 18** and **23**

Experimental

General remarks

All reactions were performed under an argon atmosphere. All solvents were distilled from the proper drying agents previous to use. All reagents were from Sigma-Aldrich unless specified otherwise. ^{1}H and ^{11}B NMR spectra were recorded on a Bruker Avance-400 spectrometer operating at 400.13 MHz for ^{1}H NMR and 128.38 MHz for ^{11}B NMR. ^{19}F NMR spectra were recorded on Bruker Avance-300 spectrometer operating at 282.40 MHz for ^{19}F NMR. Chemical shifts (δ) were referenced to the residual solvent peak ($CDCl_3$, ^{1}H: 7.26 ppm for ^{1}H, external $BF_3·OEt_2$ for ^{11}B and external $CFCl_3$ for ^{19}F). IR spectra were recorded on a Bruker FTIR spectrometer Tensor 37 in KBr pellets. The UV–vis spectra were measured on a computerized spectrophotometer Carl Zeiss Specord M 40 in CH_2Cl_2. Electrospray ionization mass spectra (ESIMS) were registered on Shimadzu LCMS-2020 High performance liquid chromatograph mass spectrometer with electrospray ionization (ESI) method and single quadrupole detector (negative and positive ion mode). Desolvation line/heat block temperatures were 250/400°C. Nitrogen (99.5%) was used as nebulizer and drying gas. Acetonitrile (99.9+% HPLC gradient grade, Chem-Lab) was used as mobile phase with flow rate 0.4 mL/min without any pretreatment. Compounds were dissolved in CH_3CN, injection volume 0.1–1 µL. The mass range between 200 and 2000 was scanned. The course of reactions and purity of compounds were monitored by TLC using on Silufol plates (elution with hexane/CH_2Cl_2 2:1, 1:1, 1:2 and CH_2Cl_2/acetone 95:5). Macherey-Nagel silica gel 60, particle size 0.040–0.063 mm (230–400 mesh) was used for column chromatography (elution with hexane/CH_2Cl_2 1:2 for compounds **6–8**, **12**, **15**, **18**, **22**; hexane/CH_2Cl_2 1:1, for compounds **9–11**, **23**; CH_2Cl_2 for compounds **13**, **16**, **19**, **24**; CH_2Cl_2/acetone 20:1 for compounds **14**, **17**, **20, 25**).

Fluorescence spectroscopy

For the fluorescence spectra a Cary Eclipse spectrofluorometer has been used. All measured luminescence spectra were corrected for nonuniformity of detector spectral sensitivity. The luminescence quantum yields were calculated using the following equation:

$$\varphi_i = \varphi_0 \frac{(1 - 10^{-A_0}) * S_i * {n_i}^2}{(1 - 10^{-A_i}) * S_0 * {n_0}^2},$$

where φ_i and φ_0 are the luminescence quantum yields of the studied solution and the standard compound, respectively; A_i and A_0 are the absorptions of the studied solution and the standard (rhodamine 6G, φ_{fl} = 0.95 in ethanol), respectively; S_i and S_0 are the areas underneath the curves of the luminescence spectra of the studied solution and the standard, respectively; and n_i and n_0 are the refractive indices of the solvents for the substance under study and the standard compound (n_i = 1.3441, acetonitrile; n_0 = 1.361, ethanol).

X-ray crystallography

Single-crystal X-ray diffraction experiments were carried out with a Bruker SMART APEX II diffractometer (graphite monochromated Mo K_α radiation, λ = 0.71073 Å, ω-scan technique). The APEX II software [1] was used for collecting frames of data, indexing reflections, determination of lattice constants, integration of intensities of reflections, scaling and absorption correction. All calculations (space group and structure determination, refinements, graphics, and structure reporting) were made using the SHELXL2014 [2] and OLEX2 [3] program packages. Experimental details and crystal parameters are listed in Table 1. The structures were solved by direct methods and refined by the full-matrix least-squares technique against F^2 with anisotropic thermal parameters for all non-hydrogen atoms. The hydrogen atoms were placed geometrically and included in the structure factors calculations in the riding motion approximation. The crystallographic data (excluding the structural factors) for the structure of compounds **8**, **11**, **14, 18** and **23** have been deposited at the Cambridge Crystallographic Data Centre (CCDC) as supplementary publications No. 2547860 No. 2547861 No. 2547859 No. 2547858 and 2547857, respectively.

Synthesis and analysis of compounds

General procedure for the preparation of carboranyl-BODIPYs 6–8, 22. To a solution of BODIPY **1**–**3** or **21** (0.3 mmol) in dry CH_2Cl_2 (50 mL) $AlCl_3$ (0.3 mmol) was added and the reaction mixture was stirred under argon for 20 min at room temperature in the dark. Then carborane **4** (0.3 mmol) was added and the reaction mixture was stirred under argon for 10 min at room temperature. Water (50 mL) was added and the organic phase was washed with water (2 × 50 mL) and dried over Na_2SO_4. After removal of the solvent in vacuo, the residue was purified by column chromatography on silica gel using hexane/CH_2Cl_2 1:2 as an eluent.

Compound 6. Yield 57 mg (45%) as dark yellow crystals. UV–vis (CH_2Cl_2) λ_{max} nm ($\varepsilon \cdot 10^{-3}$): 232 (22.3), 346 (16.9), 502 (58.0). IR spectra (KBr), ν, cm^{-1}: 3092 (carborane CH), 2590 (carborane BH), 1559 (C=C, C=N), 1389 (B–O), 1158 (B–F). ^1H NMR (400.13 MHz, $CDCl_3$) δ, ppm: 7.82 (s, 2H, pyrrole), 7.65–7.54 (m, 5H, Ph), 6.98 (d, *J*=4.1 Hz, 2H, pyrrole), 6.60 (dd, *J*=4.1, 1.6 Hz, 2H, pyrrole), 4.25 (br. s., 1H, carborane CH), 3.69 (s, 2H, CH_2). ^{11}B NMR (128.38 MHz, $CDCl_3$) δ, ppm: 0.5 (d, *J* = 26 Hz, 1B, O–*B*–F), −3.0 to −6.0 (m, 2B), −8.6 to −13.7 (m, 8B). MS (ESI): *m/z* $[M–H]^-$ calcd. for $C_{18}H_{24}B_{11}FN_2O$ 421.3; found 421.3.

Compound 7. Yield 52 mg (35%) as dark orange crystals. UV–vis (CH_2Cl_2) λ_{max} nm ($\varepsilon \cdot 10^{-3}$): 233 (40.9), 354 (25.3), 504 (69.7). IR spectra (KBr), ν, cm^{-1}: 3062 (carborane CH), 2584 (carborane BH), 1567 (C=C, C=N), 1378 (B–O), 1160 (B–F). ^1H NMR (400.13 MHz, $CDCl_3$) δ, ppm: 7.82 (s, 2H, pyrrole), 7.71 (d, *J* = 8.6 Hz, 2H, Ph), 7.46 (d, *J* = 8.0 Hz, 2H, Ph), 6.95 (d, *J* = 4.1 Hz, 2H, pyrrole), 6.61 (dd, *J* = 4.3, 1.8 Hz, 2H, pyrrole), 4.22 (br. s., 1H, carborane CH), 3.68 (s, 2H, CH_2). ^{11}B NMR (128.38 MHz, $CDCl_3$) δ, ppm: 0.5 (d, *J* = 26.0 Hz, 1B, F–*B*–O), −3.1 to −5.9 (m, 2B), −8.6 to −13.9 (m, 8B). MS (ESI): *m/z* $[M–H]^-$ calcd. for $C_{18}H_{23}B_{11}BrFN_2O$ 500.2; found 500.3

Compound 8. Yield 83 mg (54%) as dark reddish-green crystals. UV–vis (CH_2Cl_2) λ_{max} nm ($\varepsilon \cdot 10^{-3}$): 226 (20.3), 330 (7.2), 376 (6.4), 518 (53.9). IR spectra (KBr), ν, cm^{-1}: 3048 (carborane CH), 2590 (carborane BH), 1563 (C=C, C=N), 1388 (B–O), 1162 (B–F). ^1H NMR (400.13 MHz, $CDCl_3$) δ, ppm: 7.87 (s, 2H, pyrrole), 6.85 (dd, *J*=4.0, 0.8 Hz, 2H, pyrrole), 6.62 (dd, *J*=4.3, 1.1 Hz, 2H, pyrrole), 4.15 (br.s., 1H, carborane CH). 3.69 (s, 2H, CH_2). ^{11}B NMR (128.38 MHz, $CDCl_3$) δ, ppm: 0.5 (d, *J*=26 Hz, 1B, O–*B*–*F*), –2.9 to –6.2 (m, 2B), –8.6 to –13.9 (m, 8B). ^{19}F NMR (282.40 MHz, $CDCl_3$); δ, ppm: –136.7 (d, *J*=22 Hz, 1F, Ar-F-*ortho*), –136.0 (d, *J*=22 Hz, 1F, Ar-F-*ortho*), –148.7 (t, *J* = 22.0 Hz, 1F, Ar-F-*para*), –150.5 to –150.7 (m, 1F, B–F), –158.8 to –159.0 (m, 2F, Ar-F-*meta*). MS (ESI): *m/z* $[M–H]^-$ calcd. for $C_{18}H_{19}B_{11}F_6N_2O$ 512.2; found 512.2.

Compound 22. Yield 81 mg (48%) as reddish-green crystals. UV–vis (CH_2Cl_2) λ_{max} nm ($\varepsilon \cdot 10^{-3}$): 228 (40.9), 316 (11.9), 378 (11.8), 518 (131.2). IR spectra (KBr), ν, cm^{-1}: 3082 (carborane CH), 2585 (carborane BH), 1556 (C=C, C=N), 1377 (B–O), 1185 (B–F). ^1H NMR (400.13 MHz, $CDCl_3$) δ, ppm: 6.10 (s, 2H, pyrrole), 4.19 (br.s., 1H, carborane CH), 3.35 (s, 2H, CH_2), 2.52 (s, 6H, CH_3), 1.64 (s, 6H, CH_3). ^{11}B NMR (128.38 MHz, $CDCl_3$) δ, ppm: 0.9 (d, *J* = 21 Hz, 1B, F–*B*–*O*), –3.2 - –13.6 (m, 10B). ^{19}F NMR (282.40 MHz, $CDCl_3$); δ, ppm: –139.4 (d, *J* = 17 Hz, 1F, Ar-F-*ortho*), –139.9 (d, *J* = 18 Hz, 1F, Ar-F-*ortho*), –149.9 (t, *J* = 21 Hz, 1F, Ar-F-*para*), –152.9 to –153.2 (m, 1F, B–F), –159.0 (td, *J* = 22, 8 Hz, 1F, Ar-F- *meta*), –159.2 (td, *J* = 22,8 Hz, 1F, Ar-F-*meta*). MS (ESI): *m/z* $[M–H]^-$ calcd. for $C_{22}H_{27}B_{11}F_6N_2O$ 567.3; found 567.2.

General procedure for the preparation of carboranyl-BODIPYs 9–11, 23 To a solution of BODIPY **1**–**3** or **21** (0.3 mmol) in dry CH_2Cl_2 (50 mL) $AlCl_3$ (0.6 mmol) was added and reaction mixture was stirred under argon for 20 min at ambient temperature in the dark. Then carborane **4** (0.7 mmol) was added and reaction mixture was stirred under argon for 10 min in the dark. Water (50 mL) was added, and organic phase was washed with water (2x50 mL)

and dried over Na_2SO_4. After removal of the solvent *in vacuo*, the residue was purified by column chromatography on silica gel using hexane–CH_2Cl_2 system (1:1) as an eluent.

Compound 9. Yield 145 mg (84%) as yellow crystals. UV–vis (CH_2Cl_2) λ_{max} nm ($\varepsilon \cdot 10^{-3}$): 228 (17.8), 348 (23.8), 500 (52.3). IR spectra (KBr), ν, cm^{-1}: 3079 (carborane CH), 2588 (carborane BH), 1578 (C=C, C=N), 1388 (B–O). ^{1}H NMR (400.13 MHz, $CDCl_3$) δ, ppm: 7.68 (s, 2H, pyrrole), 7.62-7.56 (m, 5H, Ph), 7.02 (d, *J*=3.8 Hz, 2H, pyrrole), 6.64 (dd, *J*=4.1, 1.3 Hz, 2H, pyrrole), 4.09 (br. s., 2H, carborane CH), 3.63 (s, 4H, CH_2). ^{11}B NMR (128.38 MHz, $CDCl_3$) δ, ppm: 0.6 (br.s., 1B, O–*B*–*O*), –2.8 to –5.6 (m, 4B), –8.7 to –12.4 (m, 16B). MS (ESI): *m/z* $[M–H]^-$ calcd. for $C_{21}H_{37}B_{21}N_2O_2$ 575.5; found 575.5.

Compound 10. Yield 153 mg (78%) as orange crystals. UV–vis (CH_2Cl_2) λ_{max} nm ($\varepsilon \cdot 10^{-3}$): 232 (45.9), 355 (30.1), 504 (77.4). IR spectra (KBr), ν, cm^{-1}: 3079 (carborane CH), 2586 (carborane BH), 1570 (C=C, C=N), 1390 (B–O). ^{1}H NMR (400.13 MHz, $CDCl_3$) δ, ppm: 7.70 (d, *J*=8.4 Hz, 2H, Ph), 7.66 (s, 2H, pyrrole), 7.46 (d, *J*=8.4 Hz, 2H, Ph), 6.96 (d, *J*=4.1 Hz, 2H, pyrrole), 6.62 (dd, *J*=4.3, 1.8 Hz, 2H, pyrrole), 4.04 (br. s., 2H, carborane CH), 3.59 (s, 4H, CH_2). ^{11}B NMR (128.28 MHz, $CDCl_3$) δ, ppm: 0.6 (br.s., 1B, O–*B*–*O*), –2.8 to –12.6 (m, 20B). MS (ESI): *m/z* $[M–H]^-$ calcd. for $C_{21}H_{36}B_{21}BrN_2O_2$ 655.4; found 655.3

Compound 11. Yield 150 mg (75 %) as reddish-green crystals. UV–vis (CH_2Cl_2) λ_{max} nm ($\varepsilon \cdot 10^{-3}$): 226 (23.3), 328 (8.0), 378 (6.7), 518 (57.4). IR spectra (KBr), ν, cm^{-1}: 3070 (carborane CH), 2573 (carborane BH), 1569 (C=C, C=N), 1389 (B–O). ^{1}H NMR (400.13 MHz, $CDCl_3$) δ, ppm: 7.72 (s, 2H, pyrrole), 6.87 (dd, *J*=4, 0.8 Hz, 2H, pyrrole), 6.66 (dd, *J*=4.4, 1.9 Hz, 2H, pyrrole), 4.02 (br.s., 2H, carborane CH). 3.64 (s, 4H, CH_2). ^{11}B NMR (128.38 MHz, $CDCl_3$) δ, ppm: 0.6 (br. s., 1B, O-*B*–*O*), –2.8 to –5.9 (m, 4B), –8.7 to –14.0 (m, 16B). ^{19}F NMR (282.40 MHz, $CDCl_3$); δ, ppm: –137.1 (d, *J*=19 Hz, 2F, Ar-F-*ortho*), –148.4 (t, *J* = 22 Hz, 1F, Ar-F-*para*), –158.6 to –158.7 (m, 2F, Ar-F-*meta*). MS (ESI): *m/z* $[M–H]^-$ calcd. for $C_{21}H_{32}B_{21}F_5N_2O_2$ 666.4; found 666.3.

Compound 23. Yield 156 mg (72 %) as reddish-green crystals. UV–vis (CH_2Cl_2) λ_{max} nm ($\varepsilon \cdot 10^{-3}$): 228 (47.7), 318 (15.2), 374 (13.0), 518 (143.1). IR spectra (KBr), ν, cm^{-1}: 3083 (carborane CH), 2590 (carborane BH), 1556 (C=C, C=N), 1299 (B–O). ^{1}H NMR (400.13 MHz, $CDCl_3$) δ, ppm: 6.12 (s, 2H, pyrrole), 4.16 (br.s., 2H, carborane CH), 3.34 (s, 4H, CH_2), 2.46 (s, 6H, CH_3), 1.65 (s, 6H, CH_3). ^{11}B NMR (128.38 MHz, $CDCl_3$) δ, ppm: 1.0 (br.s., 1B, O–*B*–*O*), –3.1 to –12.6 (m, 20B). ^{19}F NMR (282.40 MHz, $CDCl_3$); δ, ppm: –139.7 (dd, *J* = 22, 7 Hz, 2F, Ar-F-*ortho*), –149.5 (t, *J* = 21 Hz, 1F, Ar-F-*para*), –158.7 to –158.9 (m, 2F, Ar-F-*meta*). MS (ESI): *m/z* $[M–H]^-$ calcd. for $C_{25}H_{40}B_{21}F_5N_2O_2$ 722.5; found 722.4.

General procedure for the preparation of carboranyl-BODIPYs 12–20, 24, 25. To a solution of BODIPY **1**–**3** or **21** (0.3 mmol) in dry CH_2Cl_2 (50 mL) $AlCl_3$ (0.6 mmol) was added and reaction mixture was stirred under argon for 20 min at room temperature in the dark. Then carborane **5** (1.5 mmol) was added and reaction mixture was stirred under argon for 10 min at room temperature in the dark. Water (50 mL) was added, and organic phase was washed with water (2x50 mL) and dried over Na_2SO_4. After removal of the solvent *in vacuo*, the products were purified by column chromatography on silica gel using hexane–CH_2Cl_2 system (1:2) as an eluent for compounds **12**, **15**, **18**, CH_2Cl_2 as an eluent for compounds **13**, **16**, **19, 24** and CH_2Cl_2–acetone system (20:1) as an eluent for compounds **14**, **17**, **20, 25**.

Compound 12. Yield 50 mg (39 %) as yellow crystals. UV–vis (CH_2Cl_2) λ_{max} nm ($\varepsilon \cdot 10^{-3}$): 232 (29.4), 346 (21.6), 498 (66.7). IR spectra (KBr), ν, cm^{-1}: 2580 (carborane BH), 1578 (C=C, C=N), 1388 (B–O). ^{1}H NMR (400.13 MHz, $CDCl_3$) δ, ppm: 7.97 (s, 2H, pyrrole), 7.61-7.51 (m, 5H, Ph), 6.97 (d, *J*=4.1 Hz, 2H, pyrrole), 6.59 (dd, *J*=4.3, 1.8 Hz, 2H, pyrrole), 4.41 (s, 4H, CH_2). ^{11}B NMR (128.38 MHz, $CDCl_3$) δ, ppm: 1.5 (br.s., 1B, O–*B*–*O*), –4.5 to –5.5 (m, 2B), –8.0 to –13.0 (m, 8B). MS (ESI): *m/z* $[M–H]^-$ calcd. for $C_{19}H_{25}B_{11}N_2O_2$ 432.3; found 432.3.

Compound 13. Yield 52 mg (34 %) as orange crystals. UV–vis (CH_2Cl_2) λ_{max} nm ($\varepsilon \cdot 10^{-3}$): 232 (38.8), 352 (23.6), 501 (63.4). IR spectra (KBr), ν, cm^{-1}: 2589 (carborane BH), 1568 (C=C, C=N), 1384 (B–O). ^{1}H NMR (400.13 MHz, $CDCl_3$) δ, ppm: 7.97 (s, 2H, pyrrole), 7.67 (d, *J*=8.23 Hz, 2H, Ph), 7.41 (d, *J*=8.23 Hz, 2H, Ph), 6.93 (d, *J*=4.03 Hz, 2H, pyrrole), 6.59 (dd, *J*=4.21, 1.65 Hz, 2H, pyrrole), 4.38 (br. s., 4H, CH_2). ^{11}B NMR (128.28 MHz, $CDCl_3$) δ, ppm: 1.7 (br.s., 1B, O–*B*–*O*), –4.5 to –10.3 (m, 10B). MS (ESI): *m/z* $[M–H]^-$ calcd. for $C_{19}H_{24}B_{11}BrN_2O_2$ 510.2; found 510.2.

Compound 14. Yield 58 mg (37 %) as reddish-green crystals. UV–vis (CH_2Cl_2) λ_{max} nm ($\varepsilon \cdot 10^{-3}$): 226 (28.2), 326 (10.0), 374 (8.7), 516 (62.0). IR spectra (KBr), ν, cm^{-1}: 2579 (carborane BH), 1580 (C=C, C=N), 1390 (B–O). ^{1}H NMR (400.13 MHz, $CDCl_3$) δ, ppm: 8.01 (s, 2H, pyrrole), 6.85 (d, *J*=4.1 Hz, 2H, pyrrole), 6.62 (dd, *J*=4.4, 1.9 Hz, 2H, pyrrole), 4.39 (s, 4H, CH_2). ^{11}B NMR (128.38 MHz, $CDCl_3$) δ, ppm: 1.8 (br.s., 1B, O–*B*–*O*), –4.3 to –10.2 (m, 10B). ^{19}F NMR (282.40 MHz, $CDCl_3$); δ, ppm: –136.7 (d, *J*=19.3 Hz, 2F, Ar-F-*ortho*), –149.1 (t, *J* = 19.2 Hz, 1F, Ar-F-*para*), –159.1 to –159.3 (m, 2F, Ar-F-*meta*). MS (ESI): *m/z* $[M–H]^-$ calcd. for $C_{19}H_{20}B_{11}F_5N_2O_2$ 521.3; found 521.3

Compound 15. Yield 21 mg (16 %) as dark yellow crystals. UV–vis (CH_2Cl_2) λ_{max} nm ($\varepsilon \cdot 10^{-3}$): 230 (19.0), 346 (14.5), 500 (46.1). IR spectra (nujol), ν, cm^{-1}: 3362 (OH). IR spectra (KBr), ν, cm^{-1}: 2581(carborane BH), 1578 (C=C, C=N), 1387 (B–O), 1138 (B–F). ^{1}H NMR (400.13 MHz, $CDCl_3$) δ, ppm: 7.86 (s, 2H, pyrrole), 7.65–7.54 (m, 5H, Ph), 7.01 (d, *J*=4.1 Hz, 2H, pyrrole), 6.62 (dd, *J*=4.1, 1.6 Hz, 2H, pyrrole), 4.15 (br. s., 3H, OH+CH_2), 3.89 (s, 2H, CH_2). ^{11}B NMR (128.38 MHz, $CDCl_3$) δ, ppm: 0.5 (d, *J*=28 Hz, 1B, O–*B*–*F*), –3.1 to –4.2 (m, 2B), –10.6 to –12.4 (m, 8B). MS (ESI): *m/z* $[M–H]^-$ calcd. for $C_{19}H_{26}B_{11}FN_2O_2$ 451.3; found 451.3.

Compound 16. Yield 19 mg (12 %) as dark orange crystals. UV–vis (CH_2Cl_2) λ_{max} nm ($\varepsilon \cdot 10^{-3}$): 234 (46.2), 355 (30.3), 503 (78.0). IR spectra (nujol), ν, cm^{-1}: 3420 (OH). IR spectra (KBr), ν, cm^{-1}: 2580 (carborane BH), 1568 (C=C, C=N), 1384 (B–O), 1148 (B–F). ^1H NMR (400.13 MHz, $CDCl_3$) δ, ppm: 7.87 (s, 2H, pyrrole), 7.72 (d, *J*=8.7 Hz, 2H, Ph), 7.47 (d, *J*=8.4 Hz, 2H, Ph), 6.98 (d, *J*=3.9 Hz, 2H, pyrrole), 6.63 (dd, *J*=4.3, 1.8 Hz, 2H, pyrrole), 4.14 (br. s., 3H, OH+CH_2), 3.89 (br. s., 2H, CH_2). ^{11}B NMR (128.38 MHz, $CDCl_3$) δ, ppm: 0.5 (d, *J*=28 Hz, 1B, F–*B*–*O*), –3.0 to –4.2 (m, 2B), –8.0 to –12.3 (m, 8B). MS (ESI): *m/z* $[M–H]^-$ calcd. for $C_{19}H_{25}B_{11}BrFN_2O_2$ 529.2; found 529.2.

Compound 17. Yield 24 mg (15 %) as dark reddish-green crystals.

UV–vis (CH_2Cl_2) λ_{max} nm ($\varepsilon \cdot 10^{-3}$): 224 (21.7), 328 (6.5), 378 (6.0), 520 (48.9). IR spectra (nujol), ν, cm^{-1}: 3373 (OH). IR spectra (KBr), ν, cm^{-1}: 2585 (carborane BH), 1571 (C=C, C=N), 1389 (B–O), 1186 (B–F). ^1H NMR (400.13 MHz, $CDCl_3$) δ, ppm: 7.92 (s, 2H, pyrrole), 6.88 (d, *J*=4.1 Hz, 2H, pyrrole), 6.66 (dd, *J*=4.3, 1.4 Hz, 2H, pyrrole), 4.13 (s, 2H, CH_2), 4.10 (br.s., 1H, OH). 3.88 (s, 2H, CH_2). ^{11}B NMR (128.38 MHz, $CDCl_3$) δ, ppm: 0.5 (d, *J*=28 Hz, 1B, F–*B*–*O*), –2.8 to –4.3 (m, 2B), –9.7 to –12.4 (m, 8B). ^{19}F NMR (282.40 MHz, $CDCl_3$); δ, ppm: –136.7 to –136.9 (m, 2F, Ar-F-*ortho*), –148.4 (t, *J* = 19.2 Hz, 1F, Ar-F-*para*), –152.9 to –153.2 (m, 1F, B–F), –158.7 to –158.8 (m, 2F, Ar-F-*meta*). MS (ESI): *m/z* $[M–H]^-$ calcd. for $C_{19}H_{21}B_{11}F_6N_2O_2$ 542.3; found 542.2

Compound 18. Yield 53 mg (28 %) as dark yellow crystals. UV–vis (CH_2Cl_2) λ_{max} nm ($\varepsilon \cdot 10^{-3}$): 232 (22.5), 352 (17.8), 502 (54.3). IR spectra (nujol), ν, cm^{-1}: 3403 (OH). IR spectra (KBr), ν, cm^{-1}: 2586 (carborane BH), 1578 (C=C, C=N), 1389 (B–O).^1H NMR (400.13 MHz, $CDCl_3$) δ, ppm: 7.77 (s, 2H, pyrrole), 7.69-7.57 (m, 5H, Ph), 7.08 (d, *J*=4.1 Hz, 2H, pyrrole), 6.70 (dd, *J*=4.1, 1.9 Hz, 2H, pyrrole), 4.39 (br. s., 2H, OH), 4.17 (s, 4H, CH_2), 3.70 (s, 4H, CH_2). ^{11}B NMR (128.38 MHz, $CDCl_3$) δ, ppm: 0.5 (br. s., 1B, O–*B*–*O*), –1.5 to –4.2 (m, 4B), –7.3 to –13.2 (m, 16B). MS (ESI): *m/z* $[M–H]^-$ calcd. for $C_{23}H_{41}B_{21}N_2O_4$ 635.5; found 635.5.

Compound 19. Yield 49 mg (23 %) as dark orange crystals. UV–vis (CH_2Cl_2) λ_{max} nm ($\varepsilon \cdot 10^{-3}$): 234 (45.8), 360 (30.9), 505 (74.4). IR spectra (nujol), ν, cm^{-1}: 3406 (OH). IR spectra (KBr), ν, cm^{-1}: 2585 (carborane BH), 1567 (C=C, C=N), 1384 (B–O). ^{1}H NMR (400.13 MHz, $CDCl_3$) δ, ppm: 7.78 (s, 2H, pyrrole), 7.74 (d, *J*=8.3 Hz, 2H, Ph), 7.50 (d, *J*=8.6 Hz, 2H, Ph), 7.04 (d, *J*=4.1 Hz, 2H, pyrrole), 6.70 (dd, *J*=4.3, 1.8 Hz, 2H, pyrrole), 4.42 (br. s., 2H, OH), 4.15 (s, 4H, CH_2), 3.69 (s, 4H, CH_2). ^{11}B NMR (128.38 MHz, $CDCl_3$) δ, ppm: 0.5 (br.s., 1B, O–*B*–*O*), –3.1 to –4.1 (m, 4B), –7.9 to –14.4 (m, 16B). MS (ESI): *m/z* [M–H]$^-$ calcd. for $C_{23}H_{40}B_{21}BrN_2O_4$ 713.4; found 713.3.

Compound 20. Yield 56 mg (26%) as dark reddish-green crystals. UV–vis (CH_2Cl_2) λ_{max} nm ($\varepsilon \cdot 10^{-3}$): 226 (28.3), 276 (10.9), 334 (11.6), 520 (53.8). IR spectra (nujol), ν, cm^{-1}: 3446 (OH). IR spectra (KBr), ν, cm^{-1}: 2584 (carborane BH), 1572 (C=C, C=N), 1389 (B–O). ^{1}H NMR (400.13 MHz, $CDCl_3$) δ, ppm: 7.84 (s, 2H, pyrrole), 6.93 (dd, *J*=3.7, 0.8 Hz, 2H, pyrrole), 6.72 (d, *J*=3.2 Hz, 2H, pyrrole), 4.27 (br.s., 2H, OH), 4.15 (s, 4H, CH_2), 3.74 (s, 4H, CH_2). ^{11}B NMR (128.28 MHz, $CDCl_3$) δ, ppm: 0.5 (br.s., 1B, O–*B*–*O*), –3.0 to –12.3 (m, 20B). ^{19}F NMR (282.40 MHz, $CDCl_3$); δ, ppm: –137.0 (d, *J* = 16 Hz, 2F, Ar-F-*ortho*), –147.8 (t, *J* = 22 Hz, 1F, Ar-F-*para*), –158.3 to –158.4 (m, 2F, Ar-F-*meta*). MS (ESI): *m/z* [M–H]$^-$ calcd. for $C_{23}H_{36}B_{21}F_5N_2O_4$ 726.5; found 726.4.

Compound 24. Yield 61 mg (34 %) as dark reddish-green crystals. UV–vis (CH_2Cl_2) λ_{max} nm ($\varepsilon \cdot 10^{-3}$): 228 (44.8), 316 (14.3), 376 (14.2), 518 (156.0). IR spectra (nujol), ν, cm^{-1}: 3446 (OH). IR spectra (KBr), ν, cm^{-1}: 2591 (carborane BH), 1554 (C=C, C=N), 1369 (B–O), 1187 (B–F). ^{1}H NMR (400.13 MHz, $CDCl_3$) δ, ppm: 6.13 (s, 2H, pyrrole), 4.27 (br.s., 1H, OH), 4.10 (br.s., 2H, CH_2), 3.56 (s, 2H, CH_2), 2.55 (s, 6H, CH_3), 1.65 (s, 6H, CH_3). ^{11}B NMR (128.38 MHz, $CDCl_3$) δ, ppm: 0.9 (d, *J* = 23 Hz, 1B, F–*B*–*O*), –3.2 to –4.2 (m, 2B), –10.6 to –12.7 (m, 8B). ^{19}F NMR (282.40 MHz, $CDCl_3$); δ, ppm: –139.5 (d, *J* = 16 Hz, 1F, Ar-F-*ortho*), –140.1 (d, *J* = 16 Hz, 1F, Ar-F-*ortho*), –149.7 (br.s., 1F, Ar-F-*para*), –154.7 to –154.9 (m, 1F,

B–F), –158.7 (br.s., 1F, Ar-F- *meta*), –159.1 (br.s., 1F, Ar-F- *meta*). MS (ESI): *m/z* $[M–H]^-$ calcd. for $C_{23}H_{29}B_{11}F_6N_2O_2$ 597.3; found 597.2.

Compound 25. Yield 110 mg (47 %) as dark reddish-green crystals. UV–vis (CH_2Cl_2) λ_{max} nm ($\varepsilon \cdot 10^{-3}$): 228 (46.9), 316 (16.4), 374 (14.1), 518 (140.8). IR spectra (nujol), ν, cm^{-1}: 3419 (OH). IR spectra (KBr), ν, cm^{-1}: 2590 (carborane BH), 1557 (C=C, C=N), 1297 (B–O). ^1H NMR (400.13 MHz, $CDCl_3$) δ, ppm: 6.19 (s, 2H, pyrrole), 4.20 (br.s., 2H, OH), 4.16 (br.s., 4H, CH_2), 3.62 (s, 4H, CH_2), 2.60 (s, 6H, CH_3), 1.67 (s, 6H, CH_3). ^{11}B NMR (128.38 MHz, $CDCl_3$) δ, ppm: 0.9 (s, 1B, O–*B*–*O*), –3.0 to –4.2 (m, 4B), –7.7 to –11.4 (m, 16B). ^{19}F NMR (282.40 MHz, $CDCl_3$); δ, ppm: –139.7 (br.s., 2F, Ar-F-*ortho*), –149.1 (br.s., 1F, Ar-F-*para*), 158.6 (br.s., 2F, Ar-F- *meta*). MS (ESI): *m/z* $[M–H]^-$ calcd. for $C_{27}H_{44}B_{21}F_5N_2O_4$ 780.5; found 780.4

References

1. APEX II software package, Bruker AXS Inc., 5465, East Cheryl Parkway, Madison, WI 5317, 2005.
2. Sheldrick, G. M. *Acta Crystallogr. Sect. A: Found Crystallogr.* **2008,** *64,* 112–122. doi: 10.1107/S0108767307043930
3. Dolomanov, O. V.; Bourhis, L. J.; Gildea, R. J.; Howard, J. A. K.; Puschmann, H. *J. Appl. Crystallogr.* **2009,** *42,* 339–341. doi: 10.1107/S0021889808042726

Table S1. Crystal data and structure refinement for compounds **8**, **11**, **14**, **18** and **23**.

**Parameter**	**8**	**11**	**14**	**18**	**23**
Empirical formula	$C_{18}H_{19}B_{11}F_6N_2O$	$C_{21}H_{32}B_{21}F_5N_2O_2$	$C_{19}H_{20}B_{11}F_5N_2O_2$	$C_{23}H_{41}B_{21}N_2O_4$	$C_{25}H_{40}B_{21}F_5N_2O_2$
Formula weight	512.26	666.49	522.28	636.59	722.60
Temperature/K	120	120	120	120.00	120
Crystal system	monoclinic	monoclinic	monoclinic	orthorhombic	triclinic
Space group	$P2_1/c$	$P2_1/c$	$P2_1/c$	$Pna2_1$	P-1
a/Å	9.6053(6)	17.7255(6)	14.7686(17)	19.4534(9)	11.7666(4)
b/Å	12.1336(8)	13.6675(5)	13.7548(16)	13.6547(6)	13.3548(5)
c/Å	20.5939(12)	14.1852(5)	12.1603(15)	13.2701(5)	14.3432(5)
α/°	90	90	90	90	70.6480(10)
β/°	100.081(2)	102.8740(10)	102.786(6)	90	68.8360(10)
γ/°	90	90	90	90	71.6570(10)
Volume/Å^3	2363.1(3)	3350.2(2)	2409.0(5)	3524.9(3)	1933.77(12)
Z	4	4	4	4	2
ρ_{calc}g/cm^3	1.440	1.321	1.440	1.200	1.241
μ/mm^{-1}	0.114	0.091	0.110	0.068	0.084
F(000)	1032.0	1352.0	1056.0	1320.0	740.0
Crystalsize/mm^3	0.31 × 0.14 × 0.02	0.34 × 0.14 × 0.04	0.22 × 0.12 × 0.07	0.21 × 0.14 × 0.03	0.25 × 0.14 × 0.11

Radiation	MoKα (λ = 0.71073)	MoKα (λ = 0.71073)	MoKα (λ = 0.71073)	MoKα (λ = 0.71073)	MoKα (λ = 0.71073)
2Θ range for data collection/°	3.912 to 55.72	3.8 to 51.996	4.094 to 55.056	3.644 to 51.978	3.314 to 59.43
Index ranges	-12 ≤ h ≤ 12, -15 ≤ k ≤ 15, -26 ≤ l ≤ 26	-21 ≤ h ≤ 21, -16 ≤ k ≤ 16, -17 ≤ l ≤ 17	-19 ≤ h ≤ 19, -17 ≤ k ≤ 17, -15 ≤ l ≤ 15	-18 ≤ h ≤ 23, -16 ≤ k ≤ 16, -16 ≤ l ≤ 16	-16 ≤ h ≤ 16, -18 ≤ k ≤ 18, -19 ≤ l ≤ 19
Reflections collected	26920	47552	26191	36592	33318
Independent reflections	5570 [R_{int} = 0.0301, R_{sigma} = 0.0314]	6573 [R_{int} = 0.0372, R_{sigma} = 0.0298]	5506 [R_{int} = 0.1215, R_{sigma} = 0.1561]	6897 [R_{int} = 0.0513, R_{sigma} = 0.0536]	10846 [R_{int} = 0.0287, R_{sigma} = 0.0391]
Data/restraints/parameters	5570/0/351	6573/0/460	5506/0/353	6897/1/453	10846/0/500
Goodness-of-fit on F^2	1.086	1.058	0.980	1.006	1.037
Final R indexes [I>=2σ (I)]	R_1 = 0.0468, wR_2 = 0.1452	R_1 = 0.0406, wR_2 = 0.1050	R_1 = 0.0558, wR_2 = 0.1219	R_1 = 0.0443, wR_2 = 0.1047	R_1 = 0.0545, wR_2 = 0.1737
Final R indexes [all data]	R_1 = 0.0574, wR_2 = 0.1493	R_1 = 0.0556, wR_2 = 0.1121	R_1 = 0.1433, wR_2 = 0.1556	R_1 = 0.0579, wR_2 = 0.1089	R_1 = 0.0691, wR_2 = 0.1816
Largest diff. peak/hole / e $Å^{-3}$	0.70/-0.50	0.28/-0.25	0.28/-0.27	0.23/-0.18	0.71/-0.58

NMR spectra of synthesized compounds 6–20 and 22–25

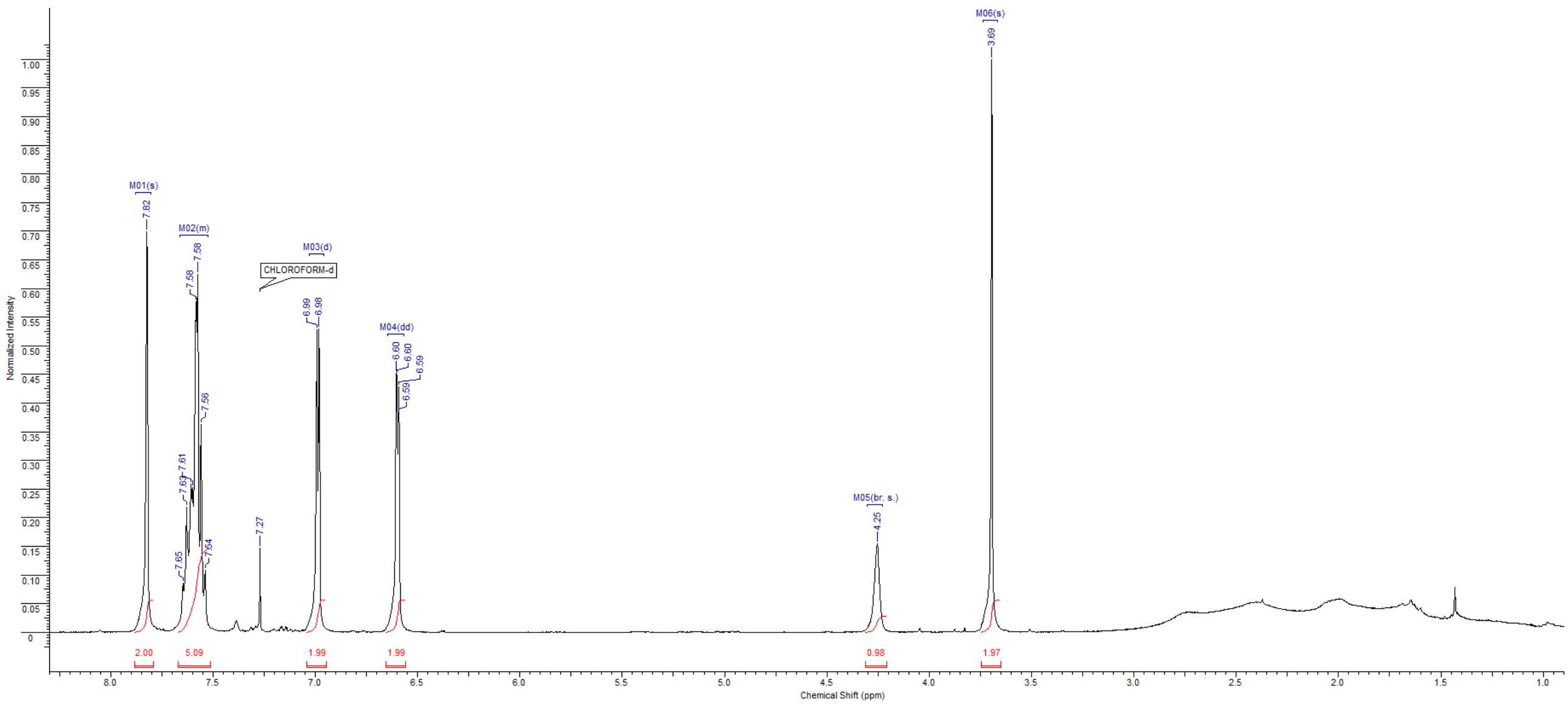

Figure S1: ^{1}H NMR (400 MHz, $CDCl_3$) spectrum of compound **6**.

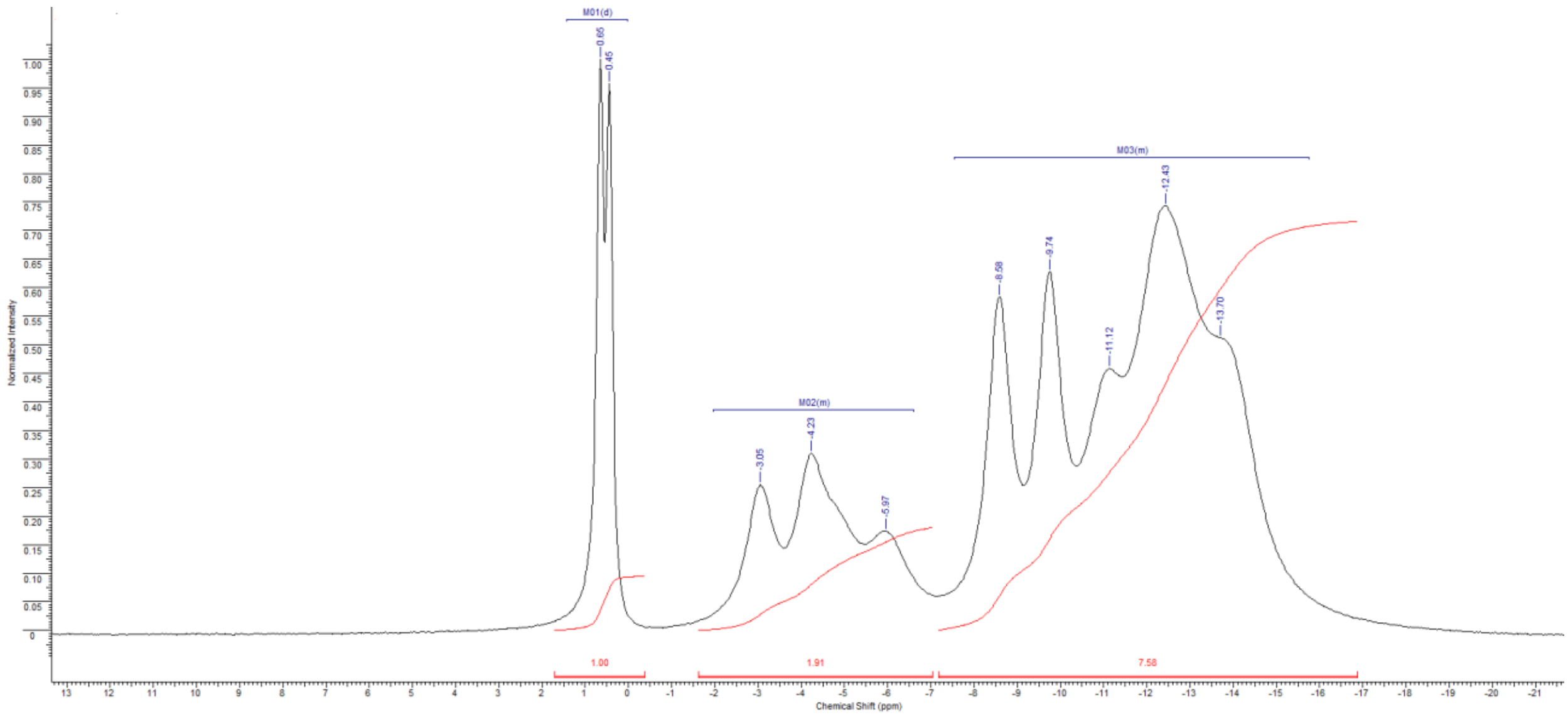

Figure S2: ^{11}B NMR (128 MHz, $CDCl_3$) spectrum of compound **6**.

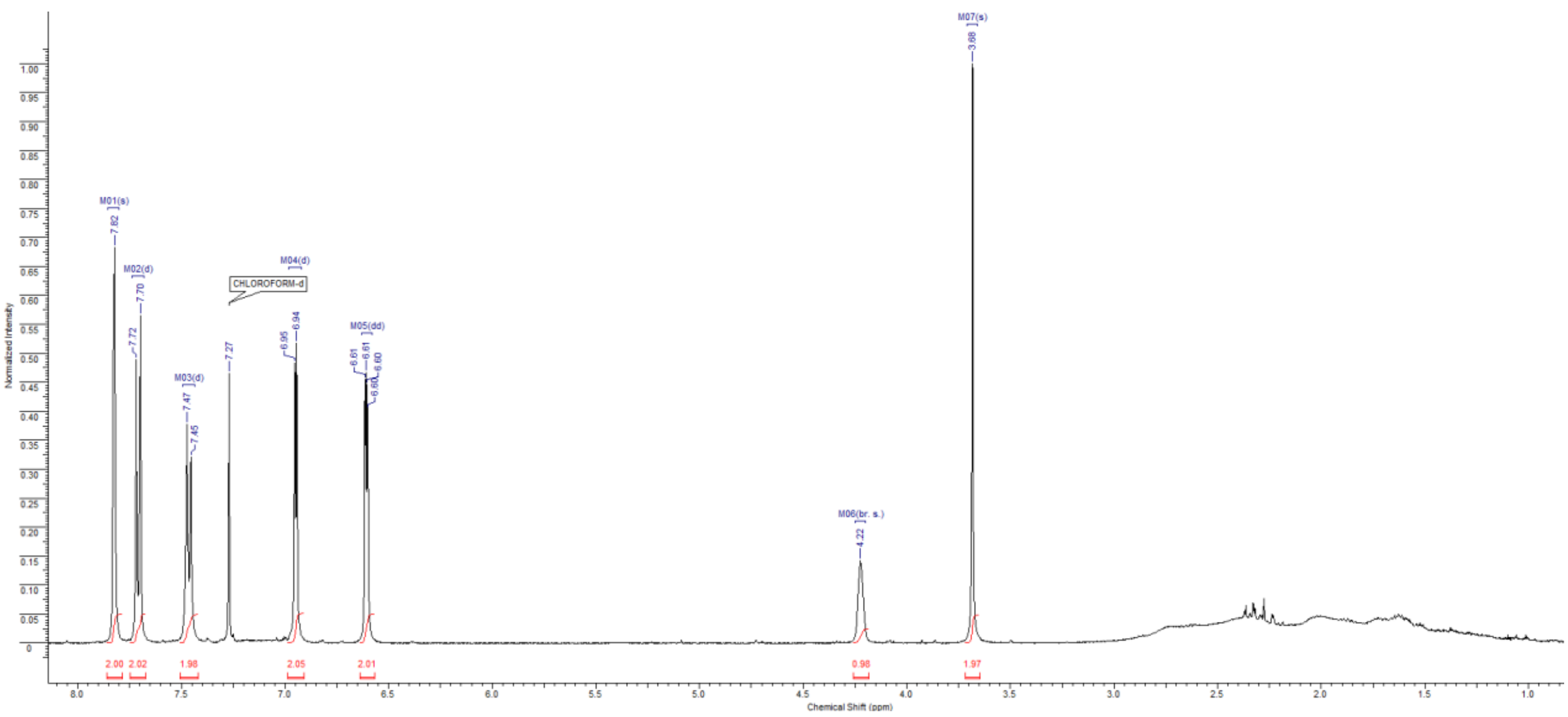

Figure S3: ^{1}H NMR (400 MHz, $CDCl_3$) spectrum of compound **7**.

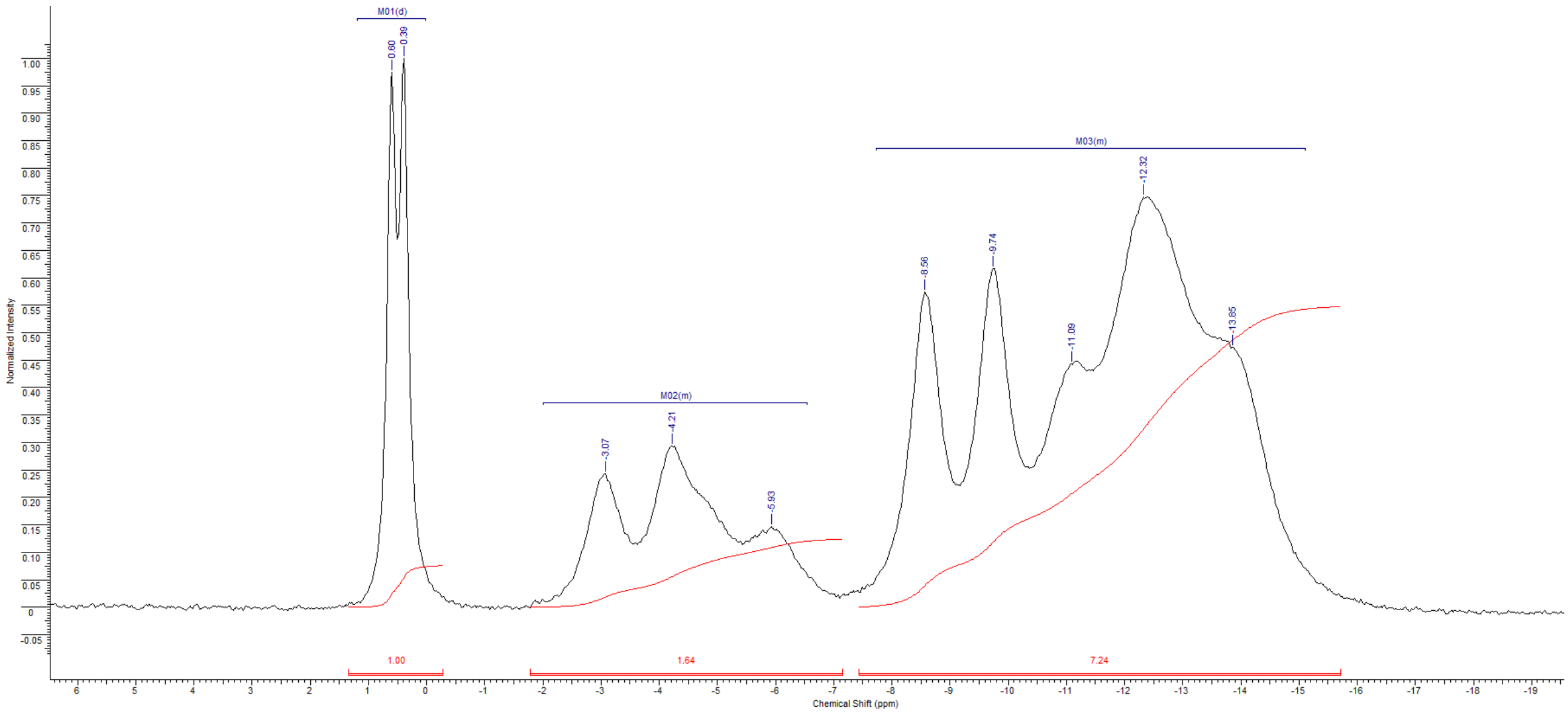

Figure S4: ^{11}B NMR (128 MHz, $CDCl_3$) spectrum of compound **7**.

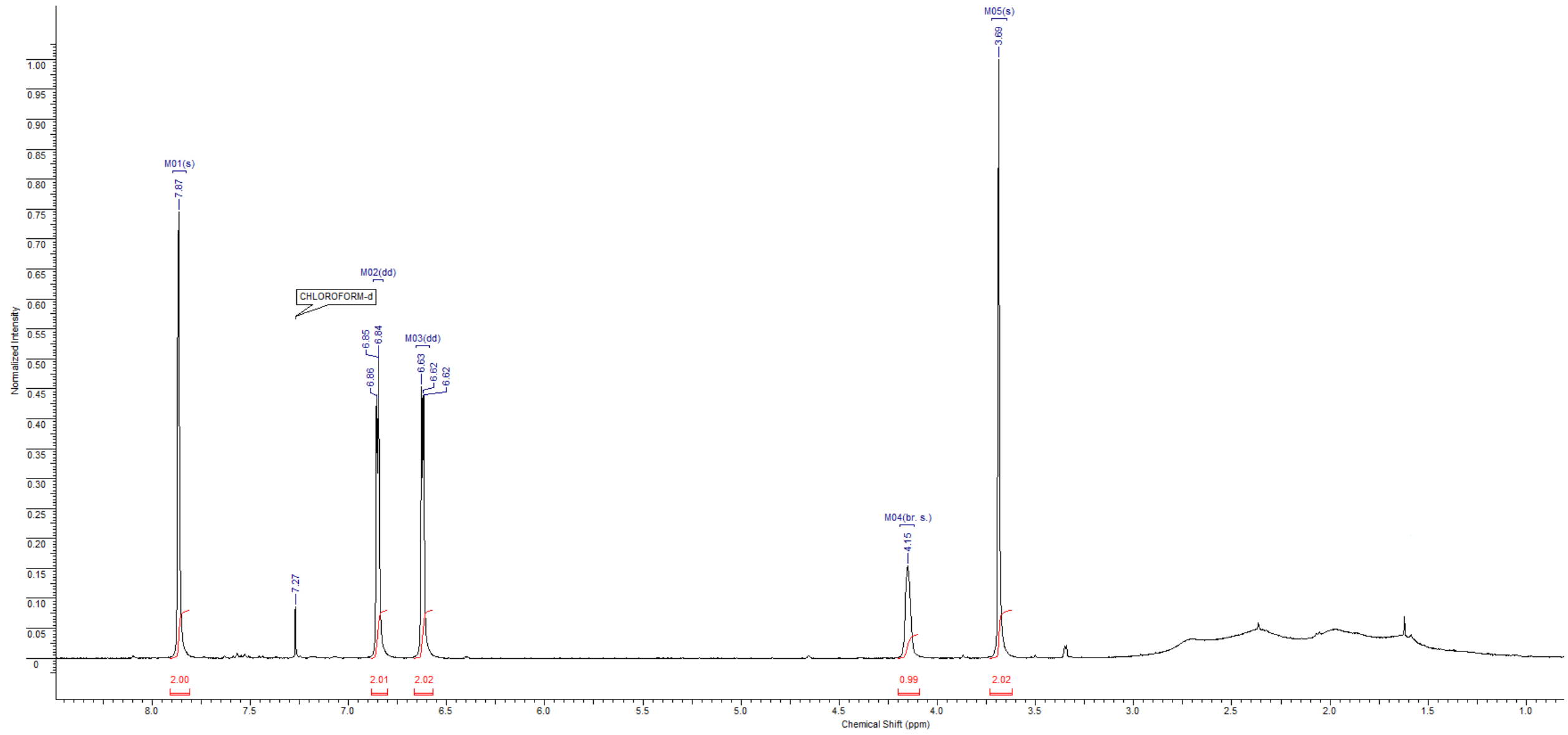

Figure S5: ^{1}H NMR (400 MHz, $CDCl_3$) spectrum of compound **8**.

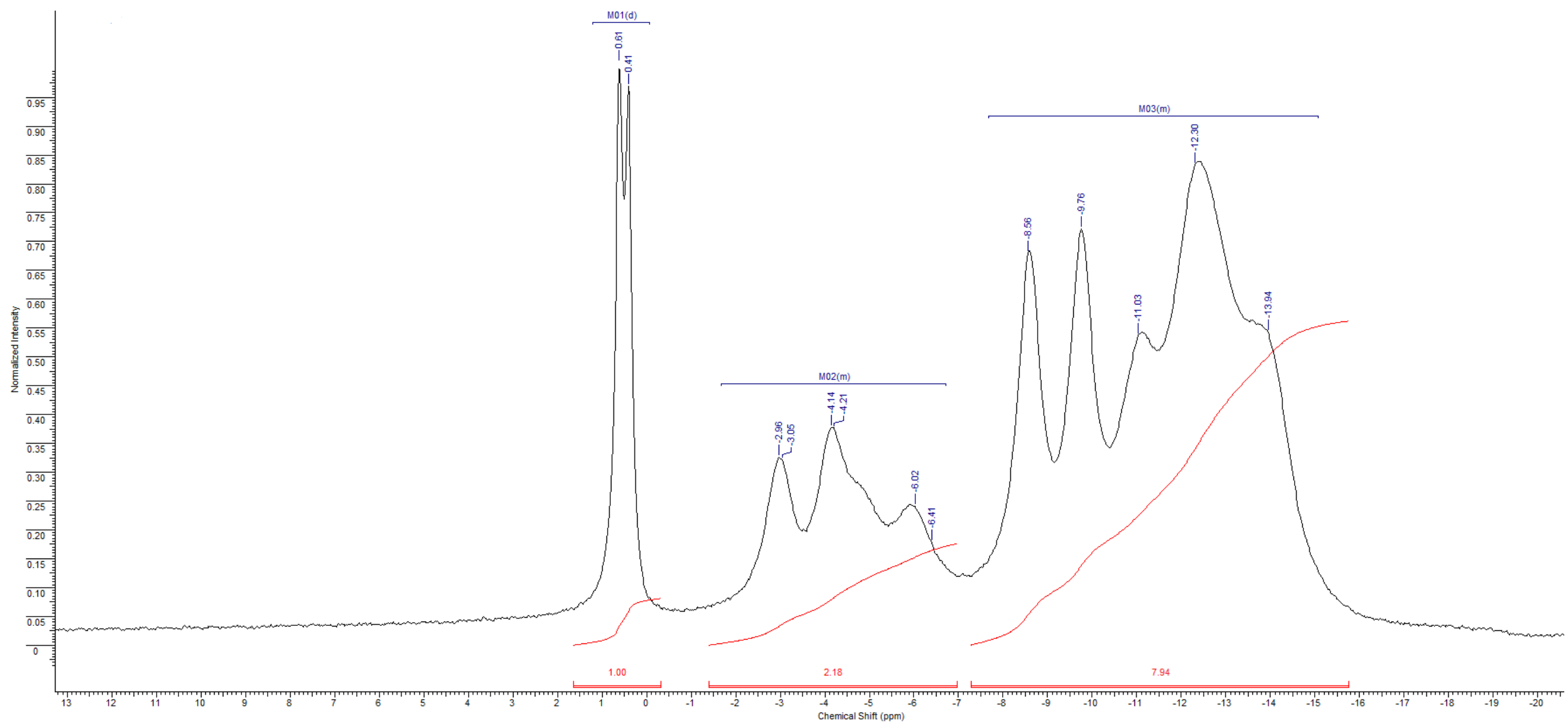

Figure S6: ^{11}B NMR (128 MHz, $CDCl_3$) spectrum of compound **8**.

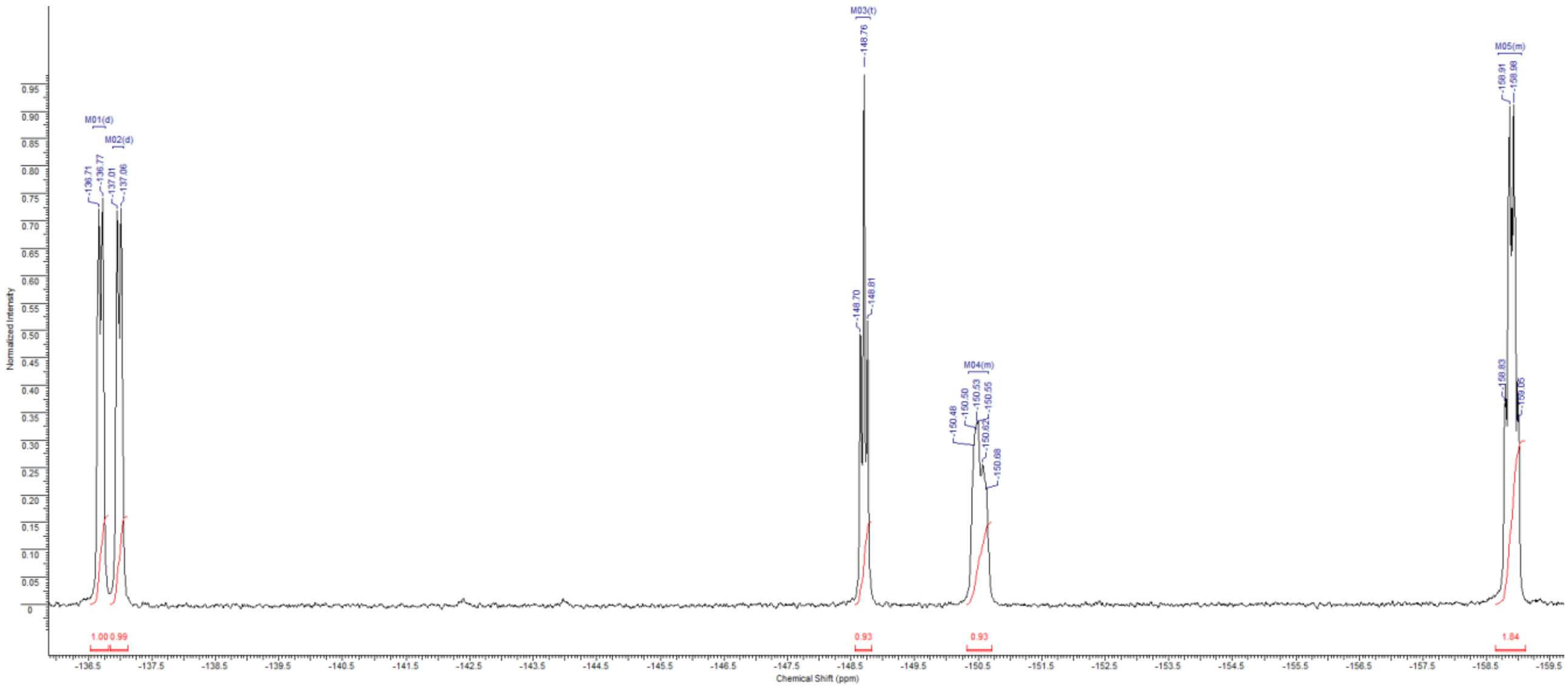

Figure S7: ^{19}F NMR (376 MHz, $CDCl_3$) spectrum of compound **8**.

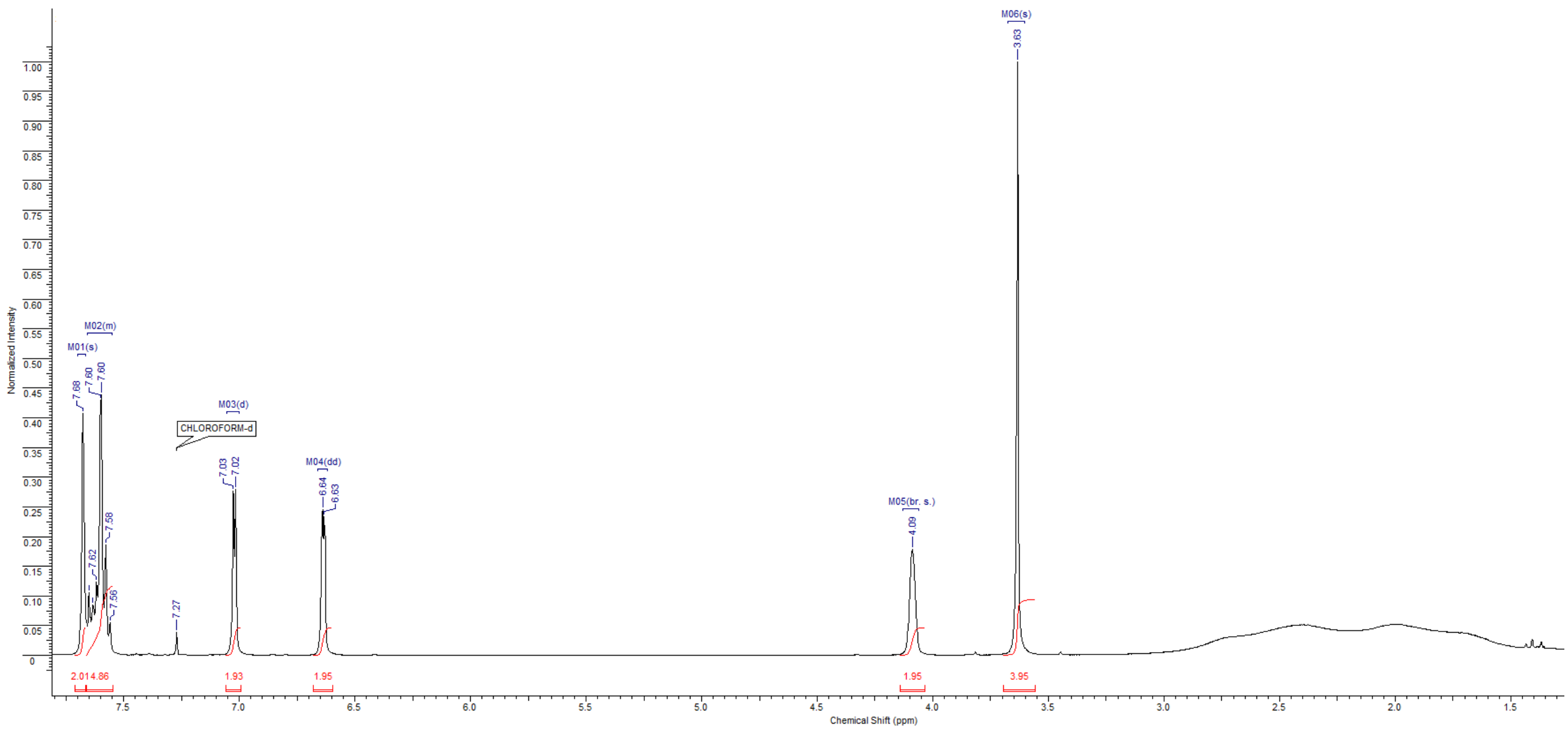

Figure S8: ^{1}H NMR (400 MHz, $CDCl_3$) spectrum of compound **9**.

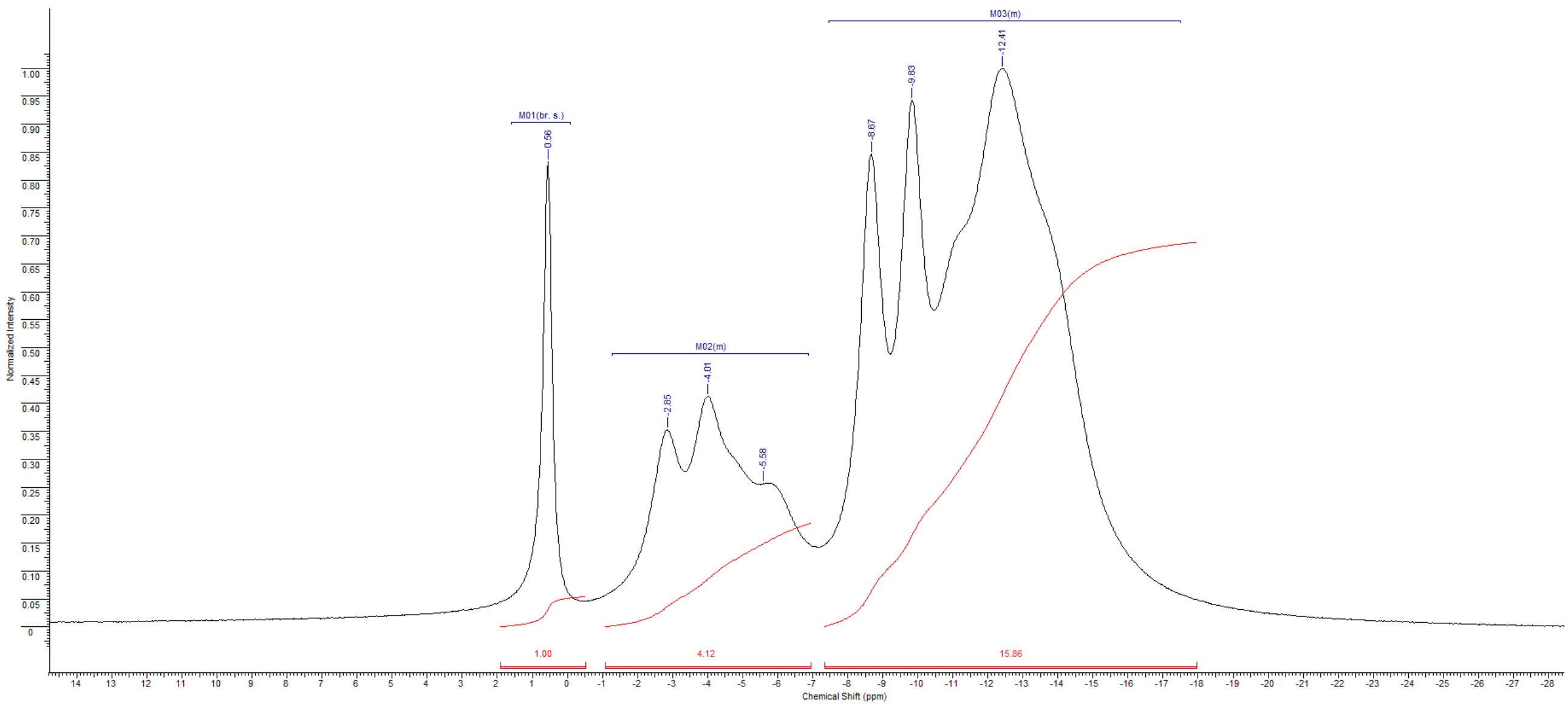

Figure S9: ^{11}B NMR (128 MHz, $CDCl_3$) spectrum of compound **9**.

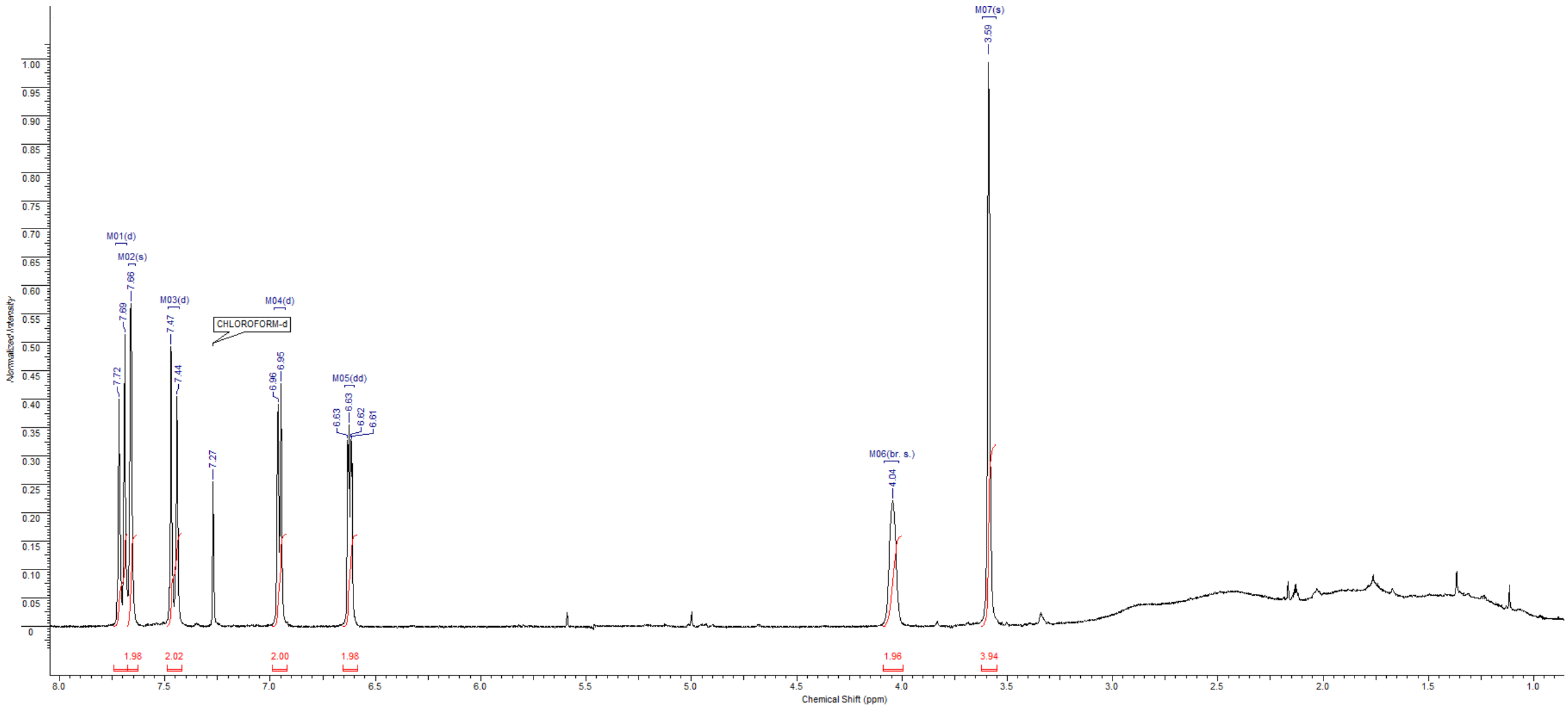

Figure S10: ^{1}H NMR (400 MHz, $CDCl_3$) spectrum of compound **10**.

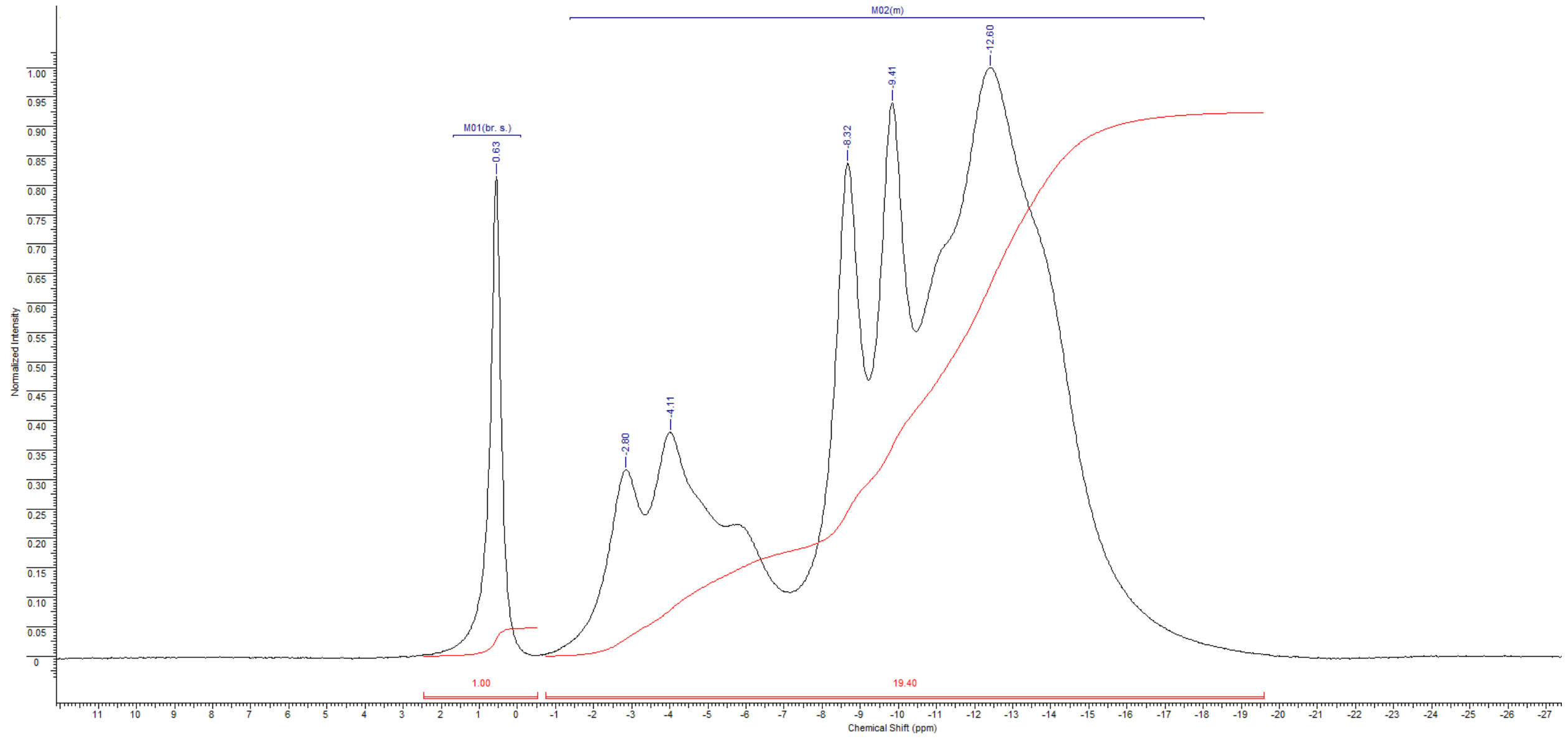

Figure S11: ^{11}B NMR (128 MHz, $CDCl_3$) spectrum of compound **10**.

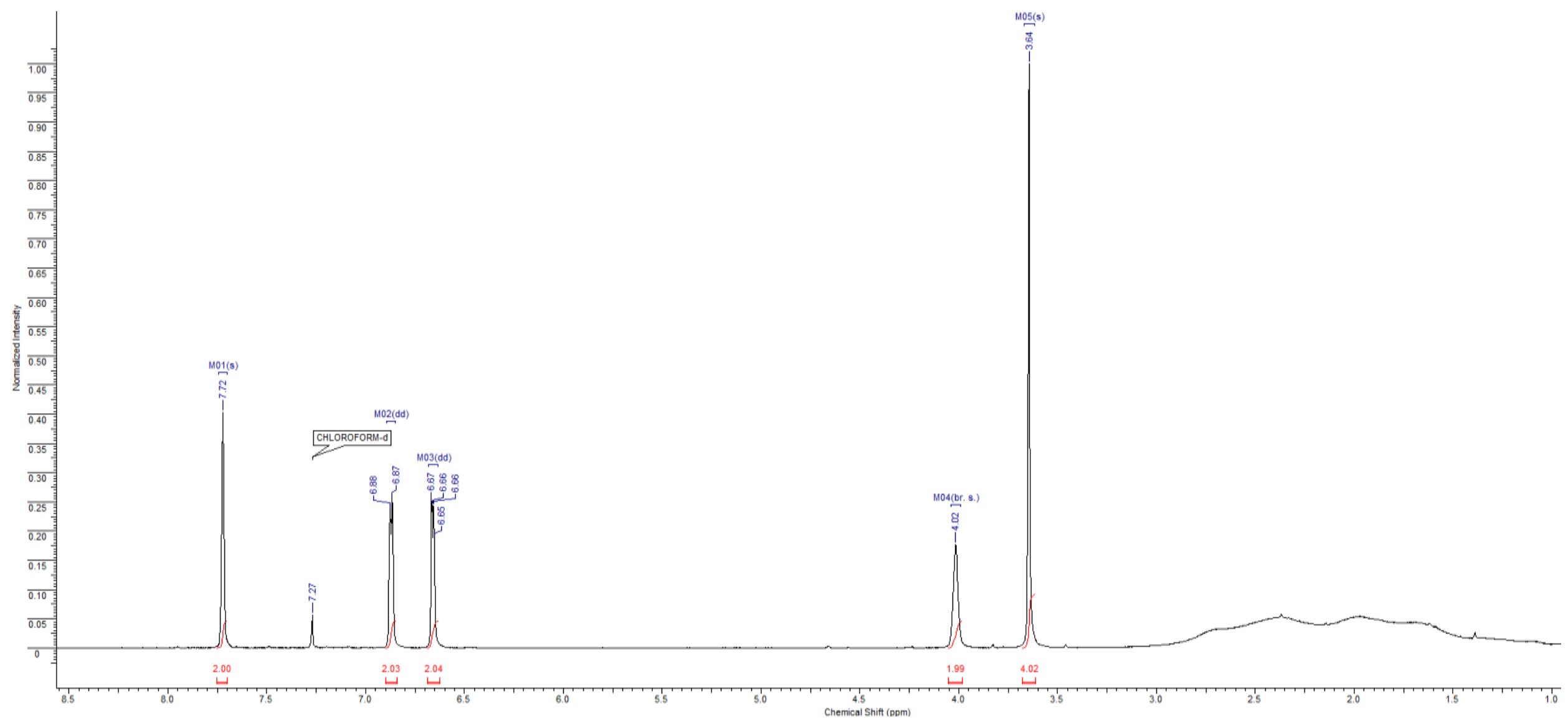

Figure S12: ^{1}H NMR (400 MHz, $CDCl_3$) spectrum of compound **11**.

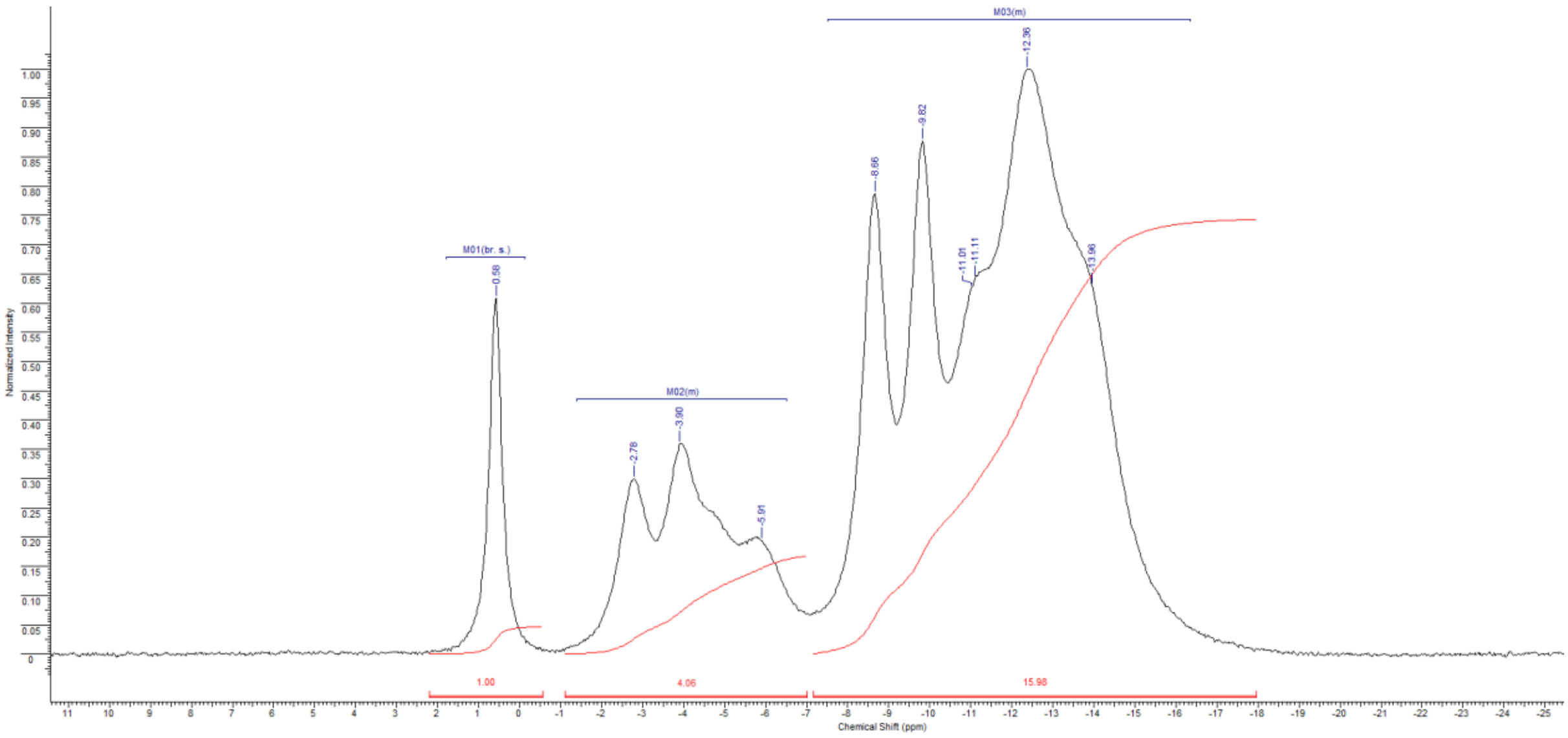

Figure S13: ^{11}B NMR (128 MHz, $CDCl_3$) spectrum of compound **11**

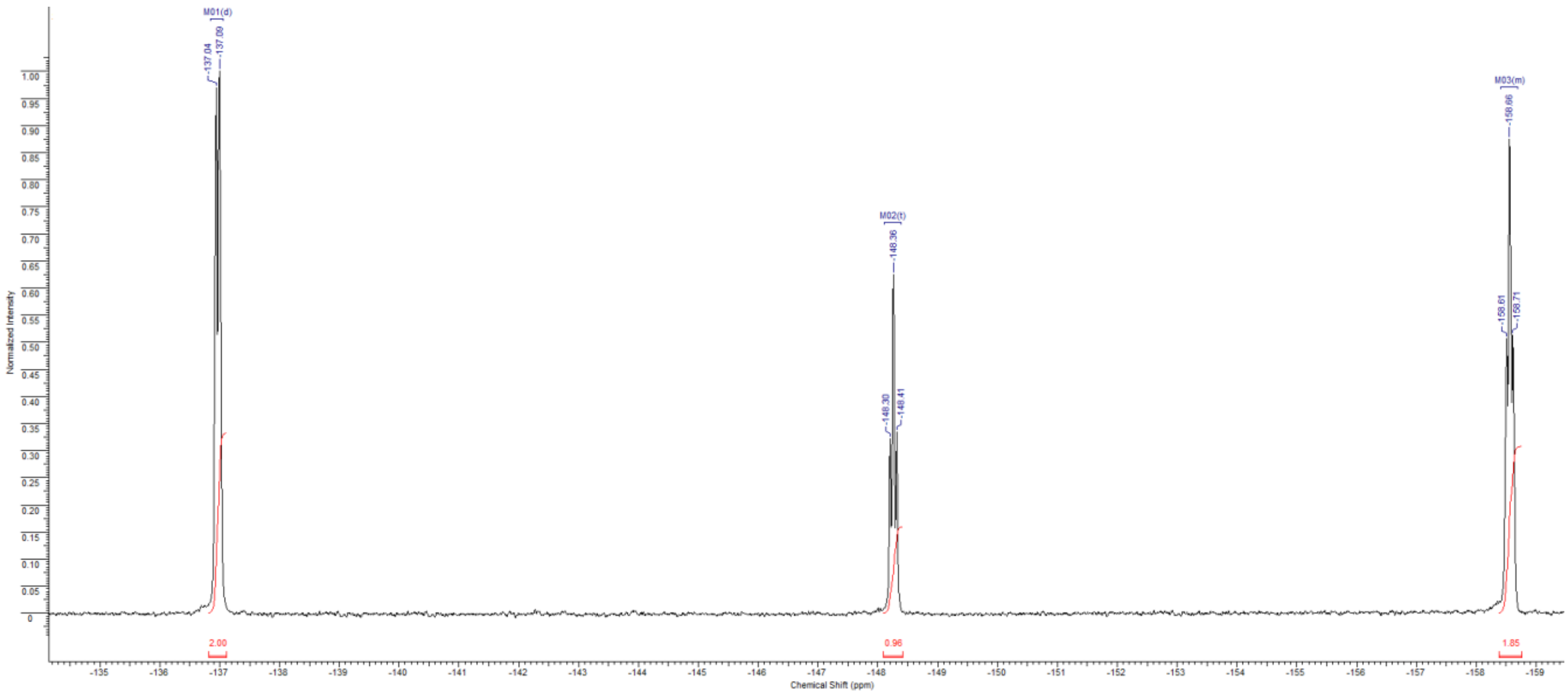

Figure S14: ^{19}F NMR (376 MHz, $CDCl_3$) spectrum of compound **11**.

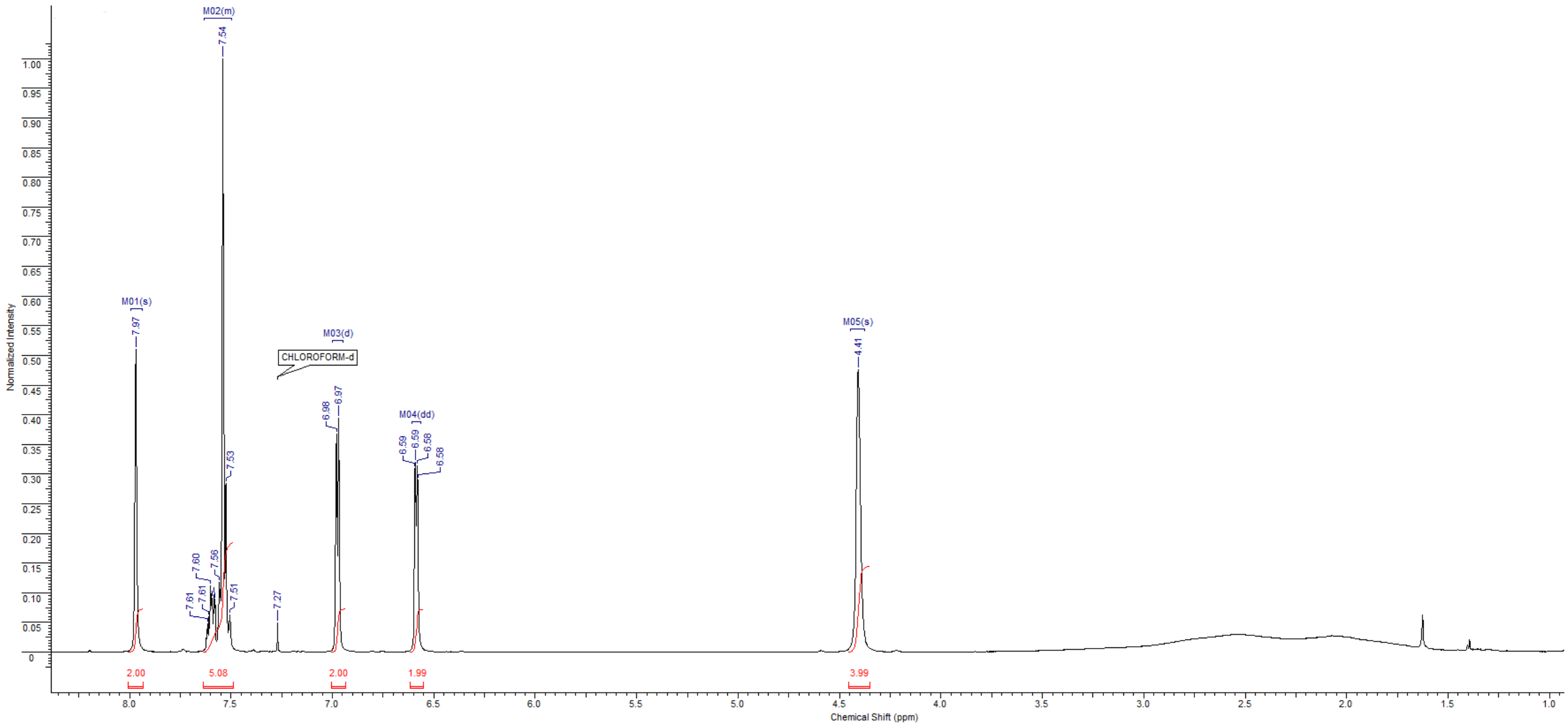

Figure S15: ^{1}H NMR (400 MHz, $CDCl_3$) spectrum of compound **12**.

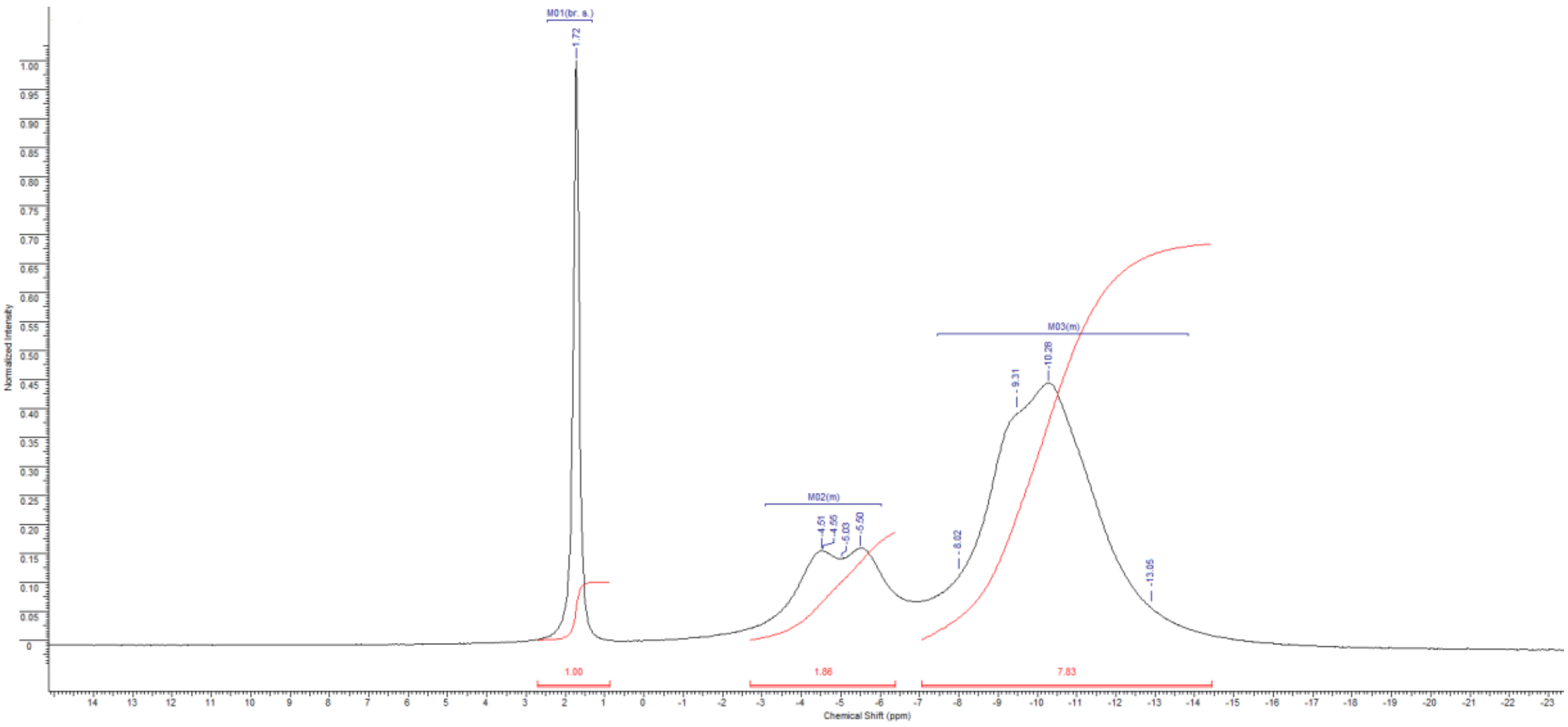

Figure S16: ^{11}B NMR (128 MHz, $CDCl_3$) spectrum of compound **12**.

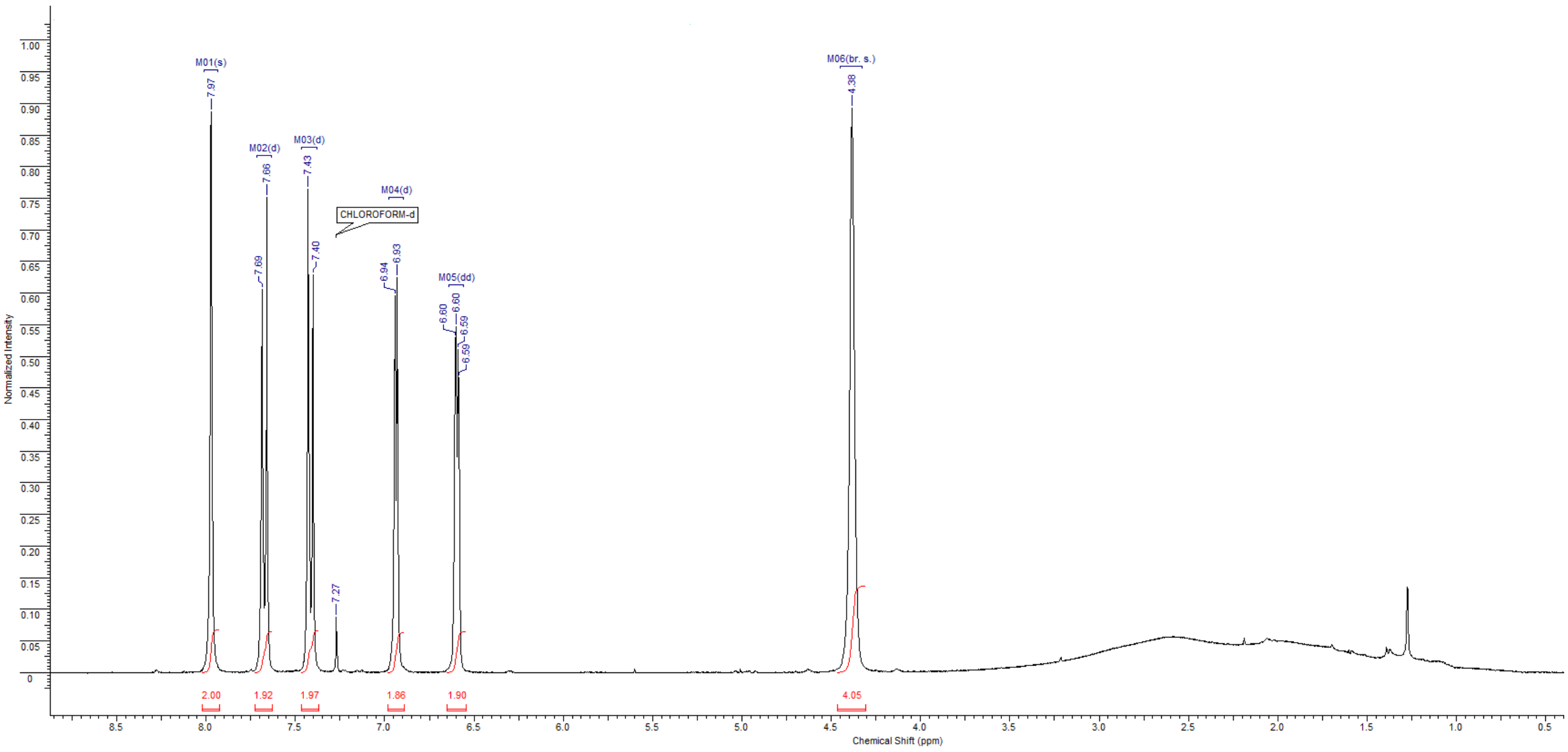

Figure S17: ^{1}H NMR (400 MHz, $CDCl_3$) spectrum of compound **13**.

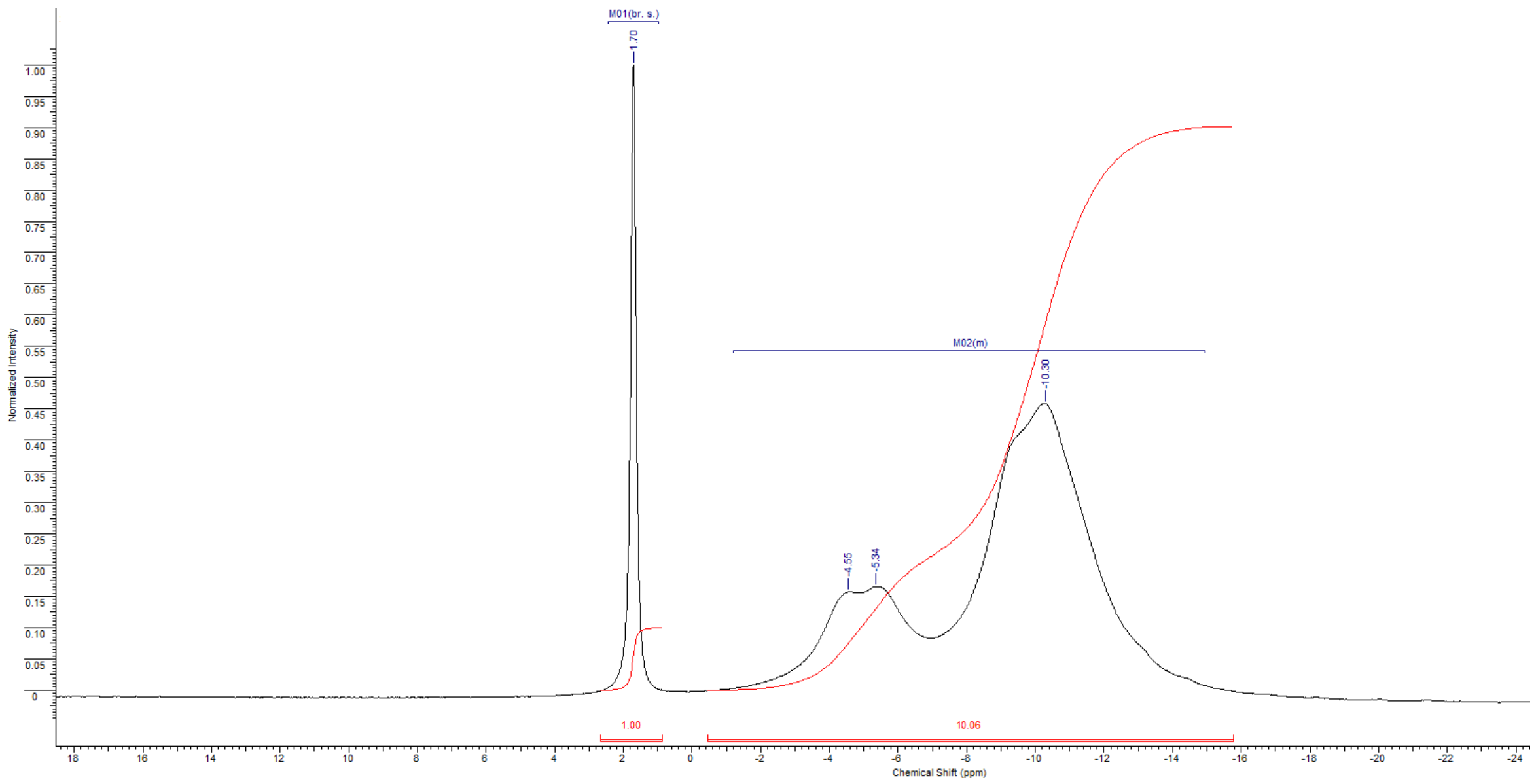

Figure S18: ^{11}B NMR (128 MHz, $CDCl_3$) spectrum of compound **13**.

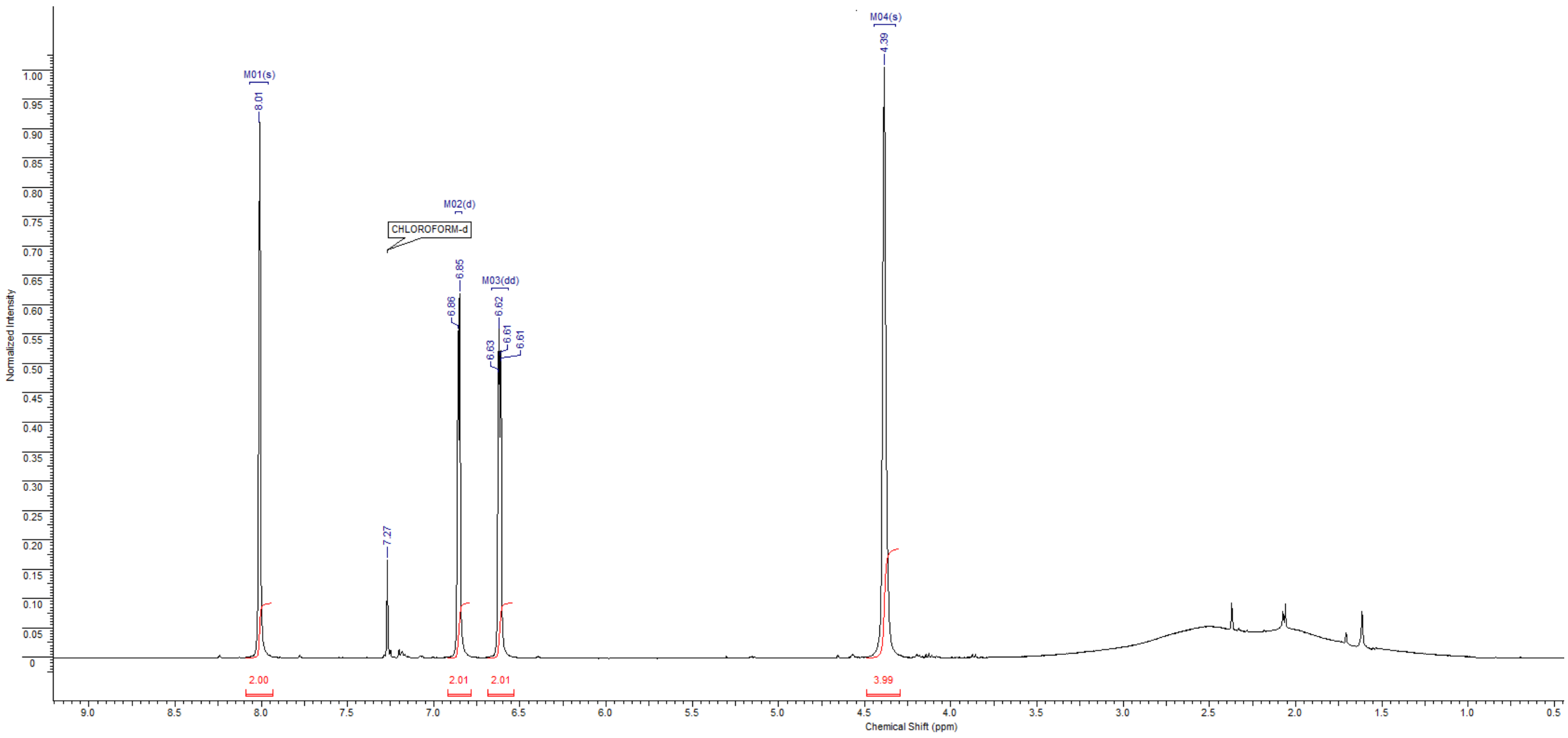

Figure S19: ^{1}H NMR (400 MHz, $CDCl_3$) spectrum of compound **14**.

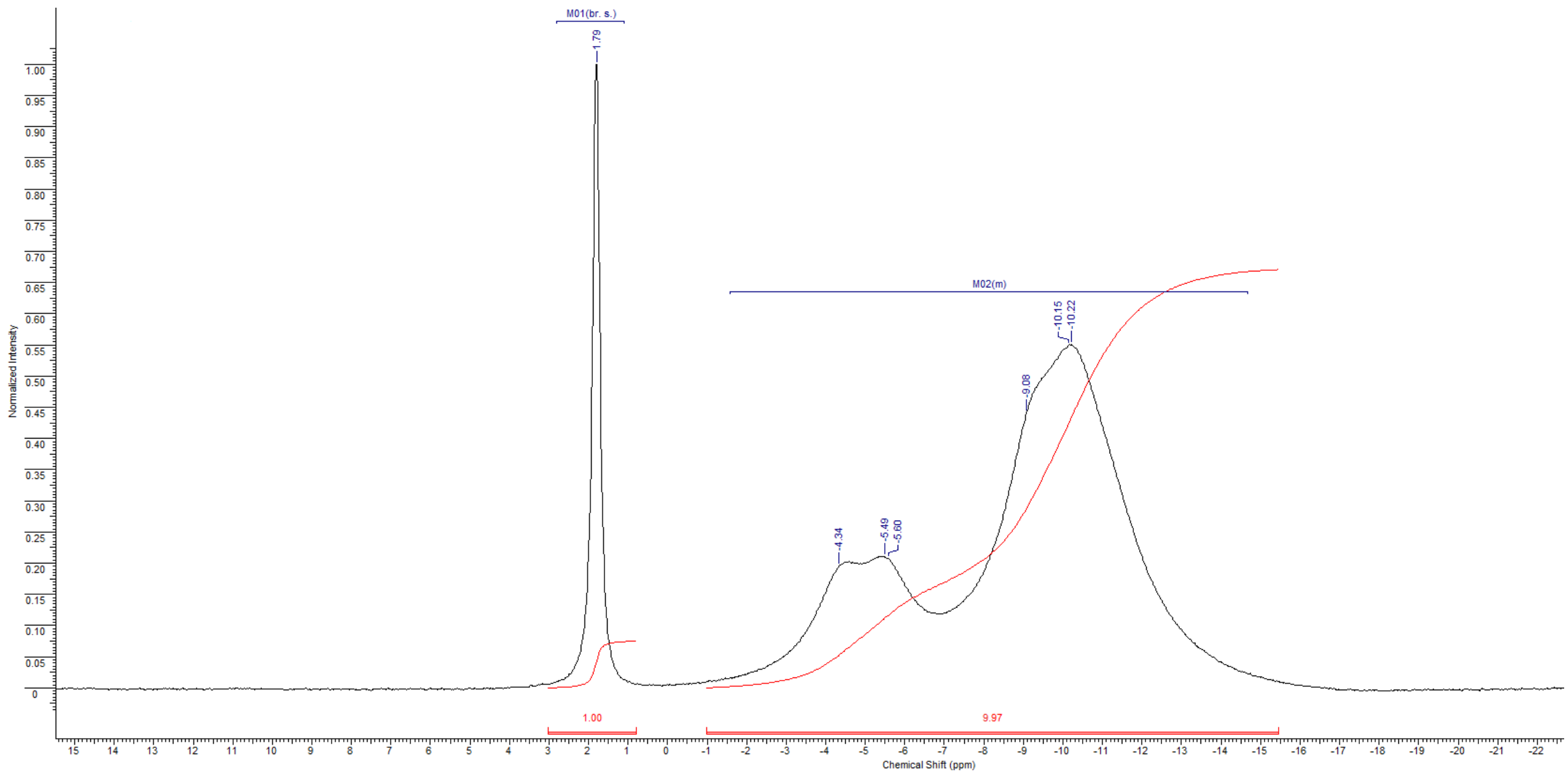

Figure S20: ^{11}B NMR (128 MHz, $CDCl_3$) spectrum of compound **14**.

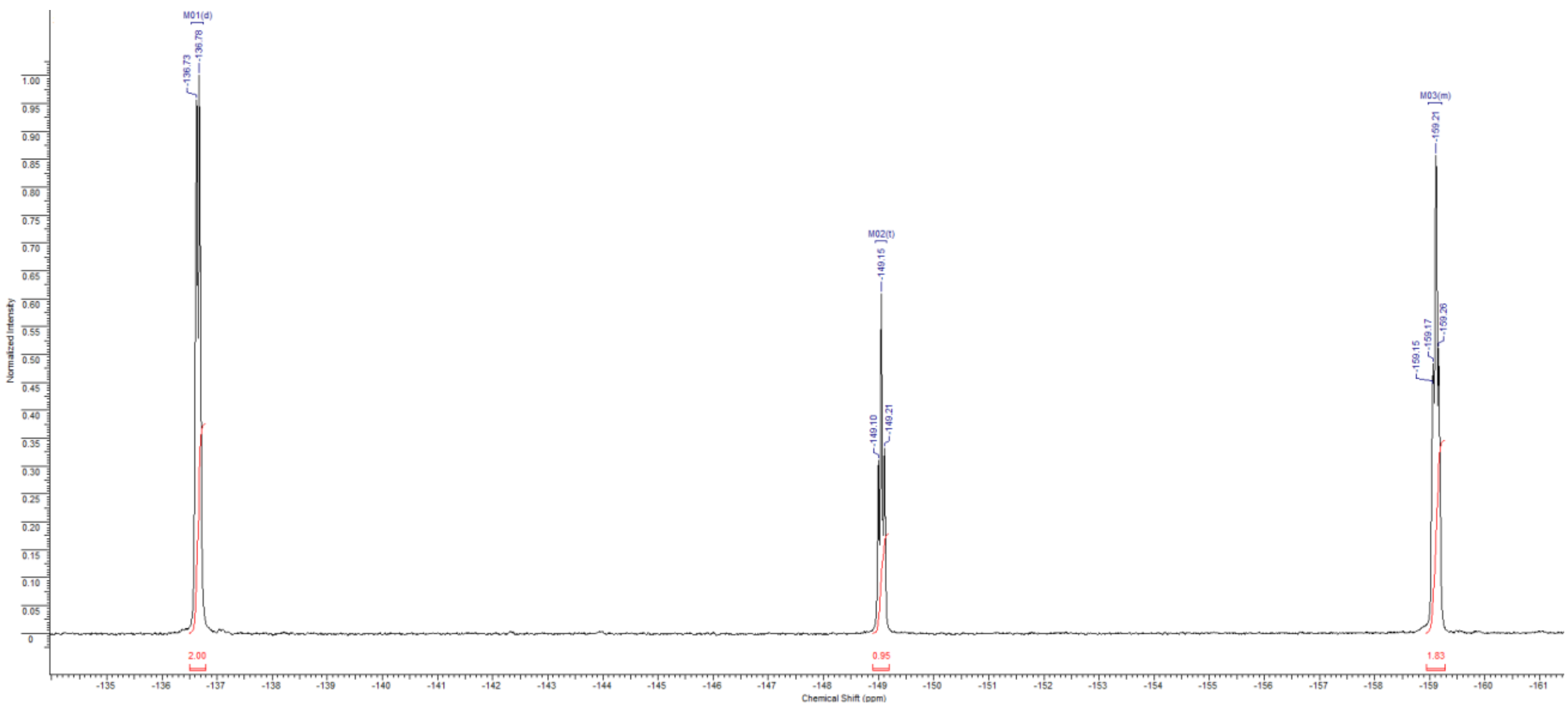

Figure S21: ^{19}F NMR (376 MHz, $CDCl_3$) spectrum of compound **14**.

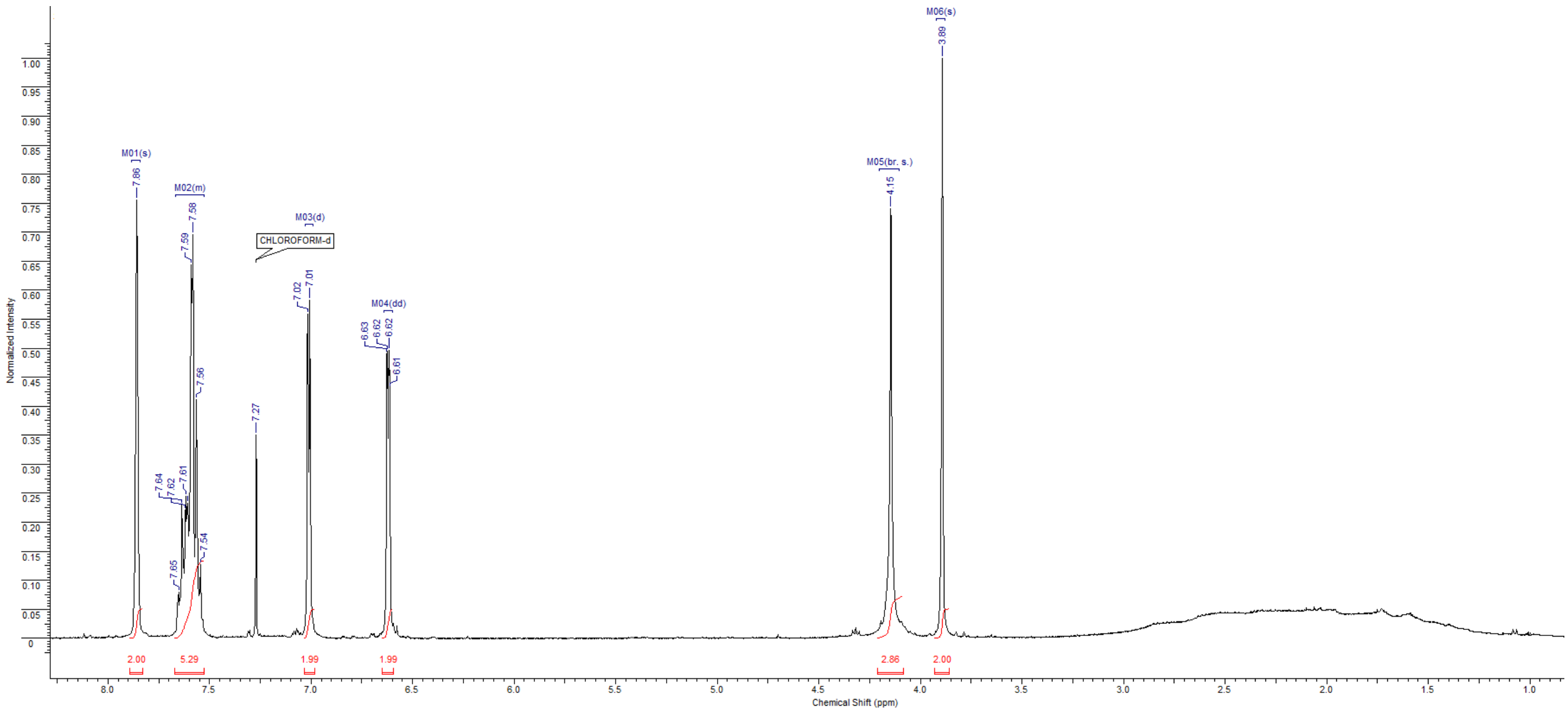

Figure S22: ^{1}H NMR (400 MHz, $CDCl_3$) spectrum of compound **15**.

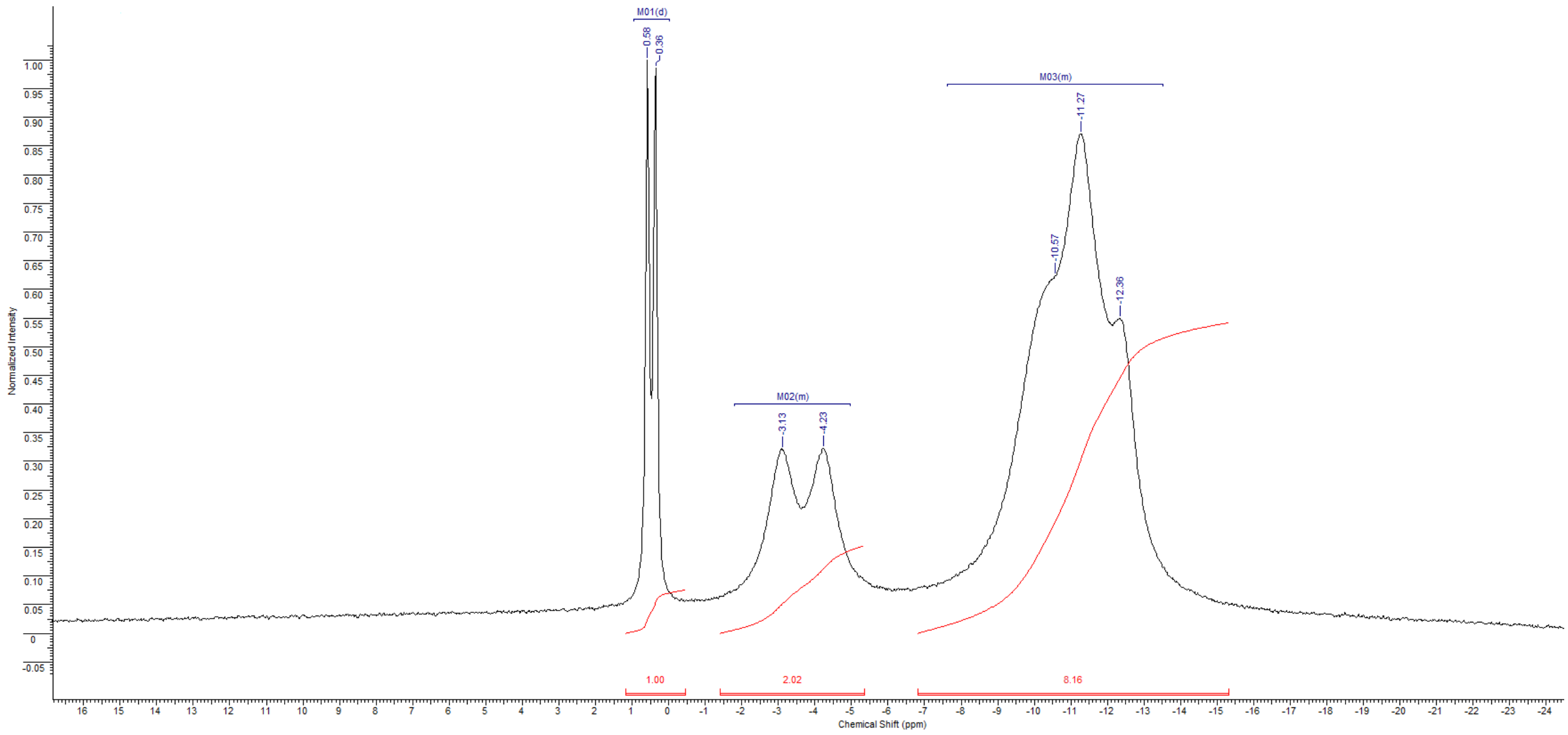

Figure S23: ^{11}B NMR (128 MHz, $CDCl_3$) spectrum of compound **15**.

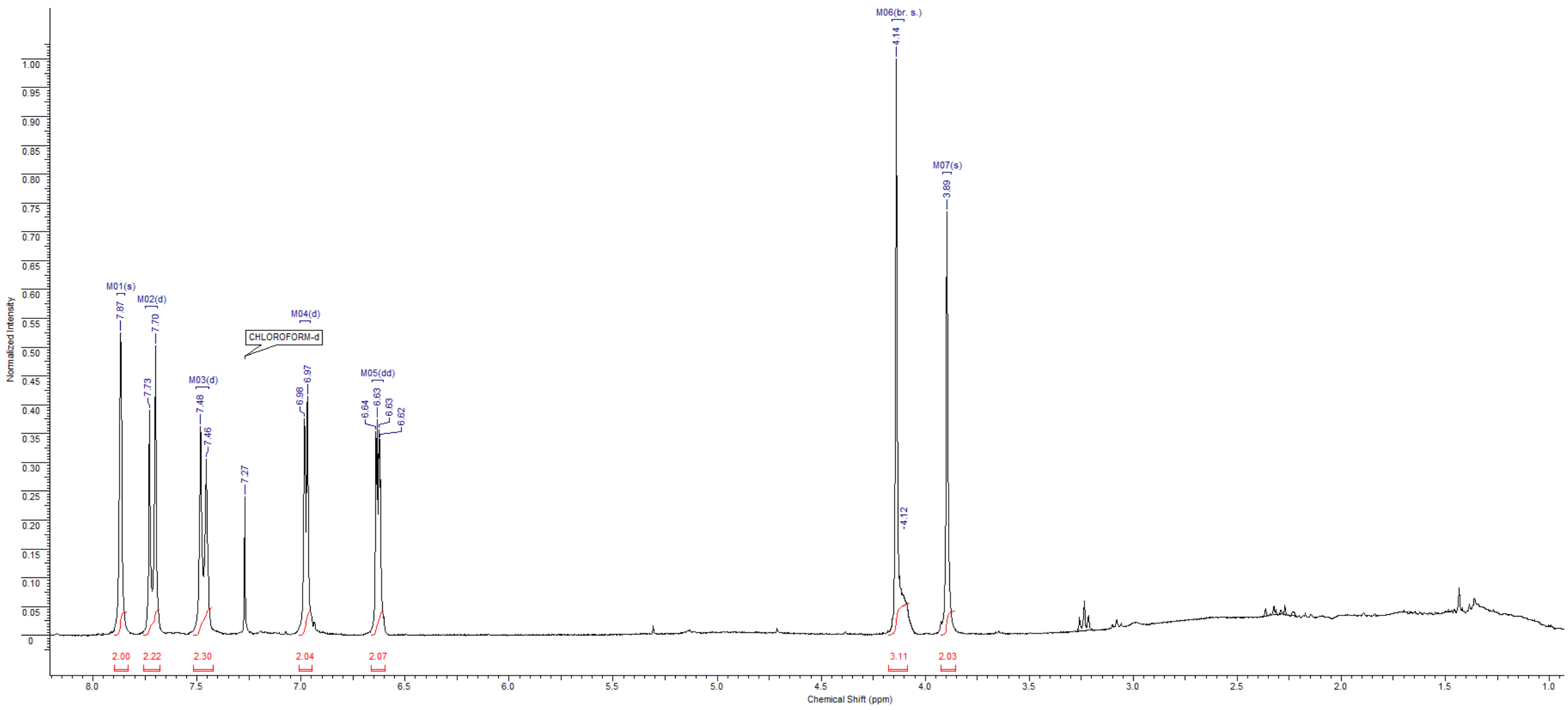

Figure S24: ^{1}H NMR (400 MHz, $CDCl_3$) spectrum of compound **16**.

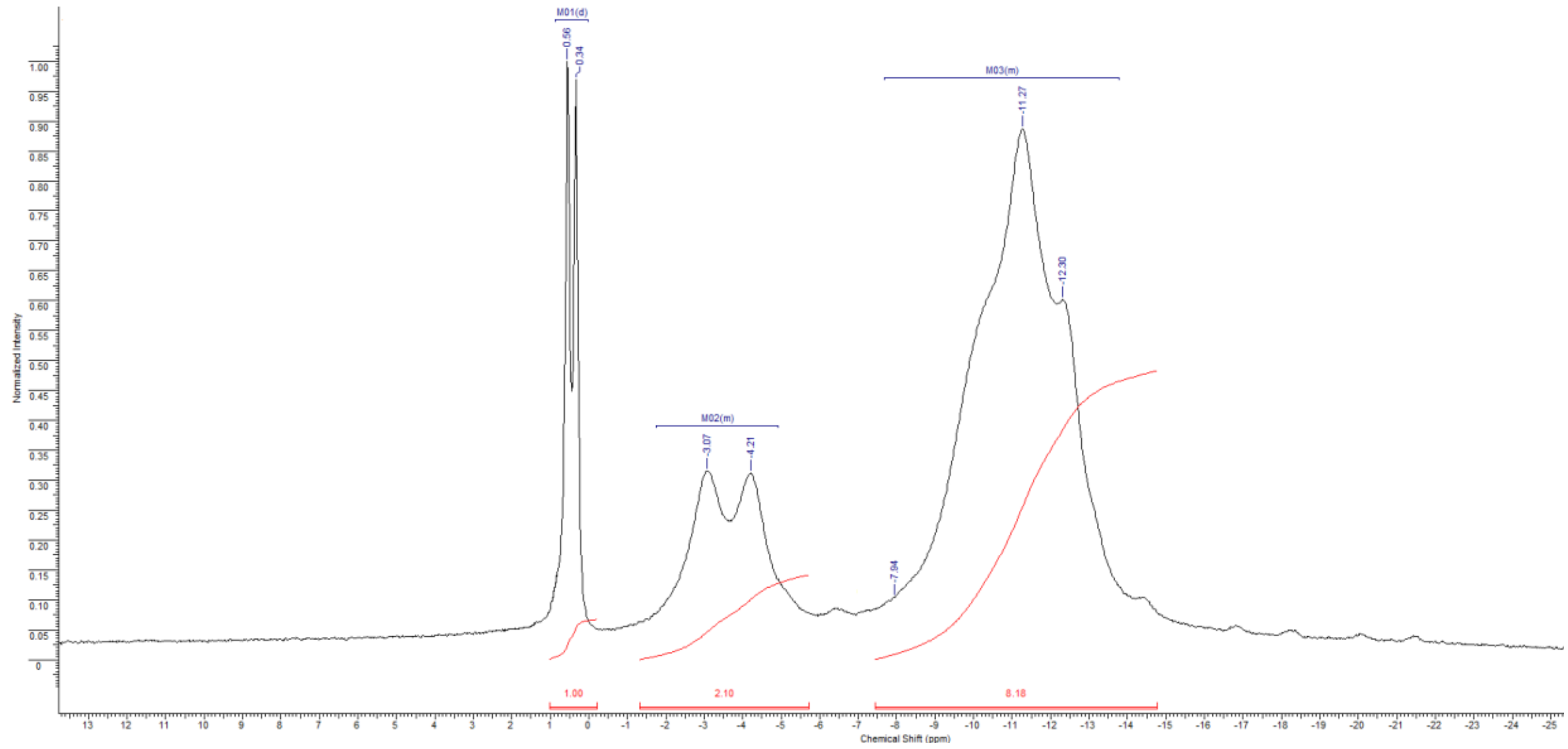

Figure S25: ^{11}B NMR (128 MHz, $CDCl_3$) spectrum of compound **16**.

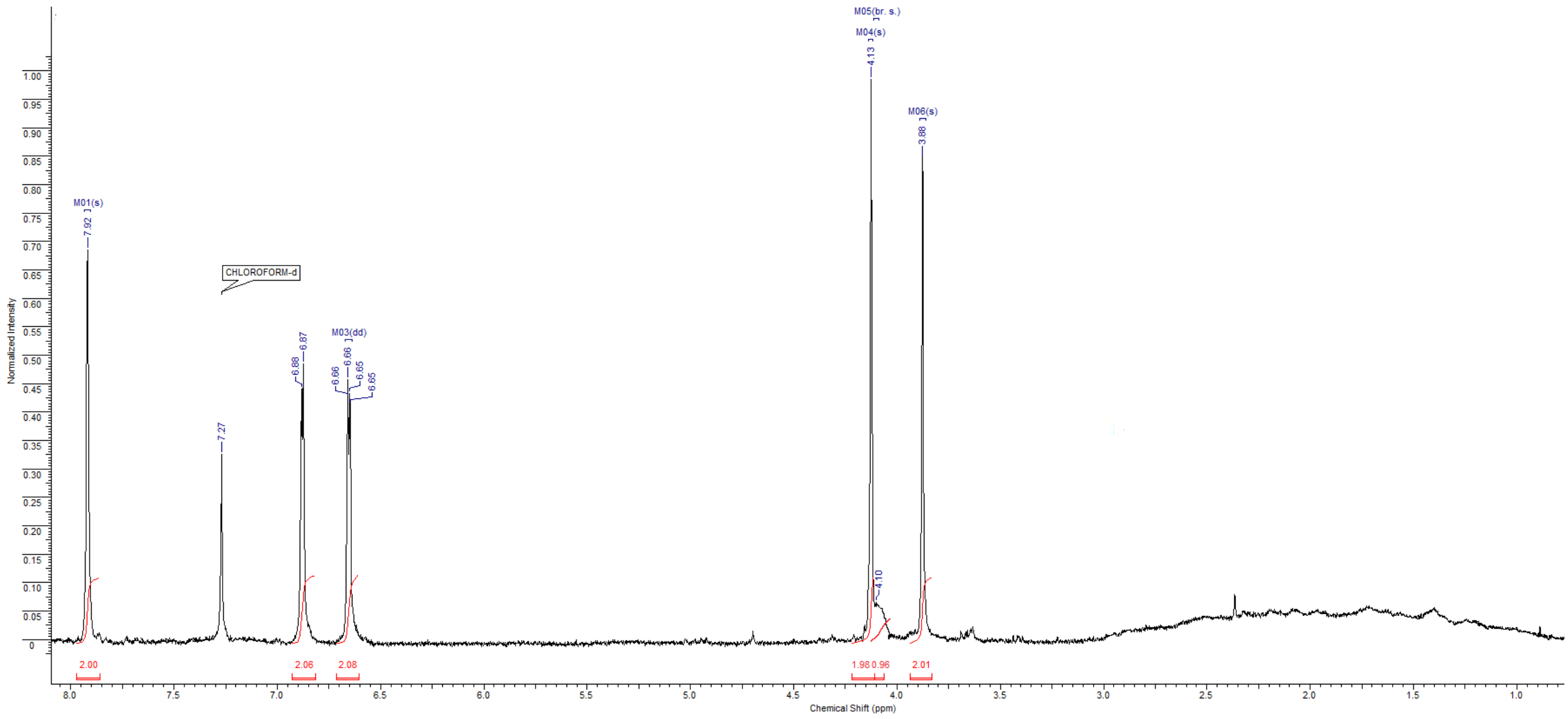

Figure S26: ^{1}H NMR (400 MHz, $CDCl_3$) spectrum of compound **17**.

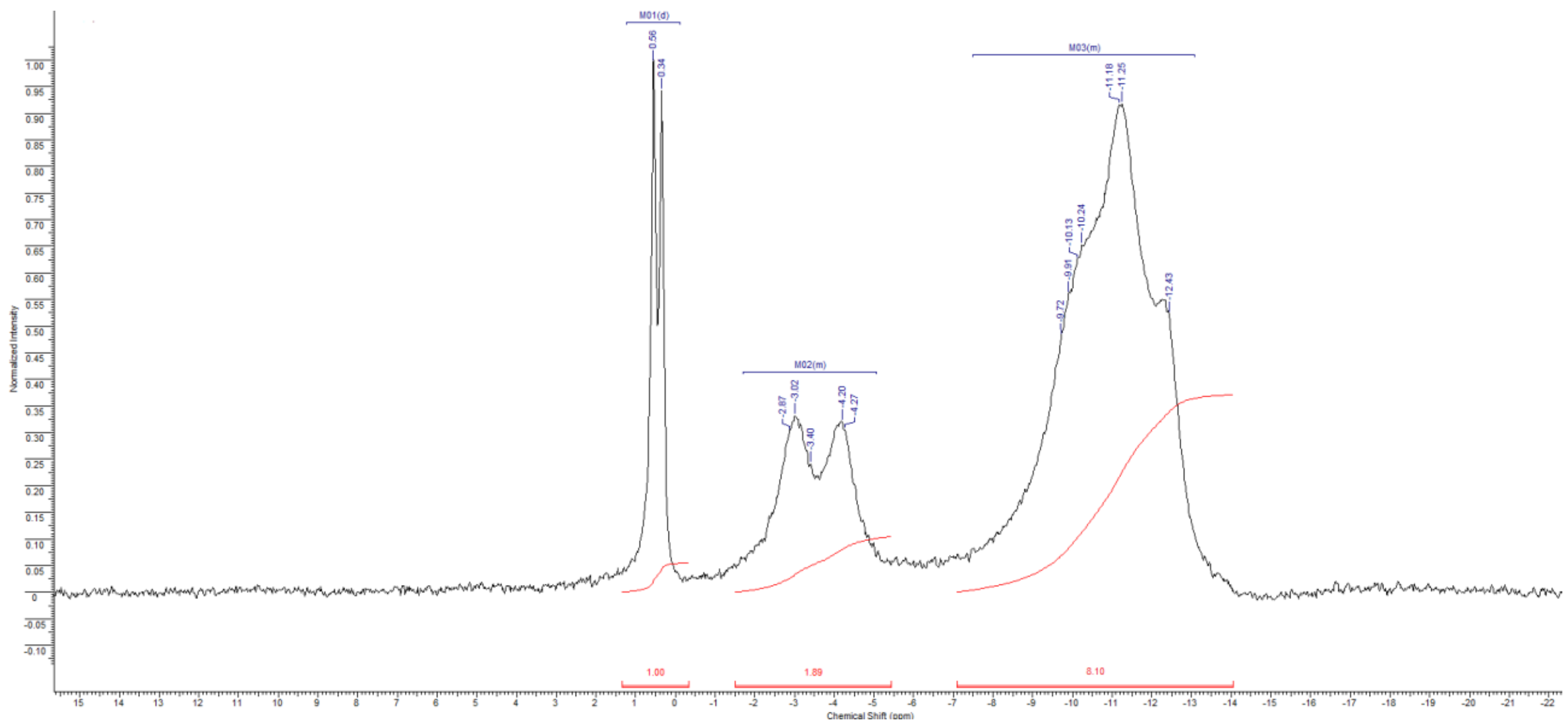

Figure S27: ^{11}B NMR (128 MHz, $CDCl_3$) spectrum of compound **17**.

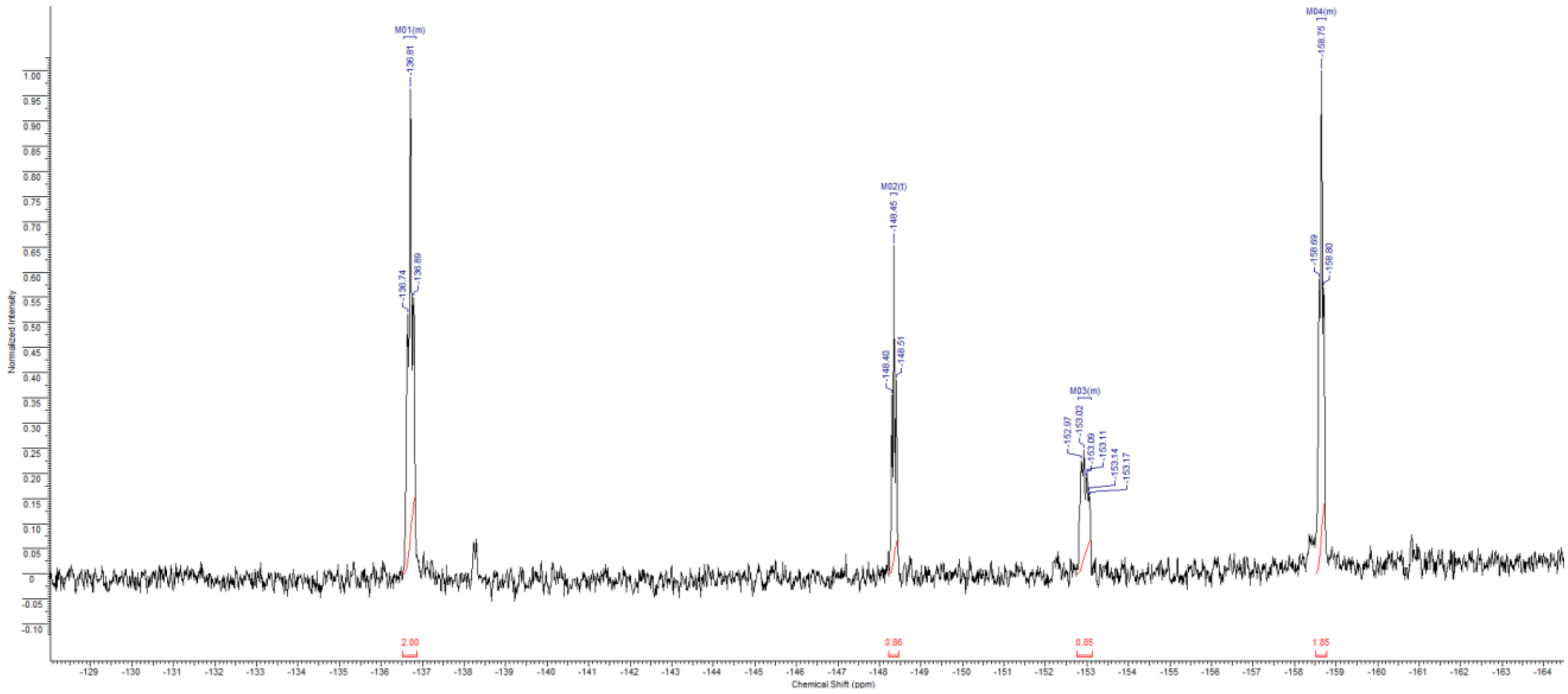

Figure S28: ^{19}F NMR (376 MHz, $CDCl_3$) spectrum of compound **17**.

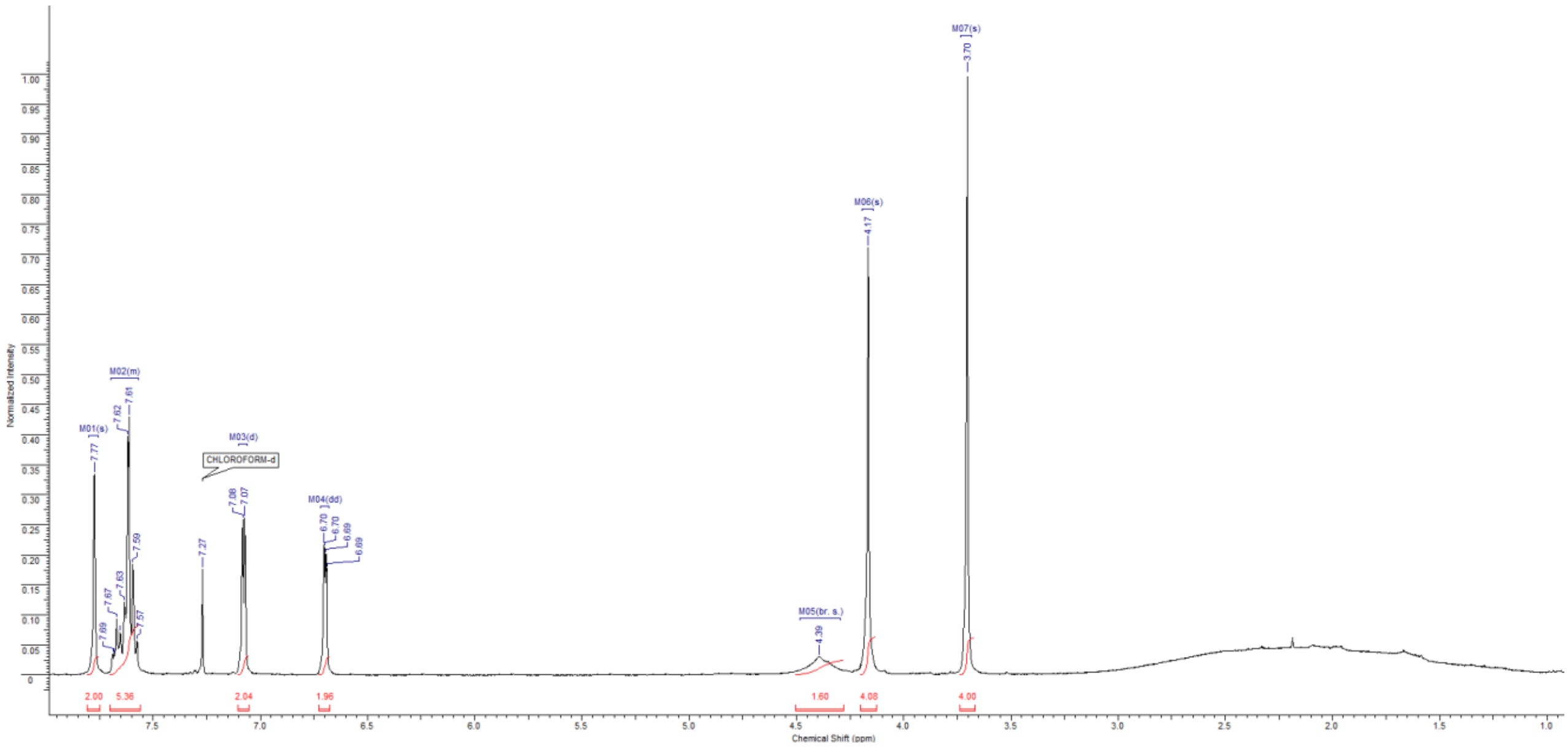

Figure S29: ^{1}H NMR (400 MHz, $CDCl_3$) spectrum of compound **18**.

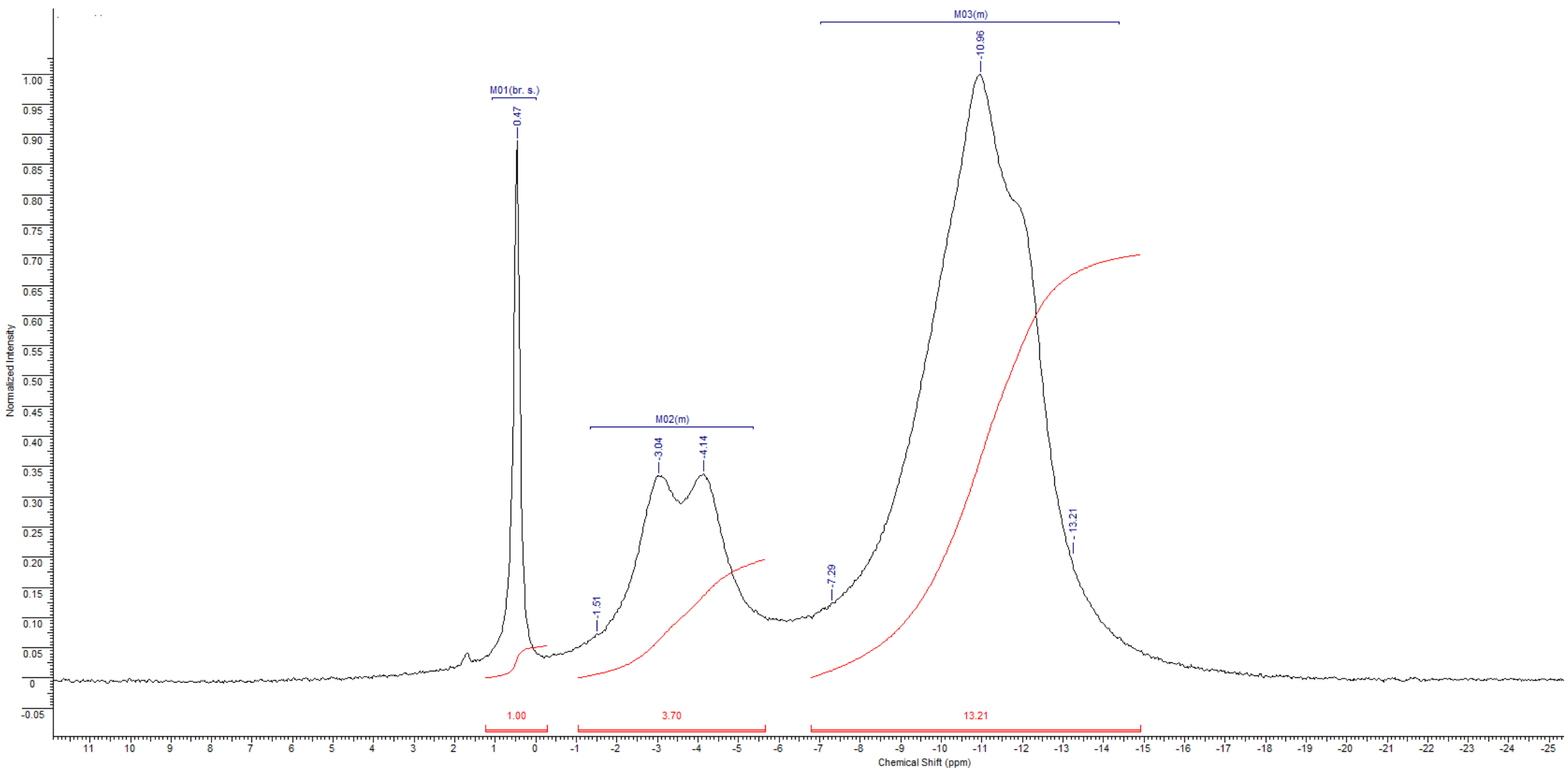

Figure S30: ^{11}B NMR (128 MHz, $CDCl_3$) spectrum of compound **18**

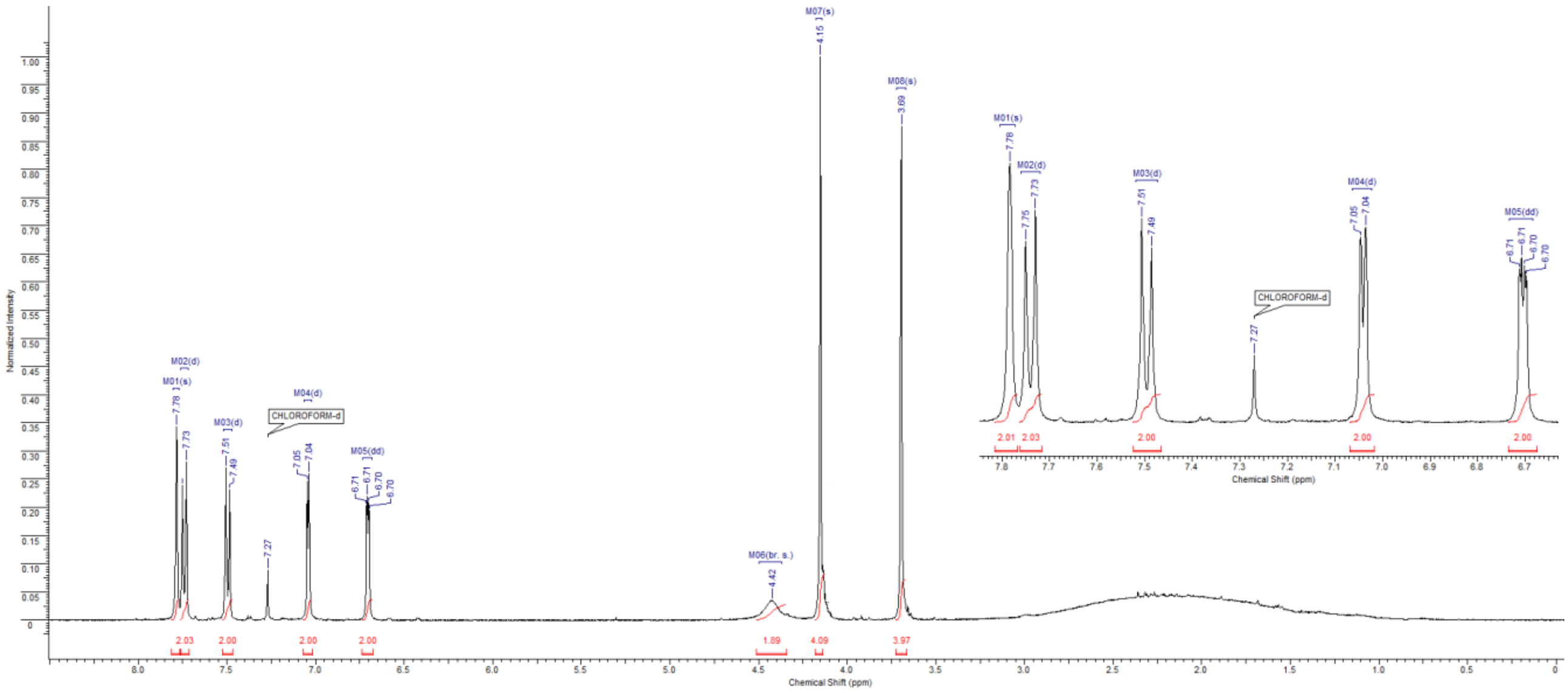

Figure S31: ^{1}H NMR (400 MHz, $CDCl_3$) spectrum of compound **19**.

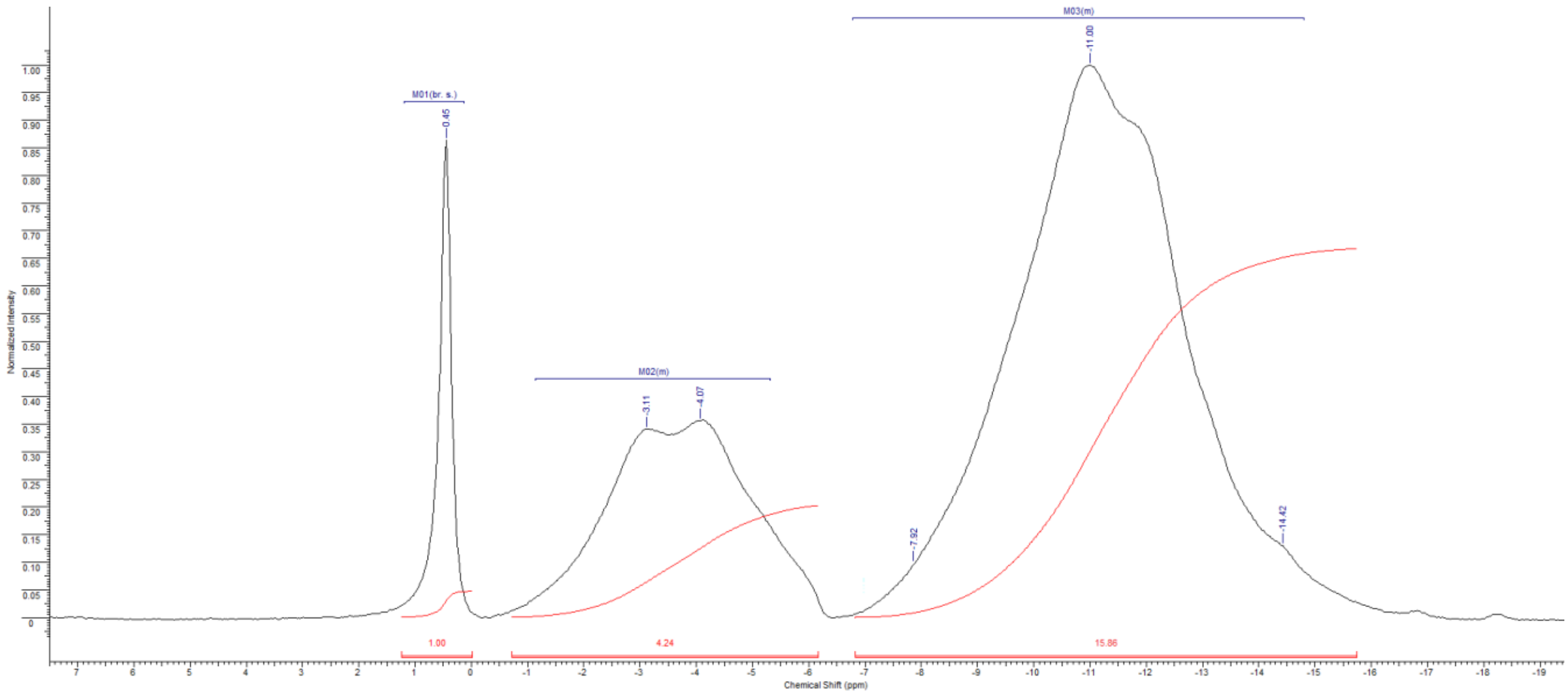

Figure S32: ^{11}B NMR (128 MHz, $CDCl_3$) spectrum of compound **19.**

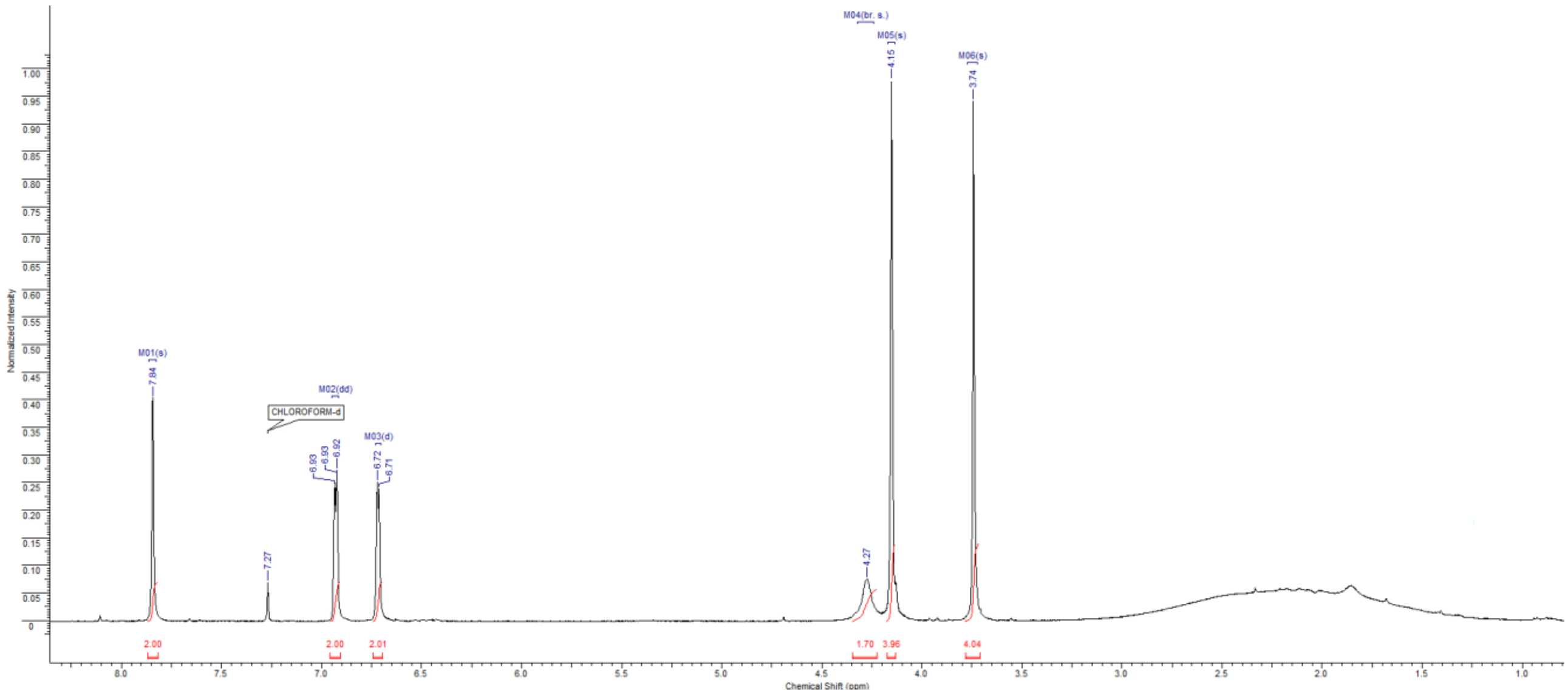

Figure S33: ^{1}H NMR (400 MHz, $CDCl_3$) spectrum of compound **20**.

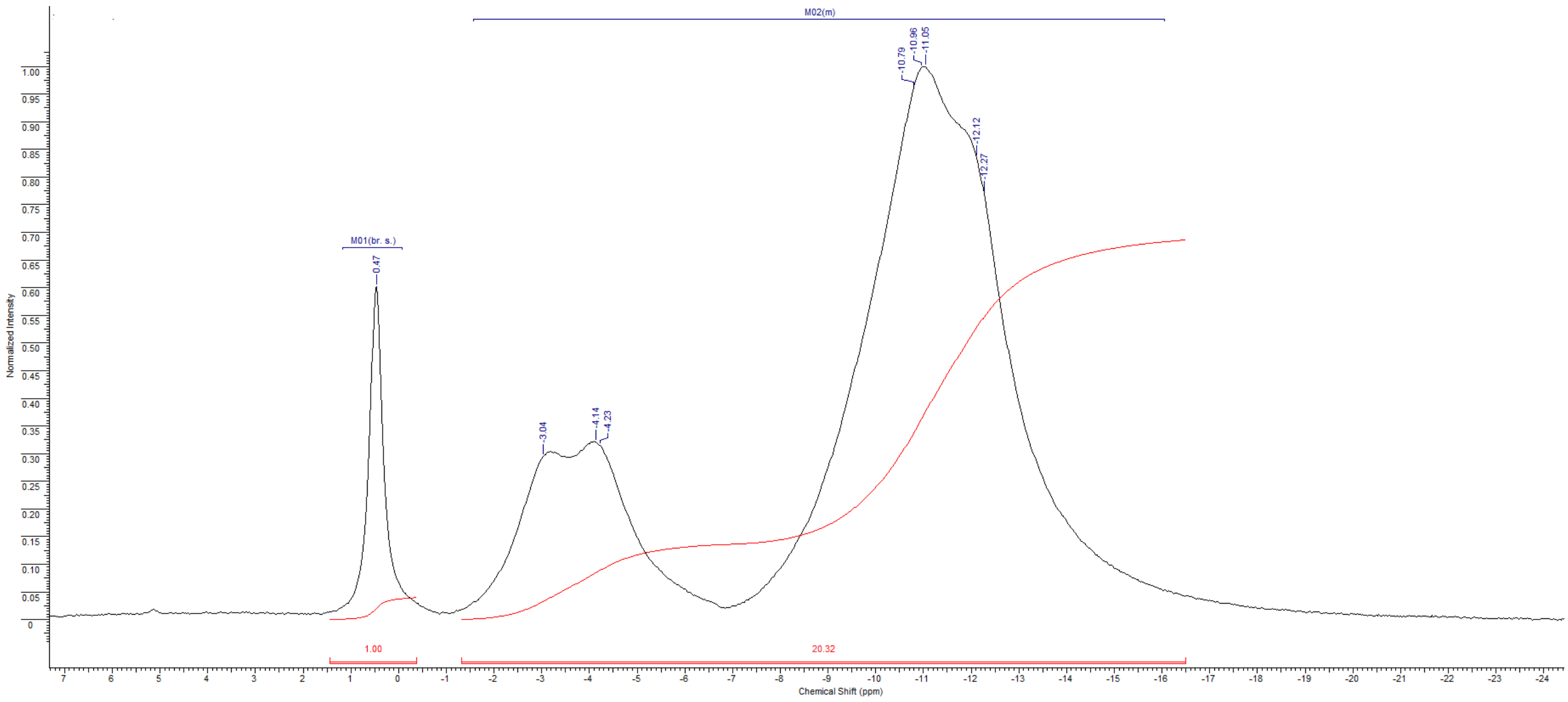

Figure S34: ^{11}B NMR (128 MHz, $CDCl_3$) spectrum of compound **20.**

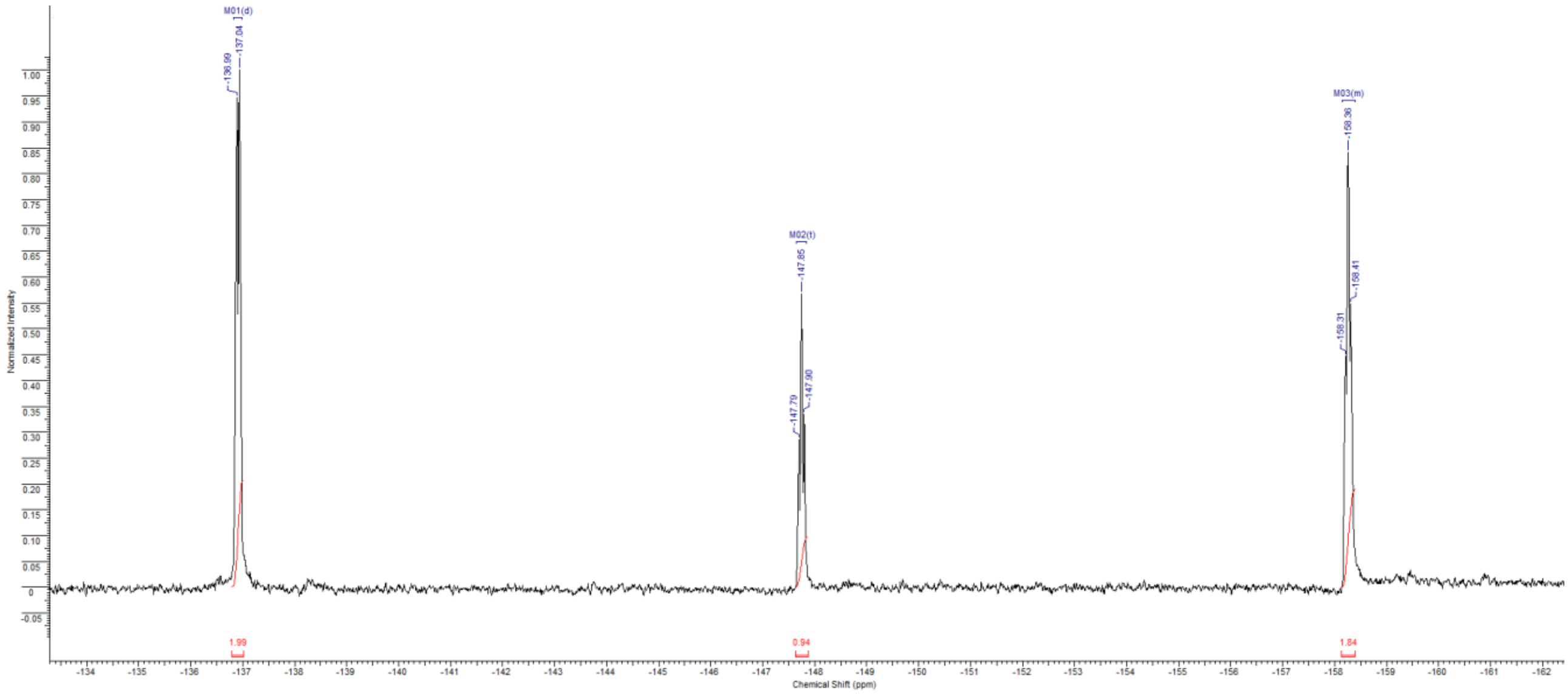

Figure S35: ^{19}F NMR (376 MHz, $CDCl_3$) spectrum of compound **20**.

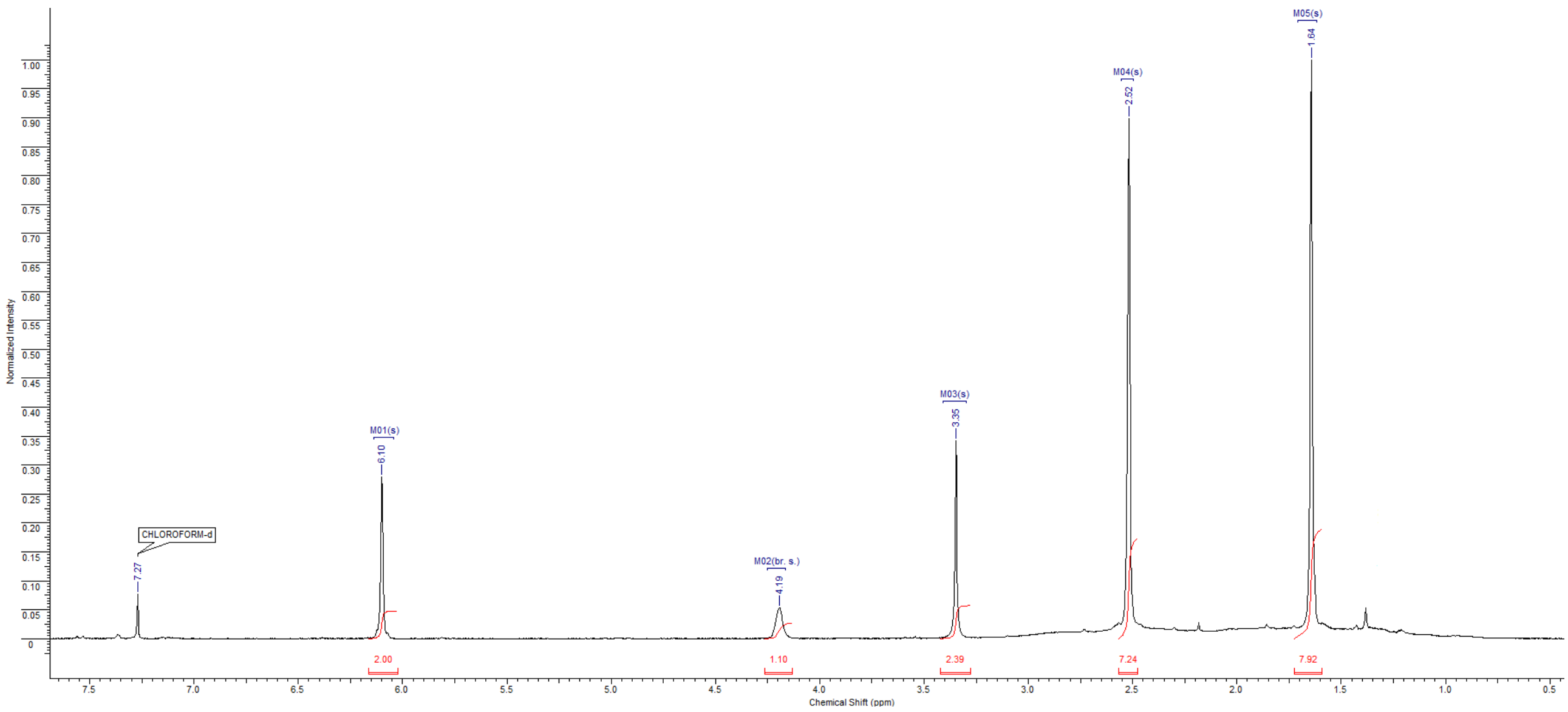

Figure S36: ^{1}H NMR (400 MHz, $CDCl_3$) spectrum of compound **22**.

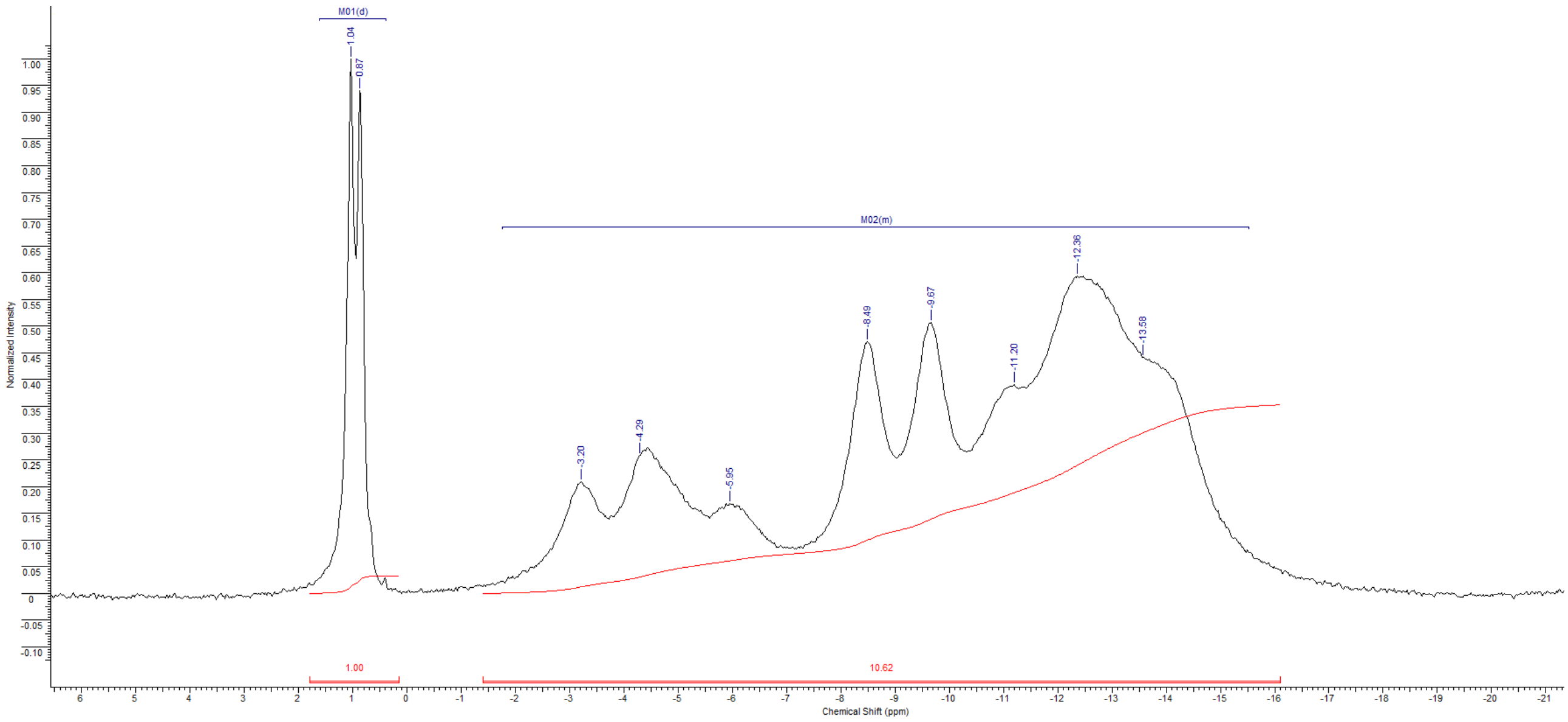

Figure S37: ^{11}B NMR (128 MHz, $CDCl_3$) spectrum of compound **22.**

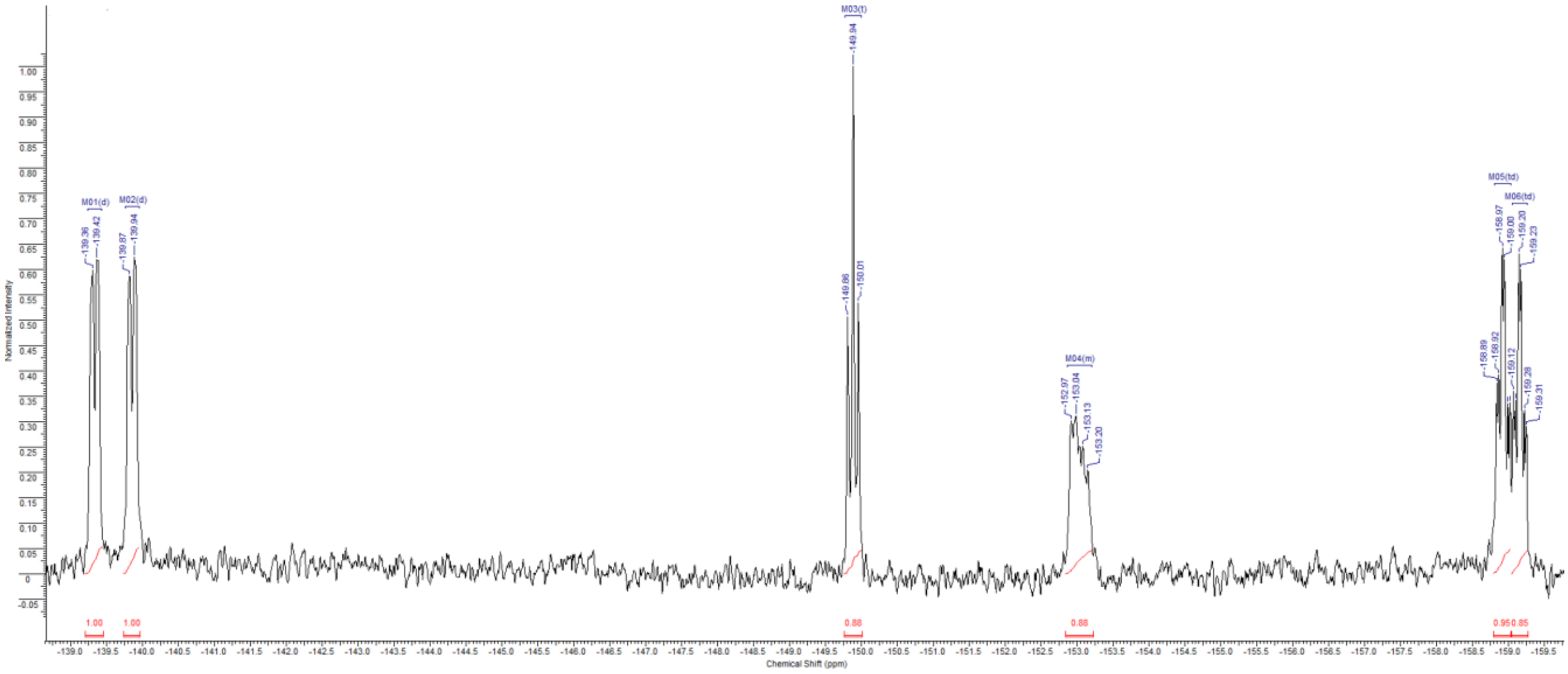

Figure S38: ^{19}F NMR (376 MHz, $CDCl_3$) spectrum of compound **22**.

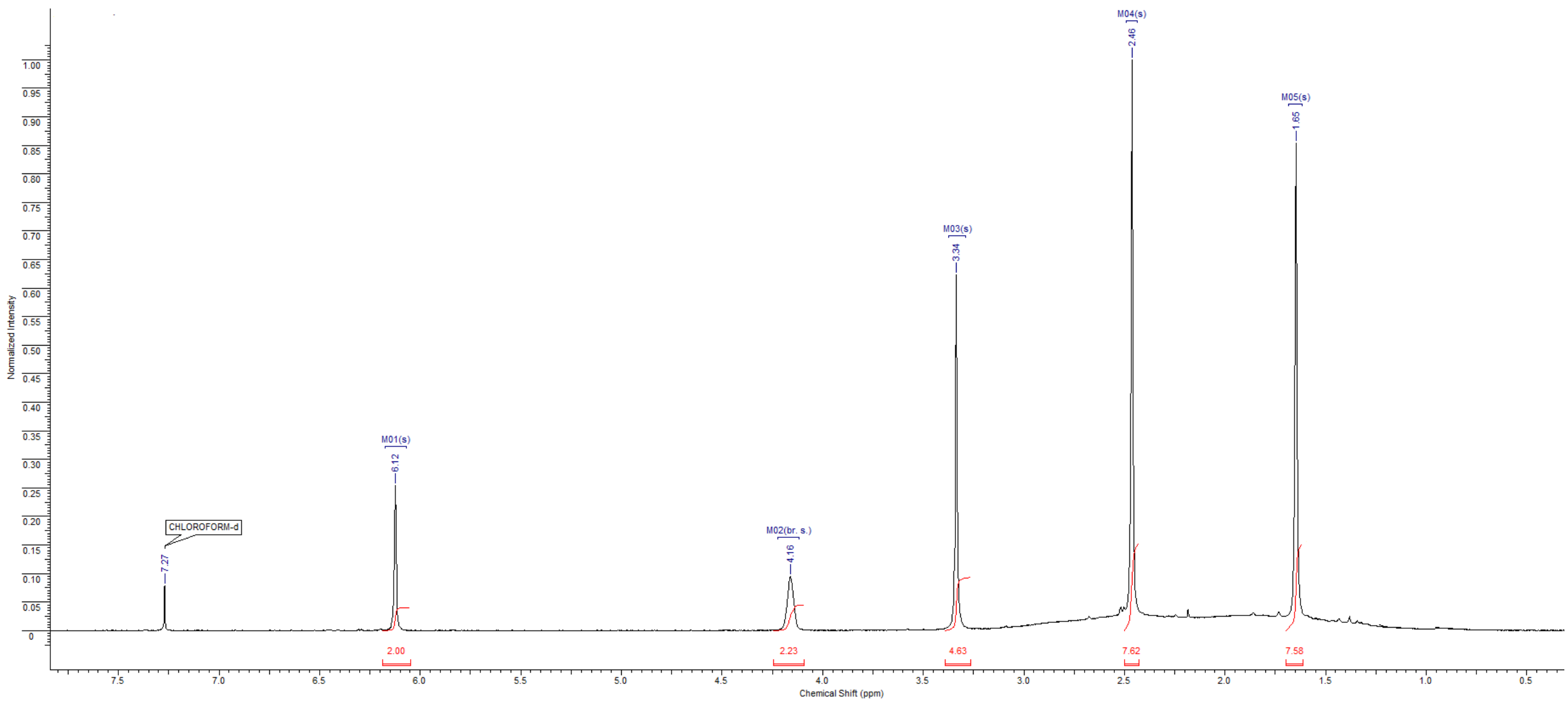

Figure S39: ^{1}H NMR (400 MHz, $CDCl_3$) spectrum of compound **23**.

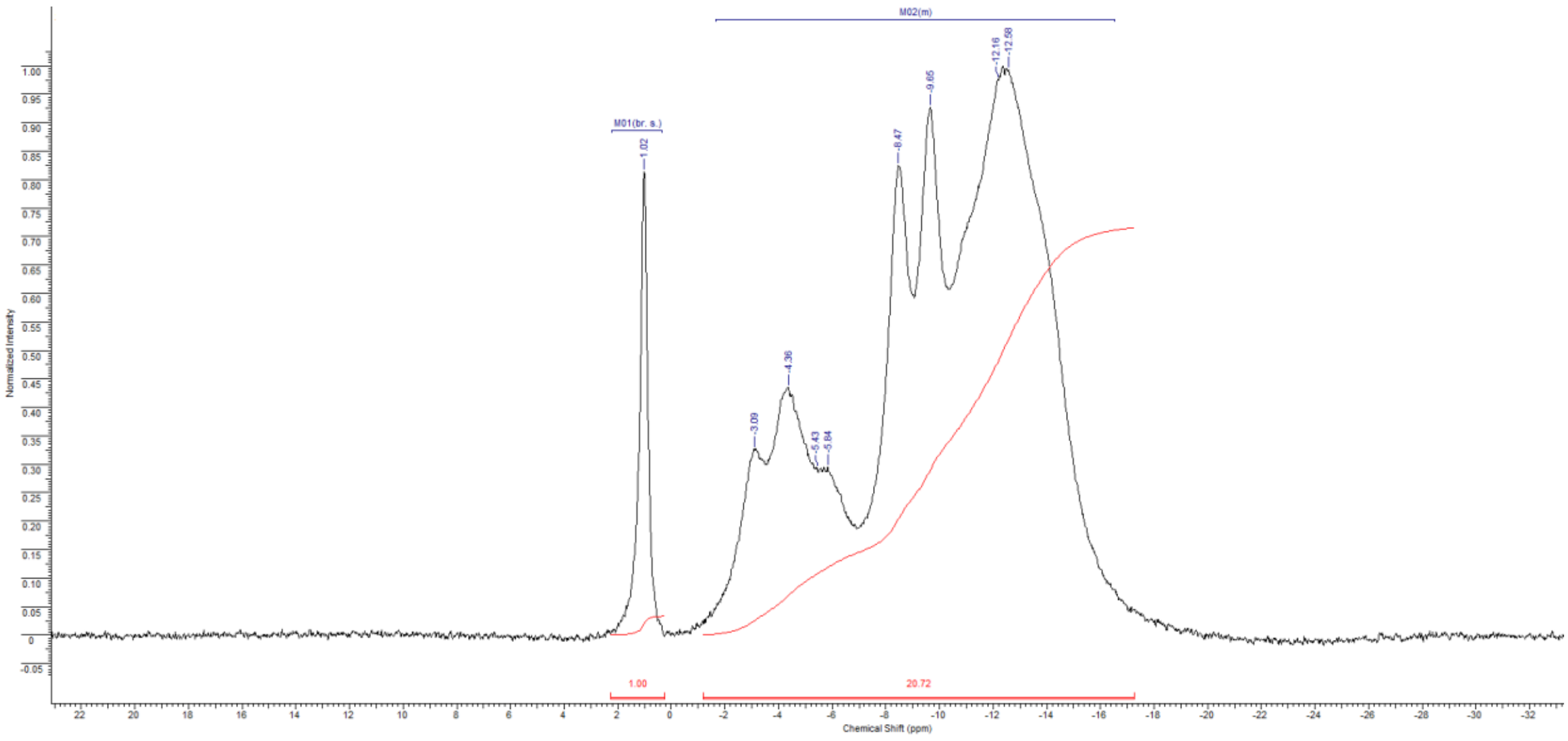

Figure S40: ^{11}B NMR (128 MHz, $CDCl_3$) spectrum of compound **23**.

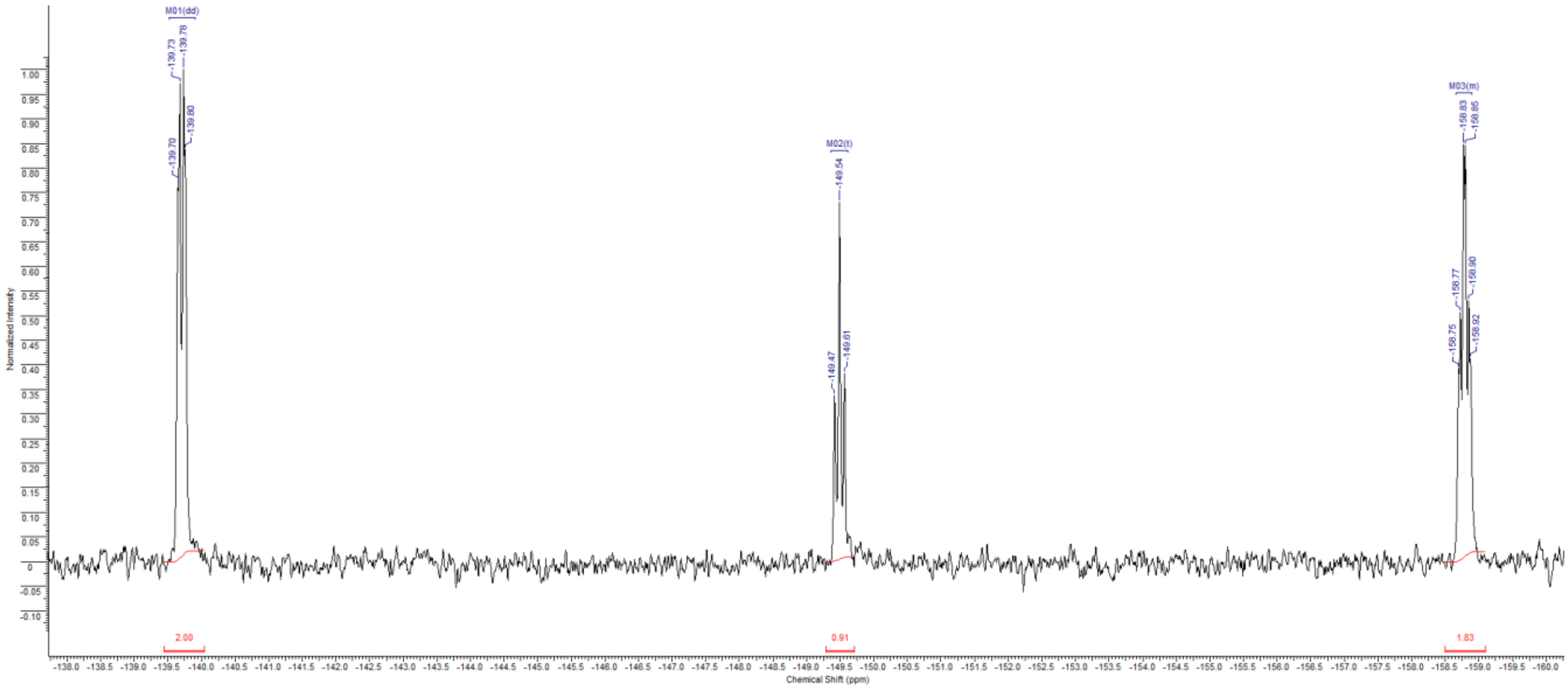

Figure S41: ^{19}F NMR (376 MHz, $CDCl_3$) spectrum of compound **23**.

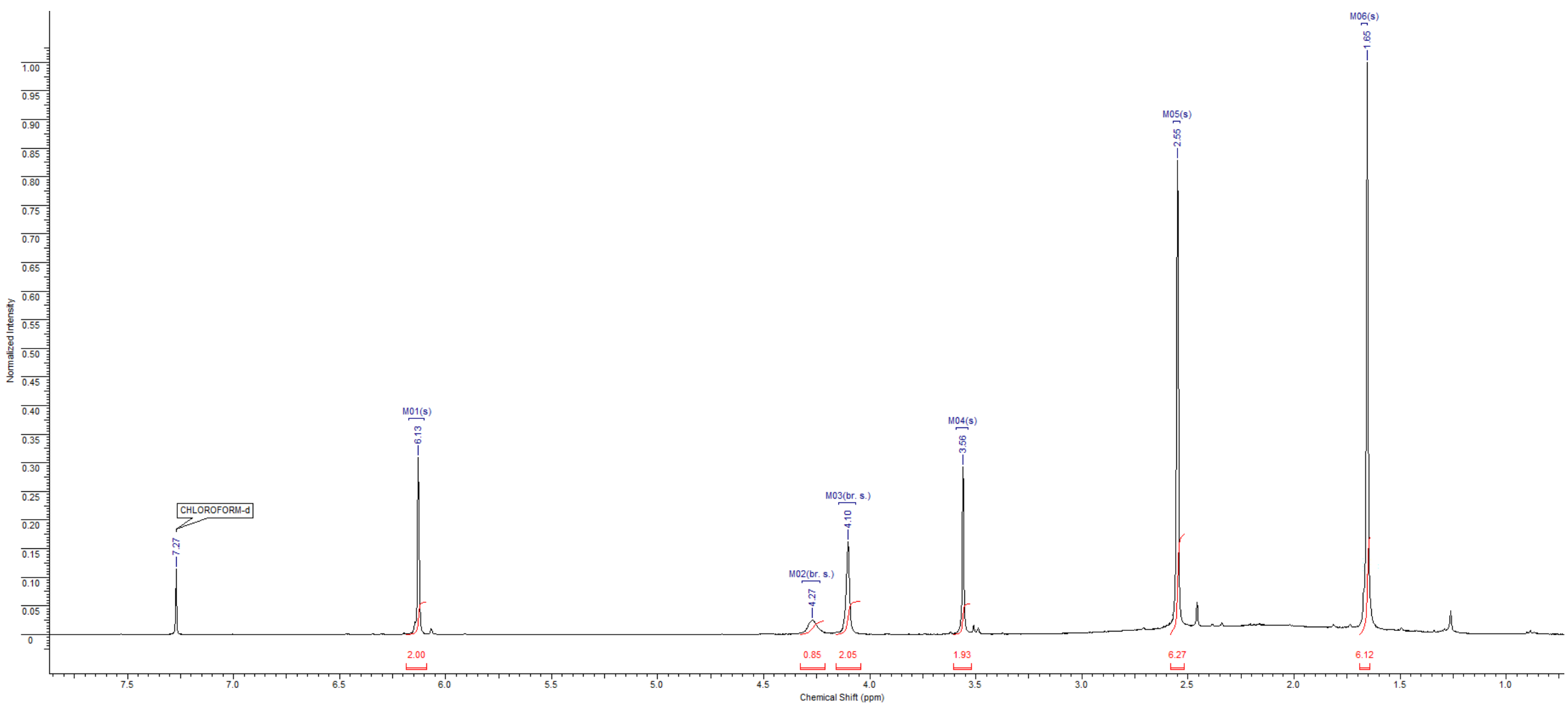

Figure S42: ^{1}H NMR (400 MHz, $CDCl_3$) spectrum of compound **24**.

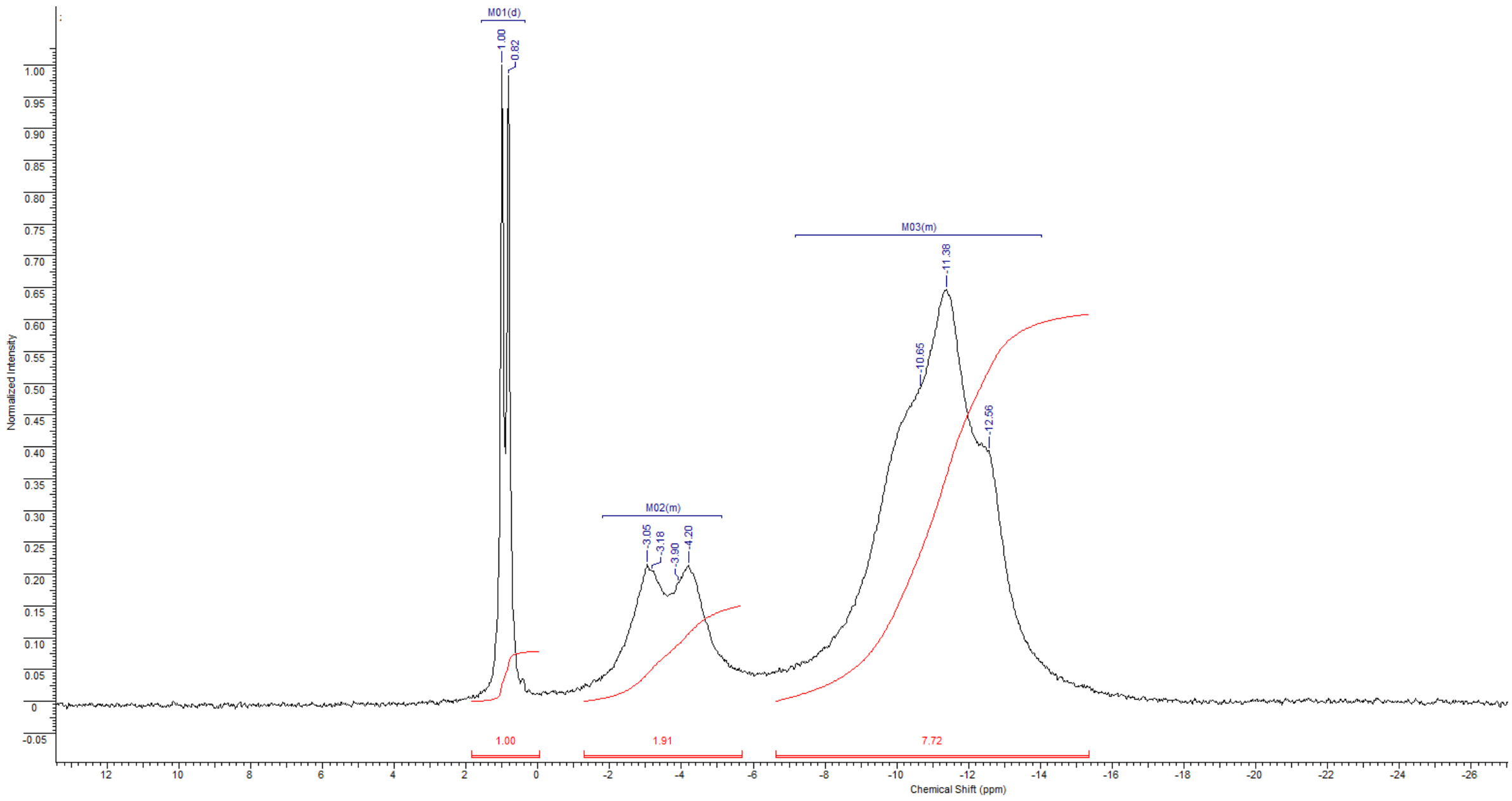

Figure S43: ^{11}B NMR (128 MHz, $CDCl_3$) spectrum of compound **24**.

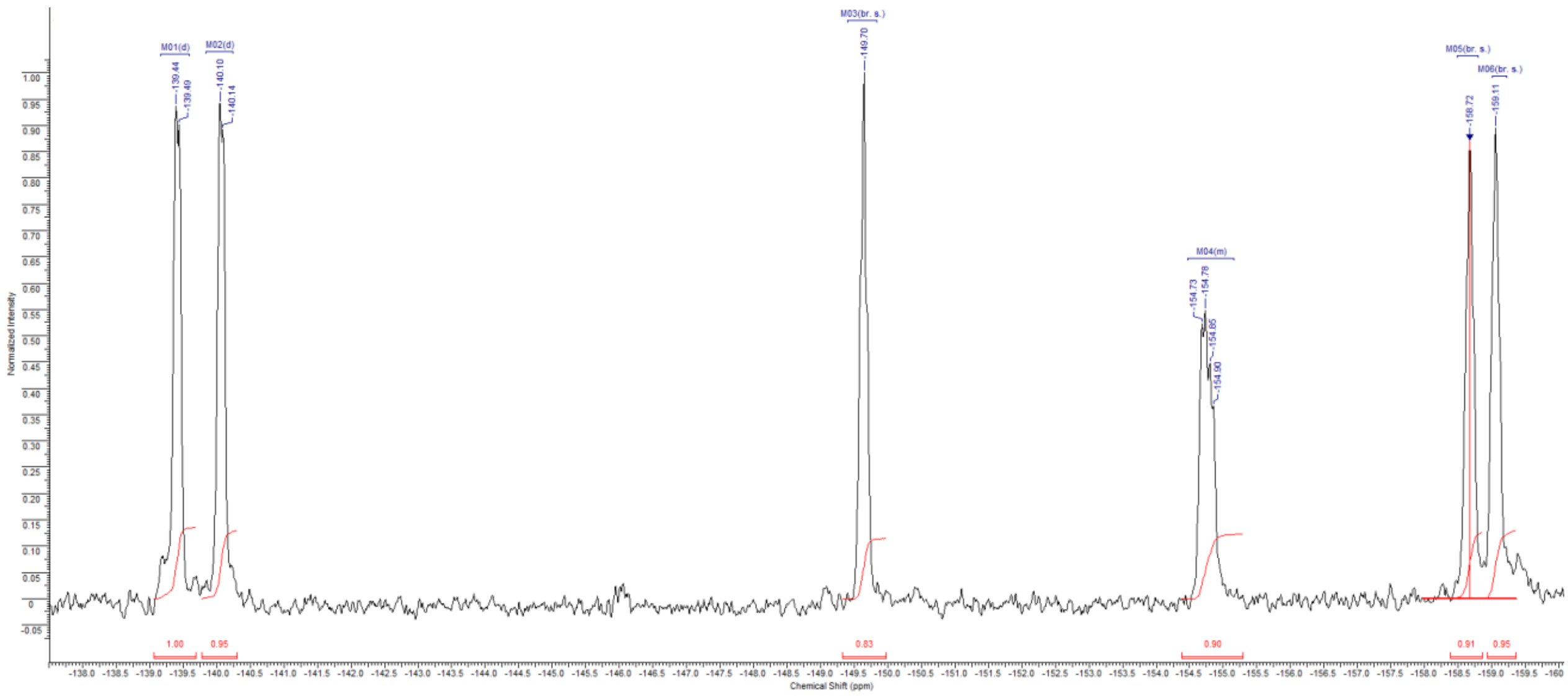

Figure S44: ^{19}F NMR (376 MHz, $CDCl_3$) spectrum of compound **24**.

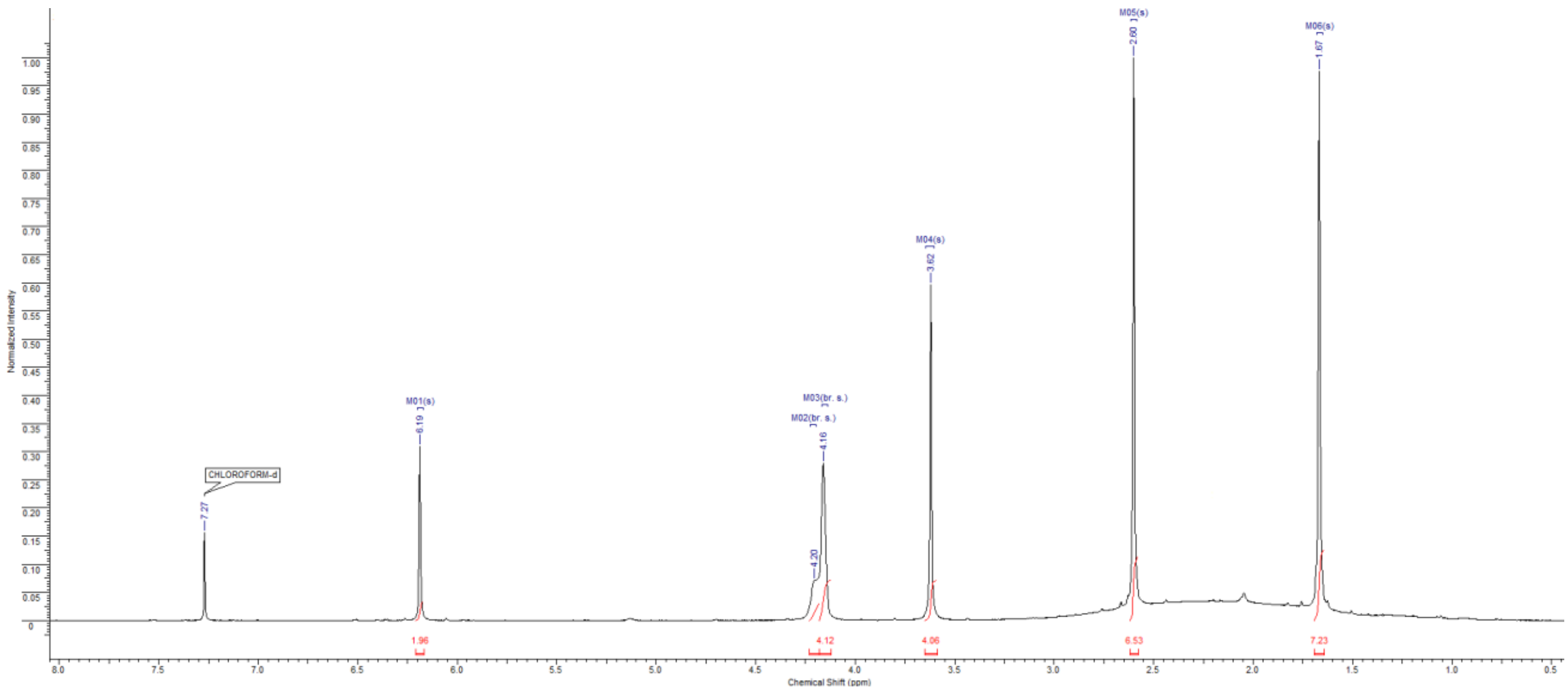

Figure S45: ^{1}H NMR (400 MHz, $CDCl_3$) spectrum of compound **25**.

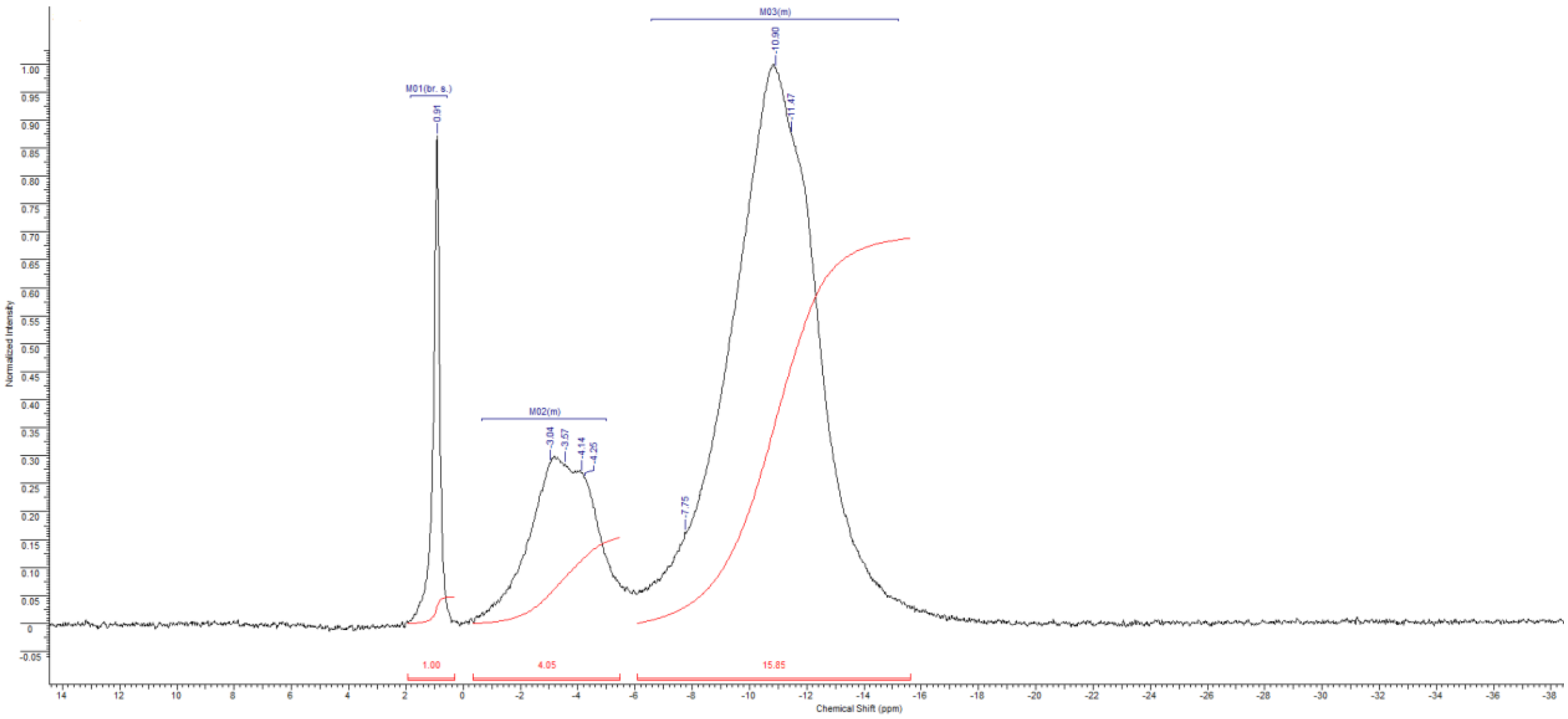

Figure S46: ^{11}B NMR (128 MHz, $CDCl_3$) spectrum of compound **25**.

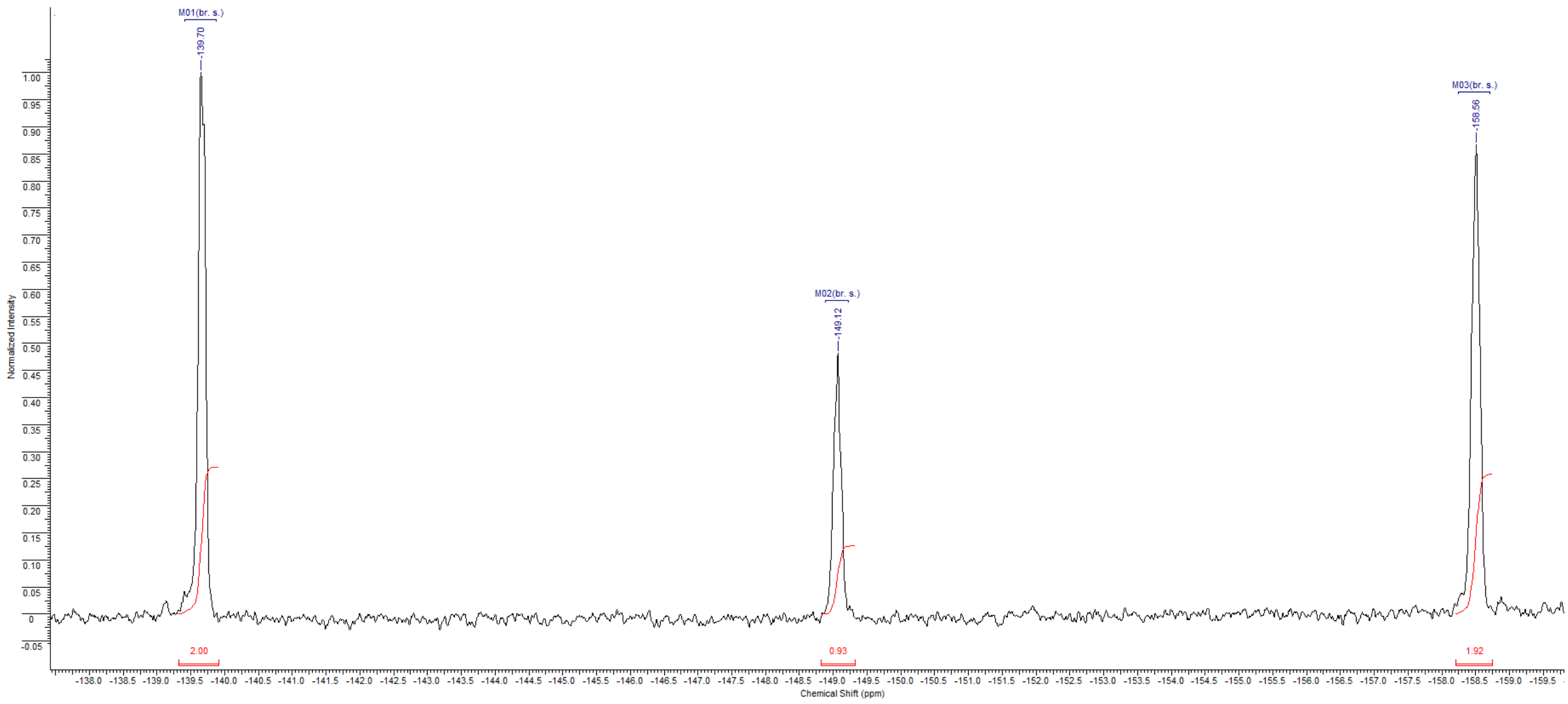

Figure S47: ^{19}F NMR (376 MHz, $CDCl_3$) spectrum of compound **25**.

Mass-spectra of synthesized compounds 6–20 and 22–25

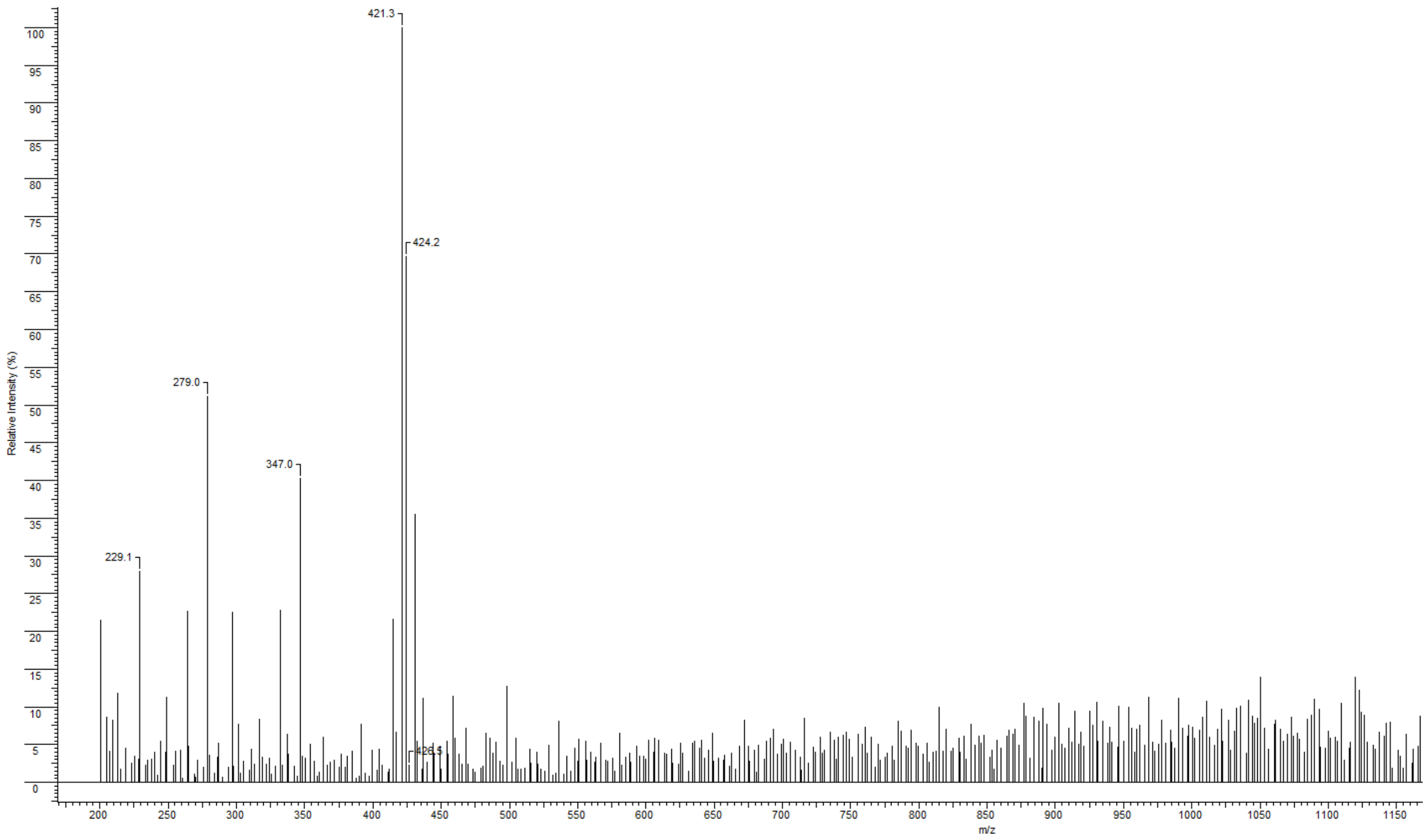

Figure S48: mass-spectrum (ESI) of compound **6**.

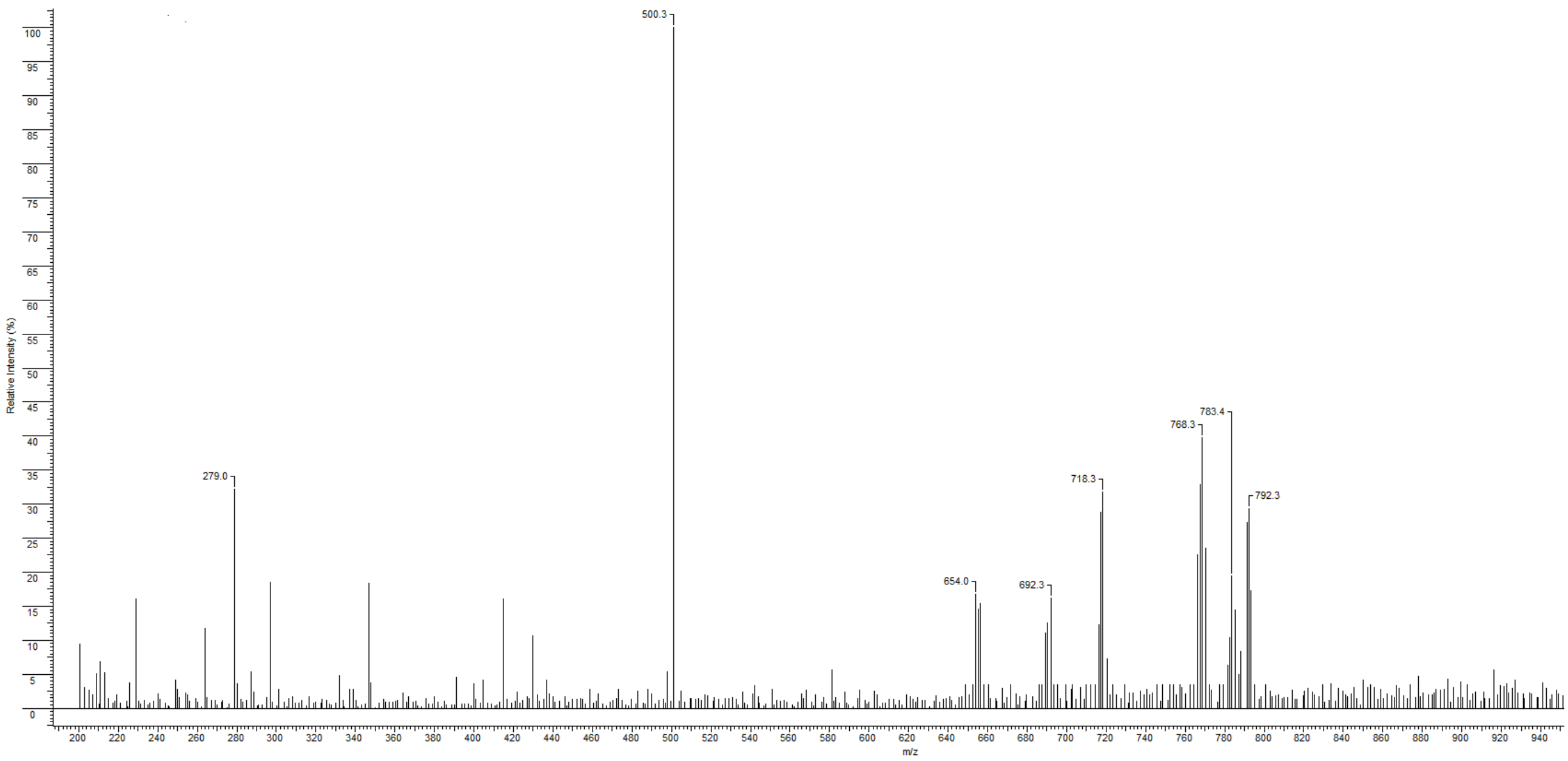

Figure S49: mass-spectrum (ESI) of compound **7**.

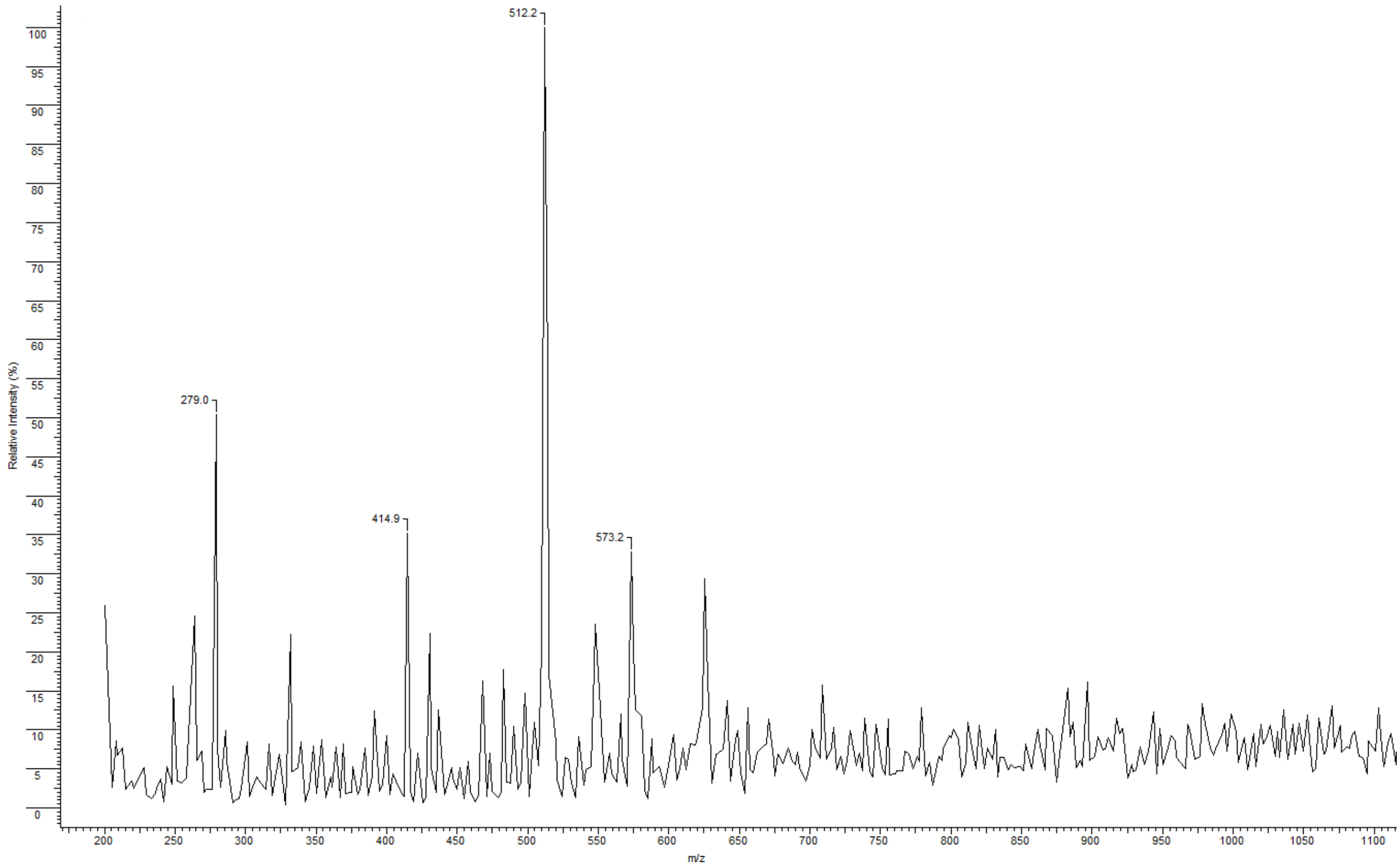

Figure S50: mass-spectrum (ESI) of compound **8**.

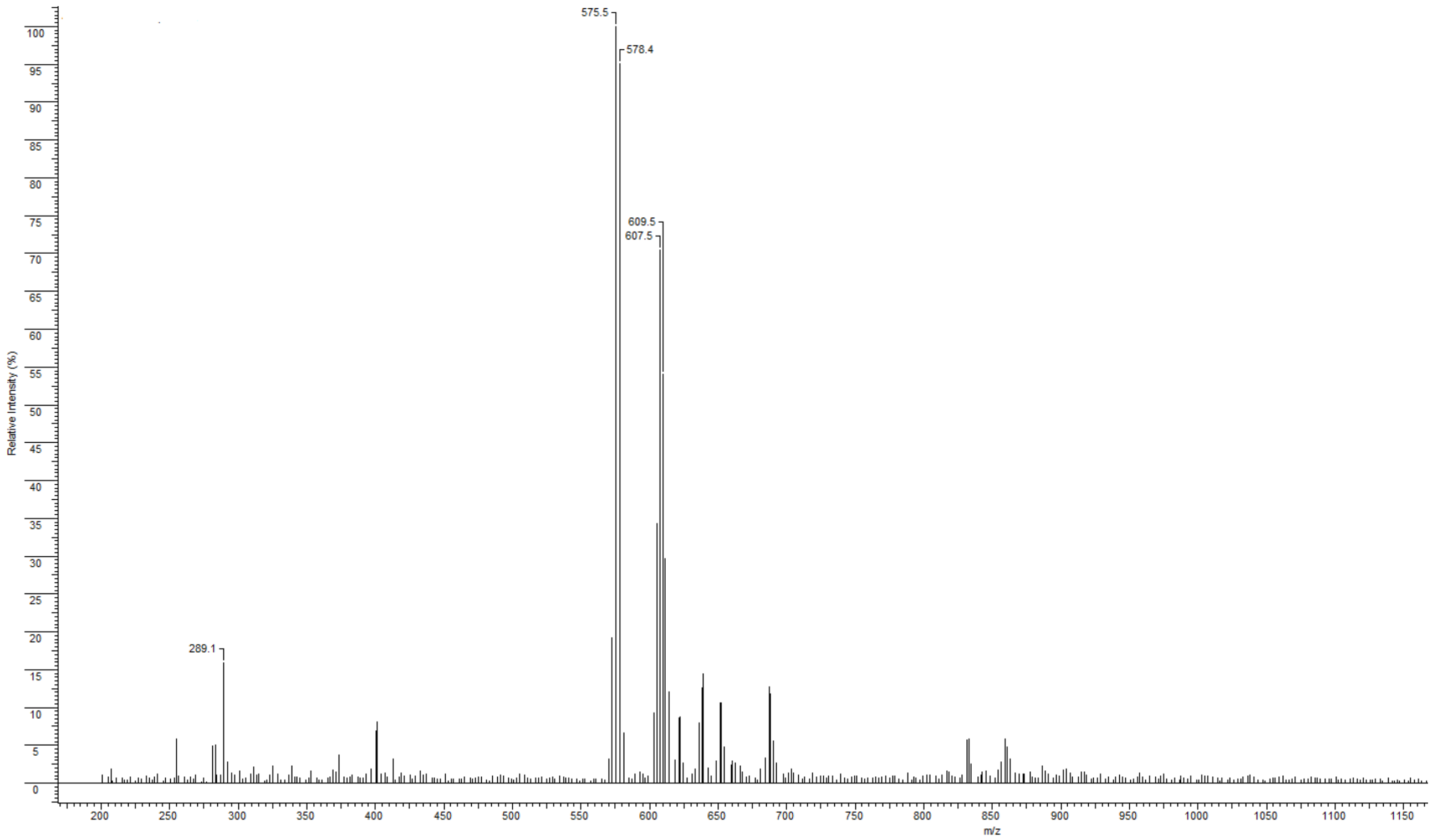

Figure S51: mass-spectrum (ESI) of compound **9**.

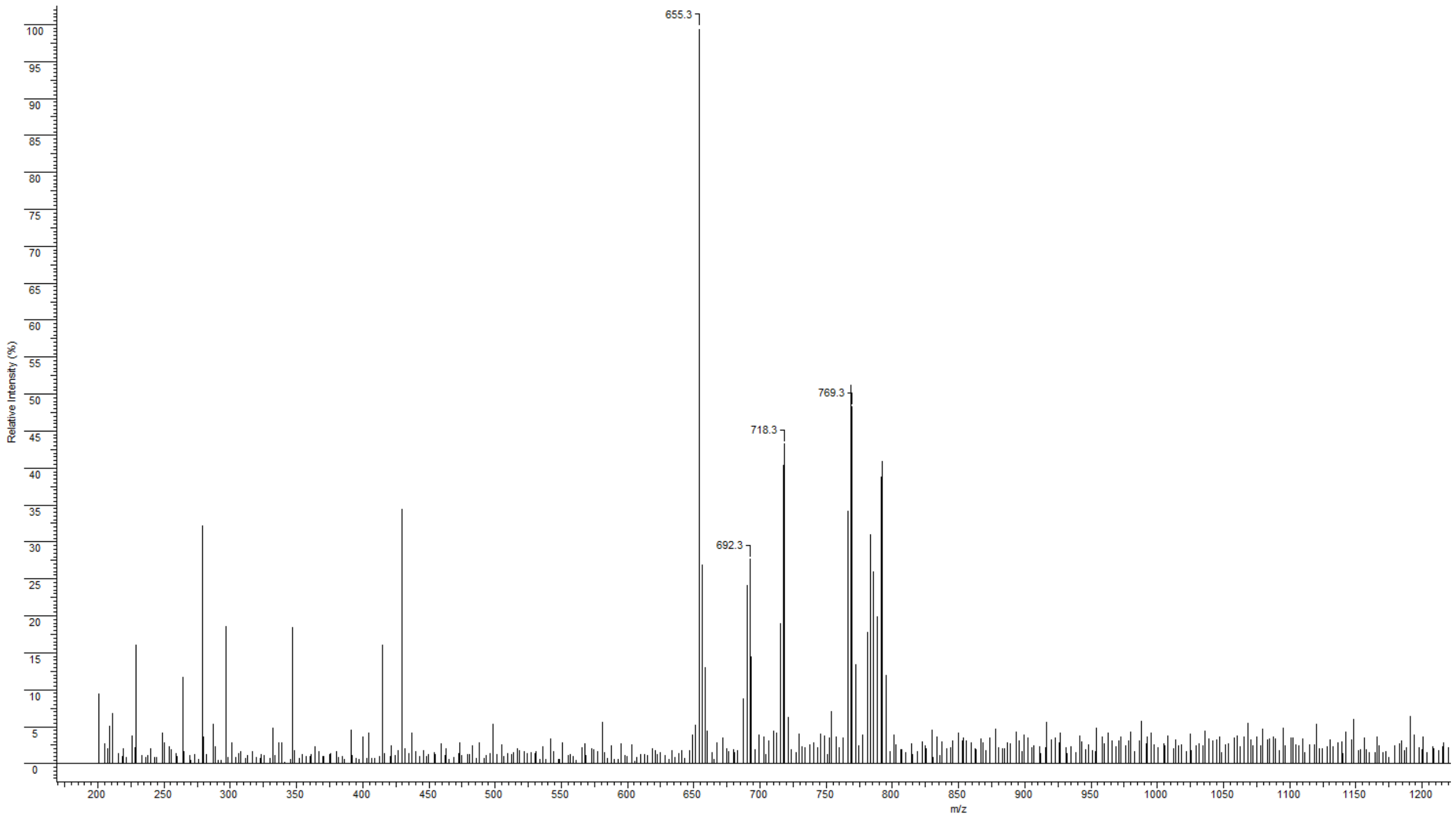

Figure S52: mass-spectrum (ESI) of compound **10**.

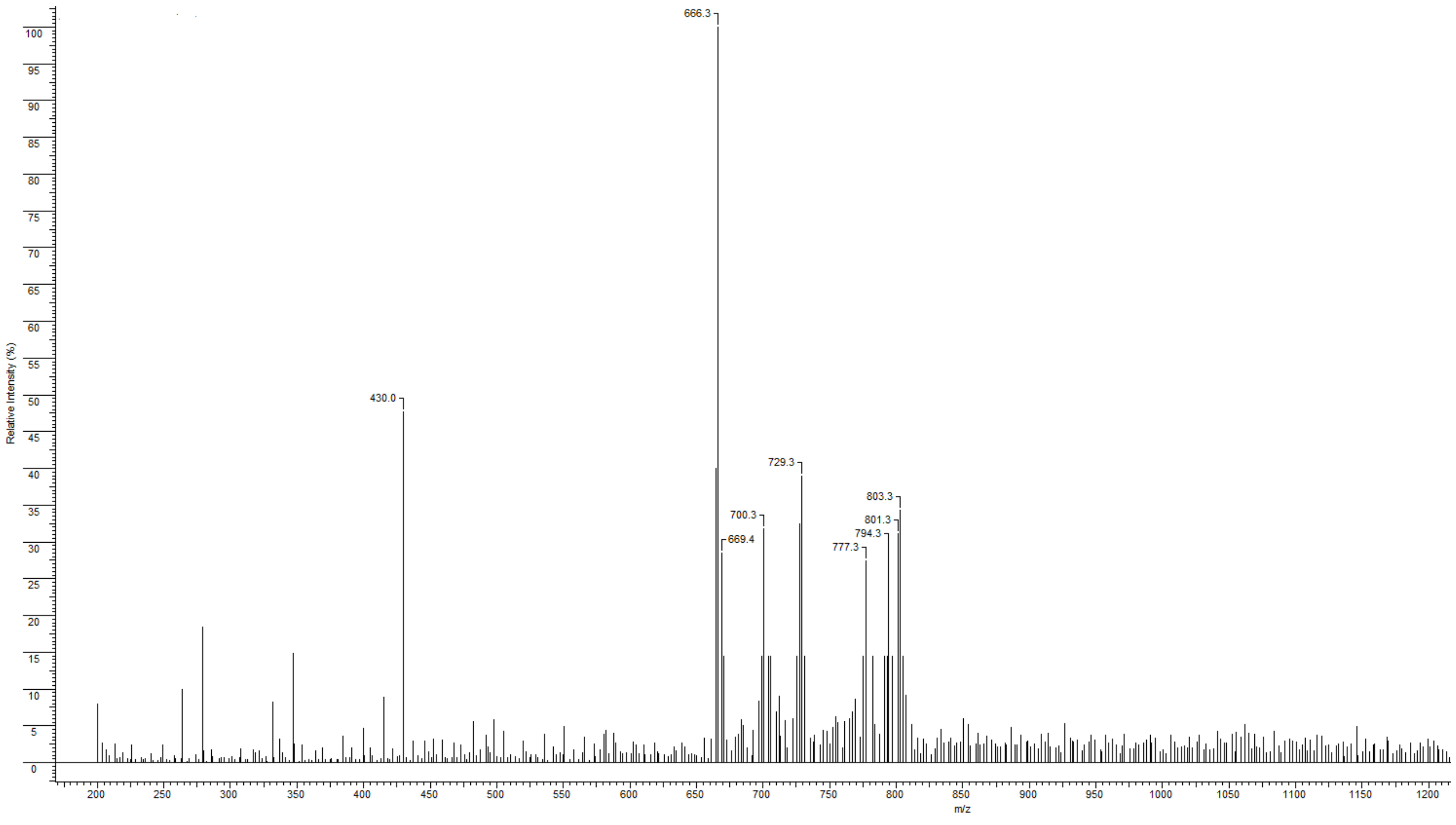

Figure S53: mass-spectrum (ESI) of compound **11**.

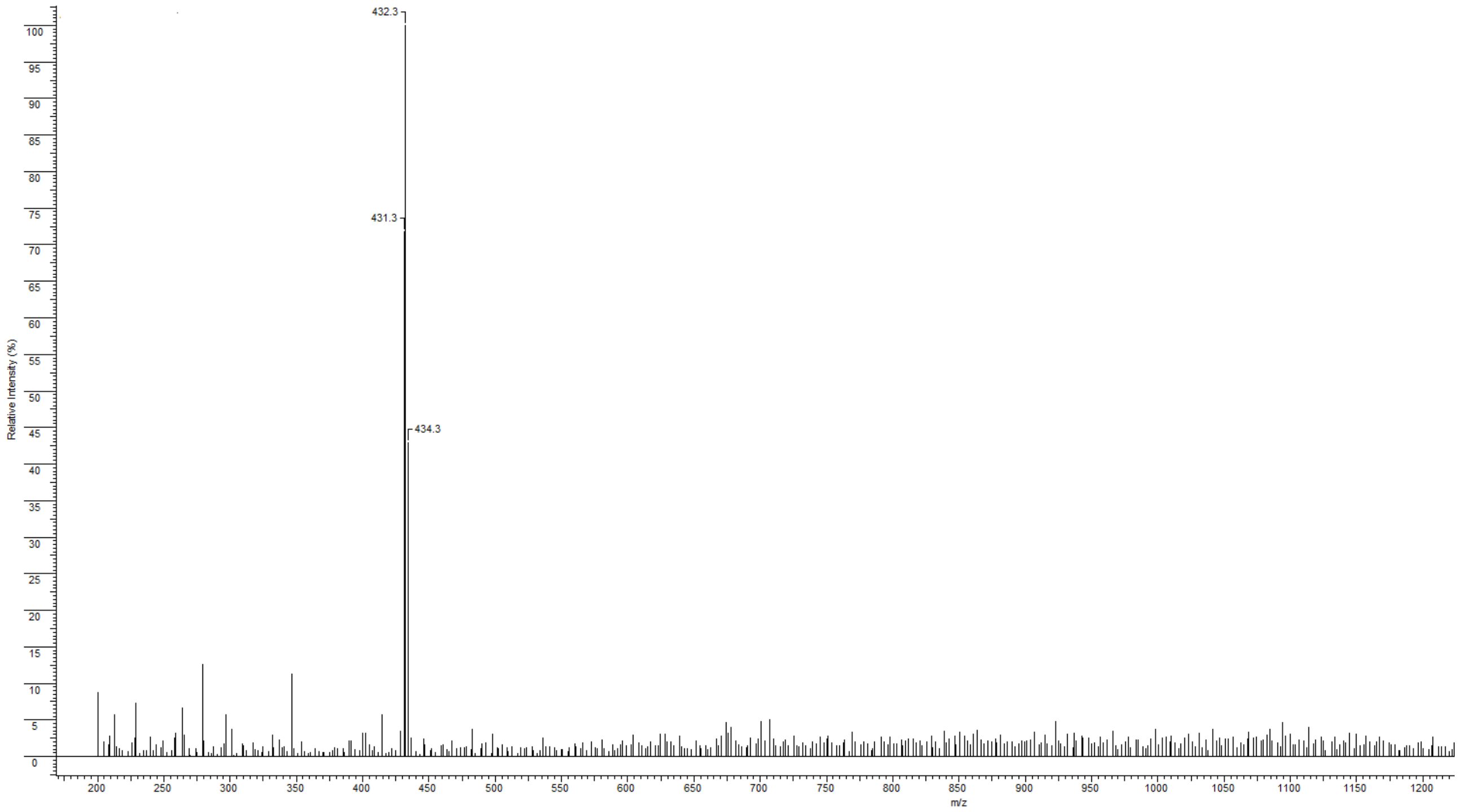

Figure S54: mass-spectrum (ESI) of compound **12**.

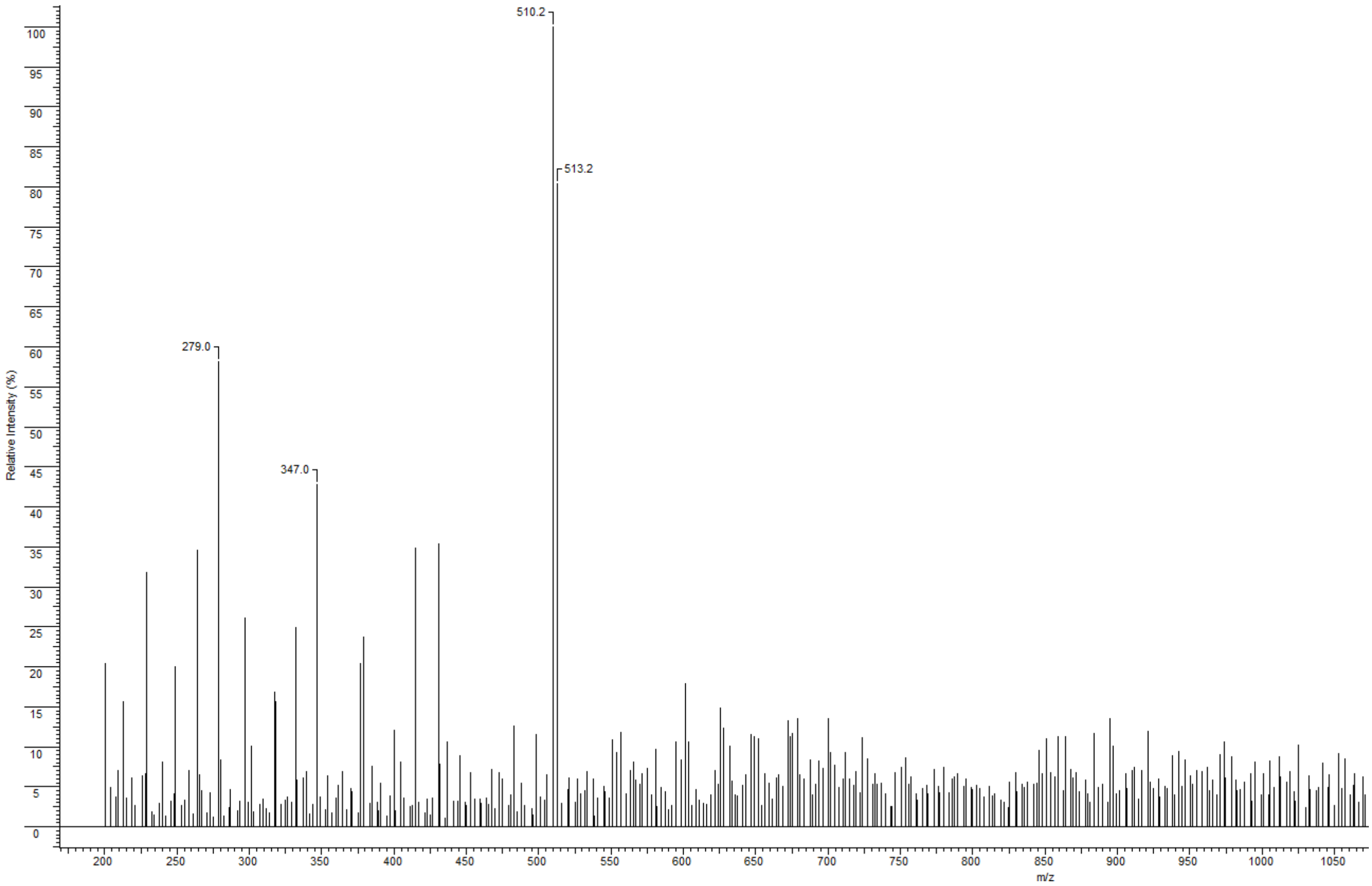

Figure S55: mass-spectrum (ESI) of compound **13**.

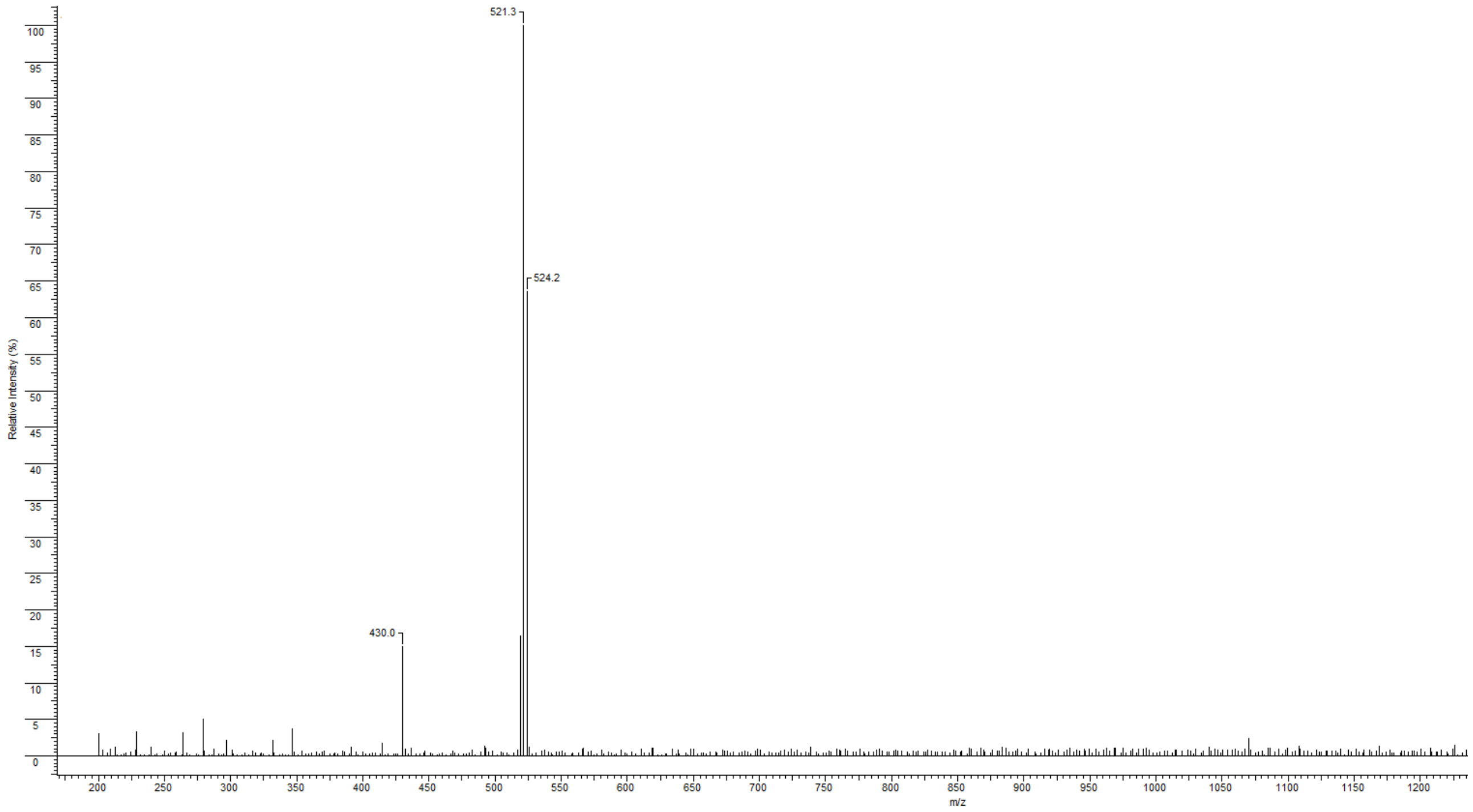

Figure S56: mass-spectrum (ESI) of compound **14**.

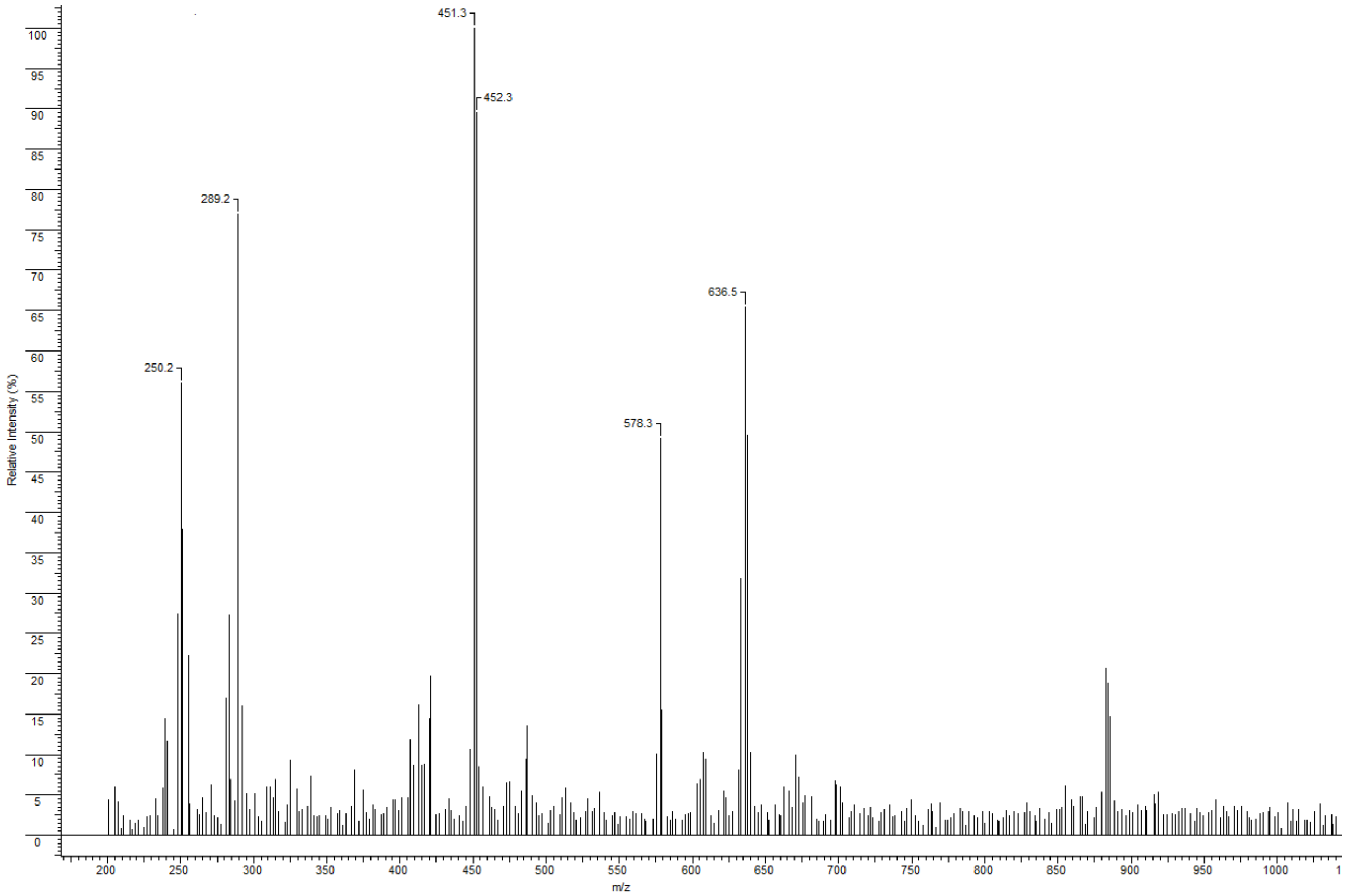

Figure S57: mass-spectrum (ESI) of compound **15**.

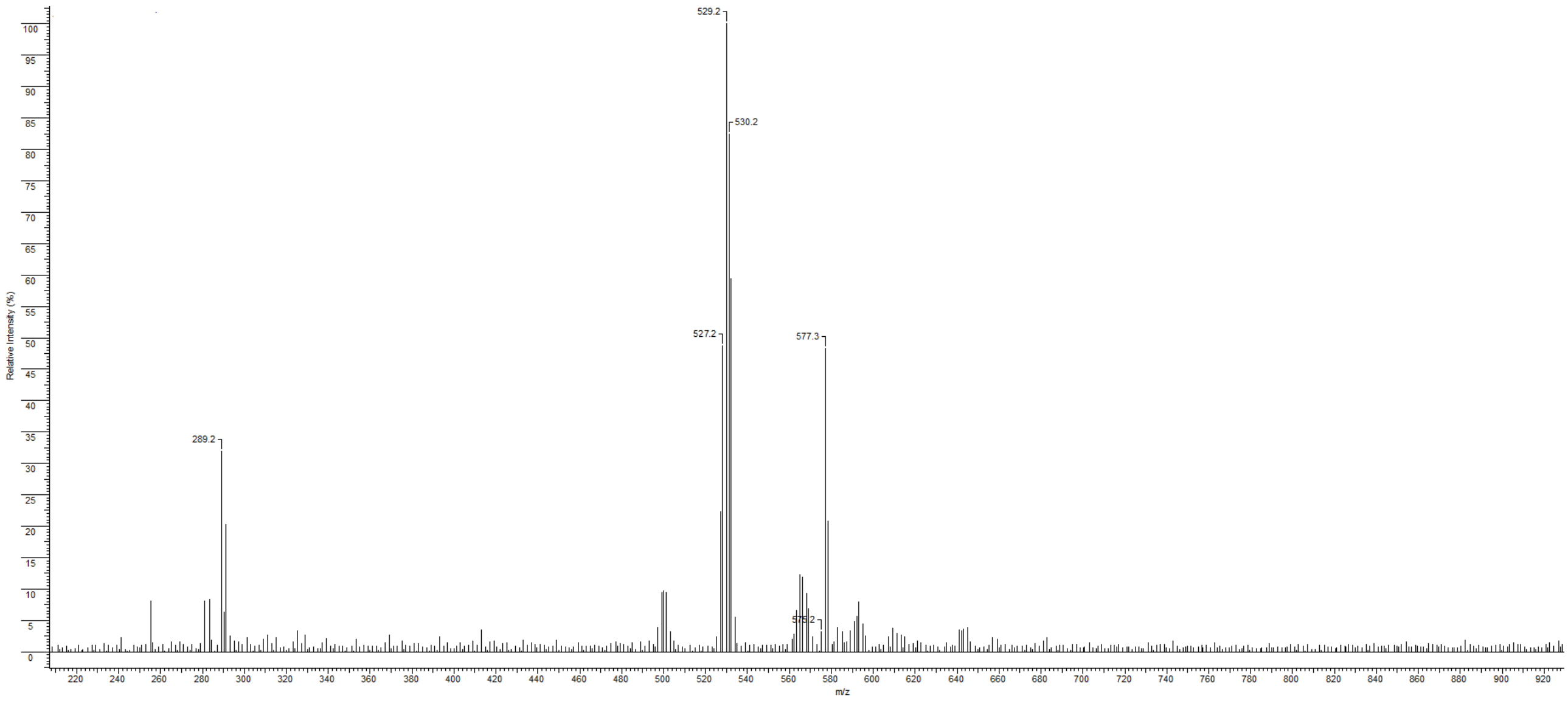

Figure S58: mass-spectrum (ESI) of compound **16**.

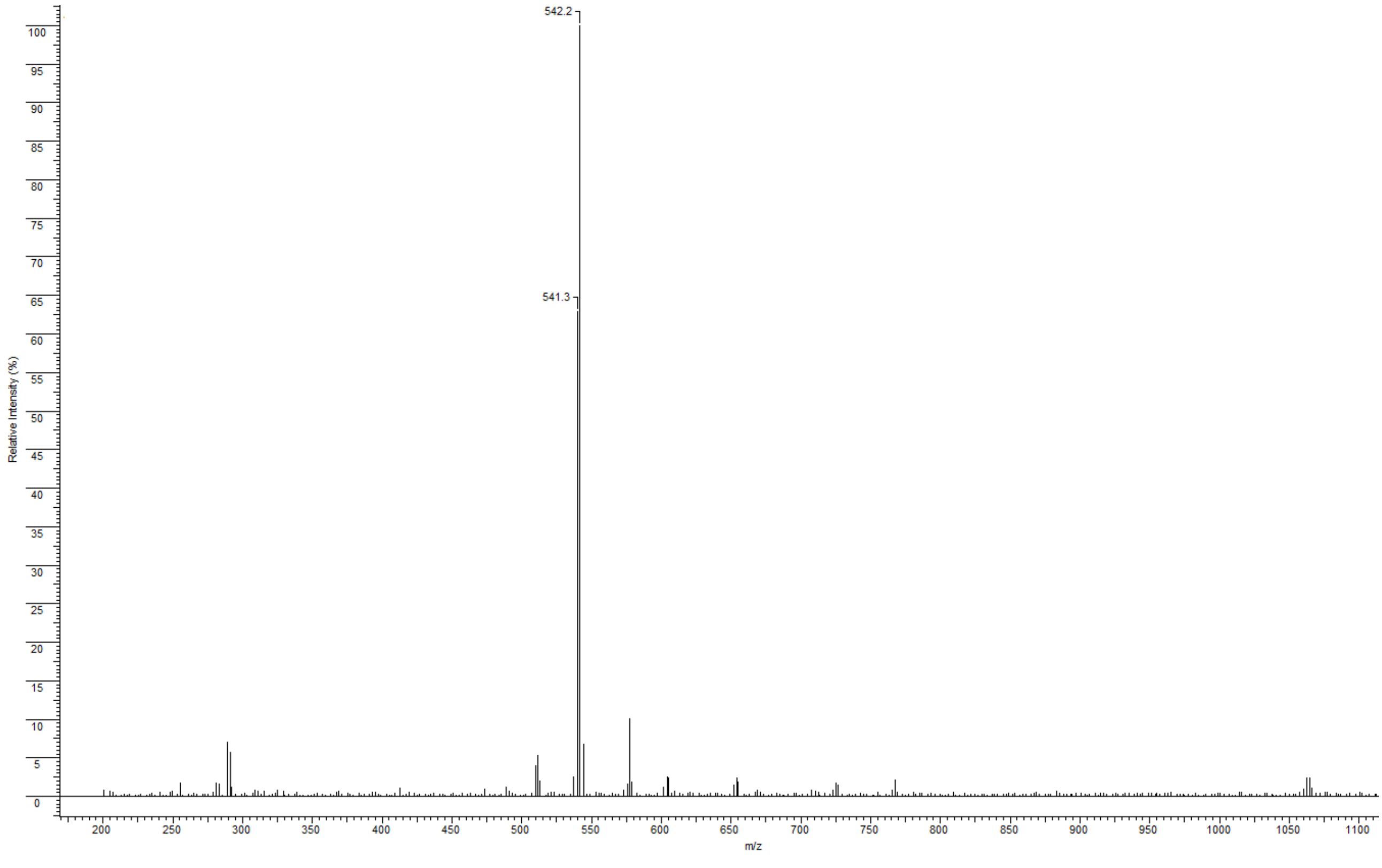

Figure S59– mass-spectrum (ESI) of compound **17**.

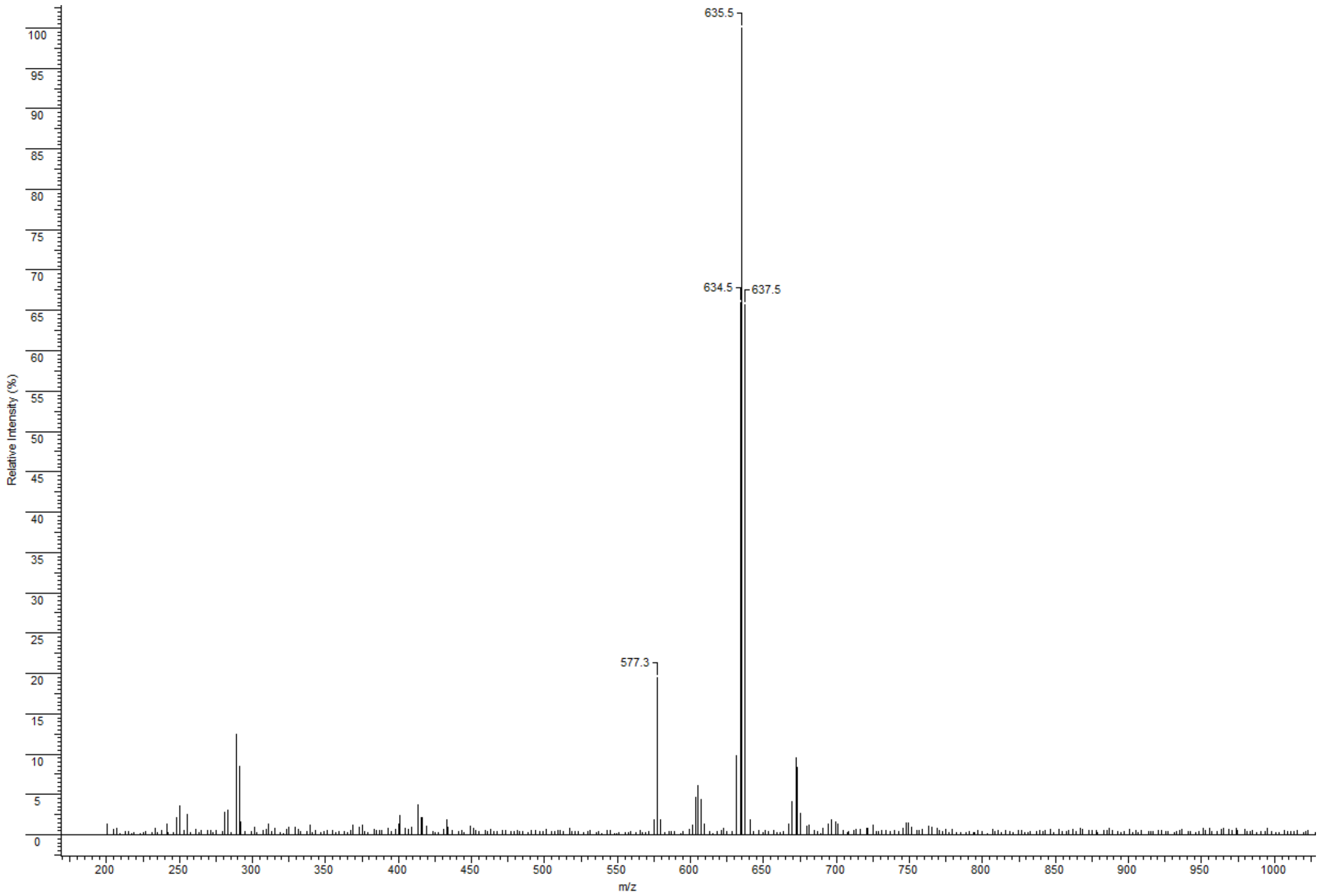

Figure S60: mass-spectrum (ESI) of compound **18**.

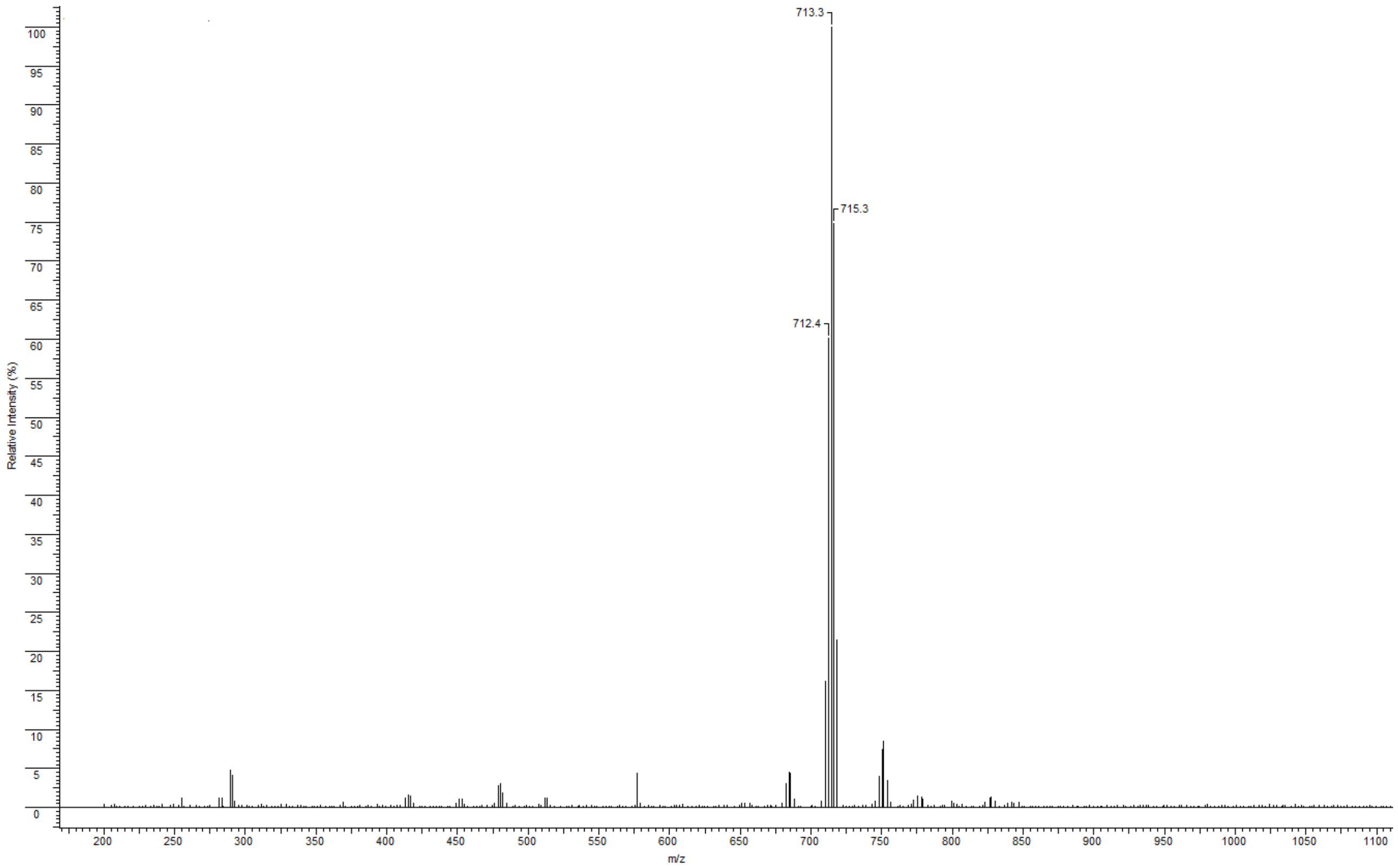

Figure S61: mass-spectrum (ESI) of compound **19**

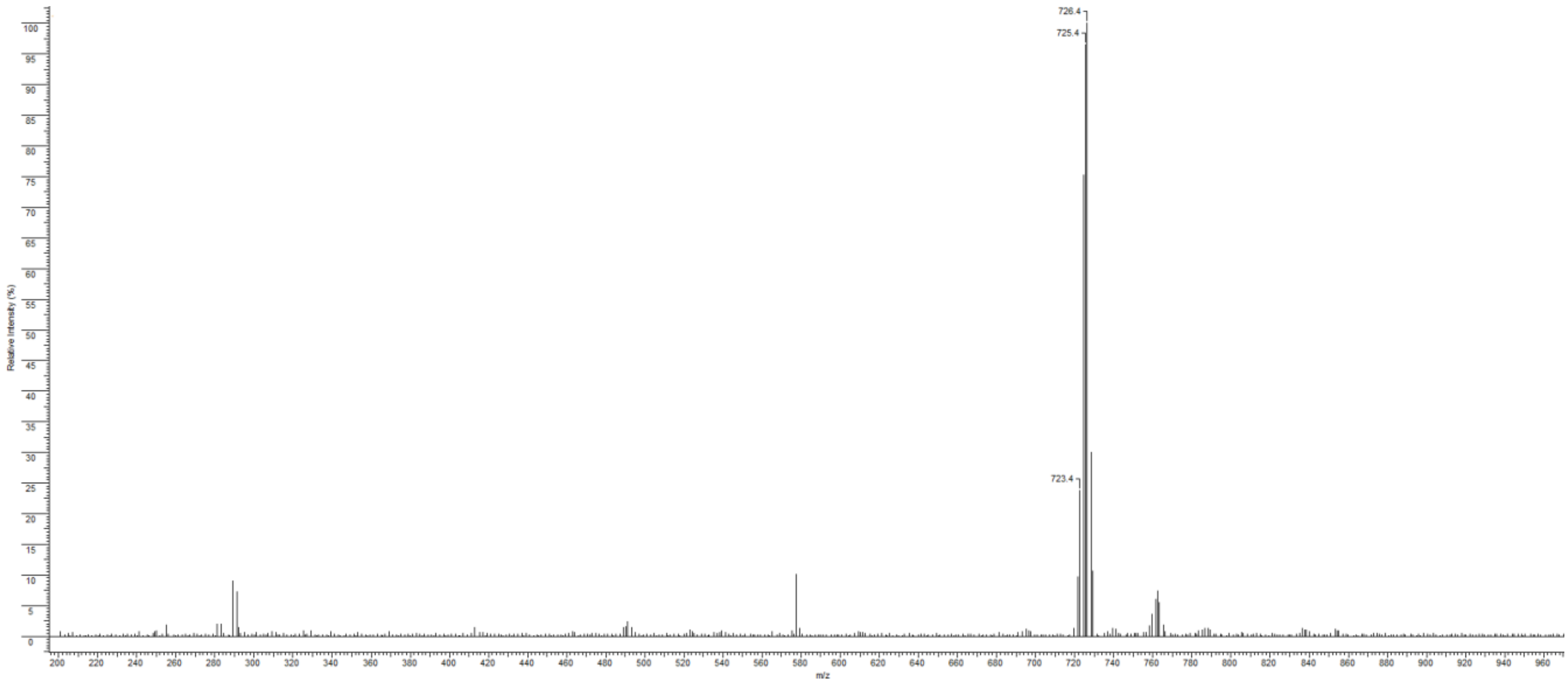

Figure S62: mass-spectrum (ESI) of compound **20**.

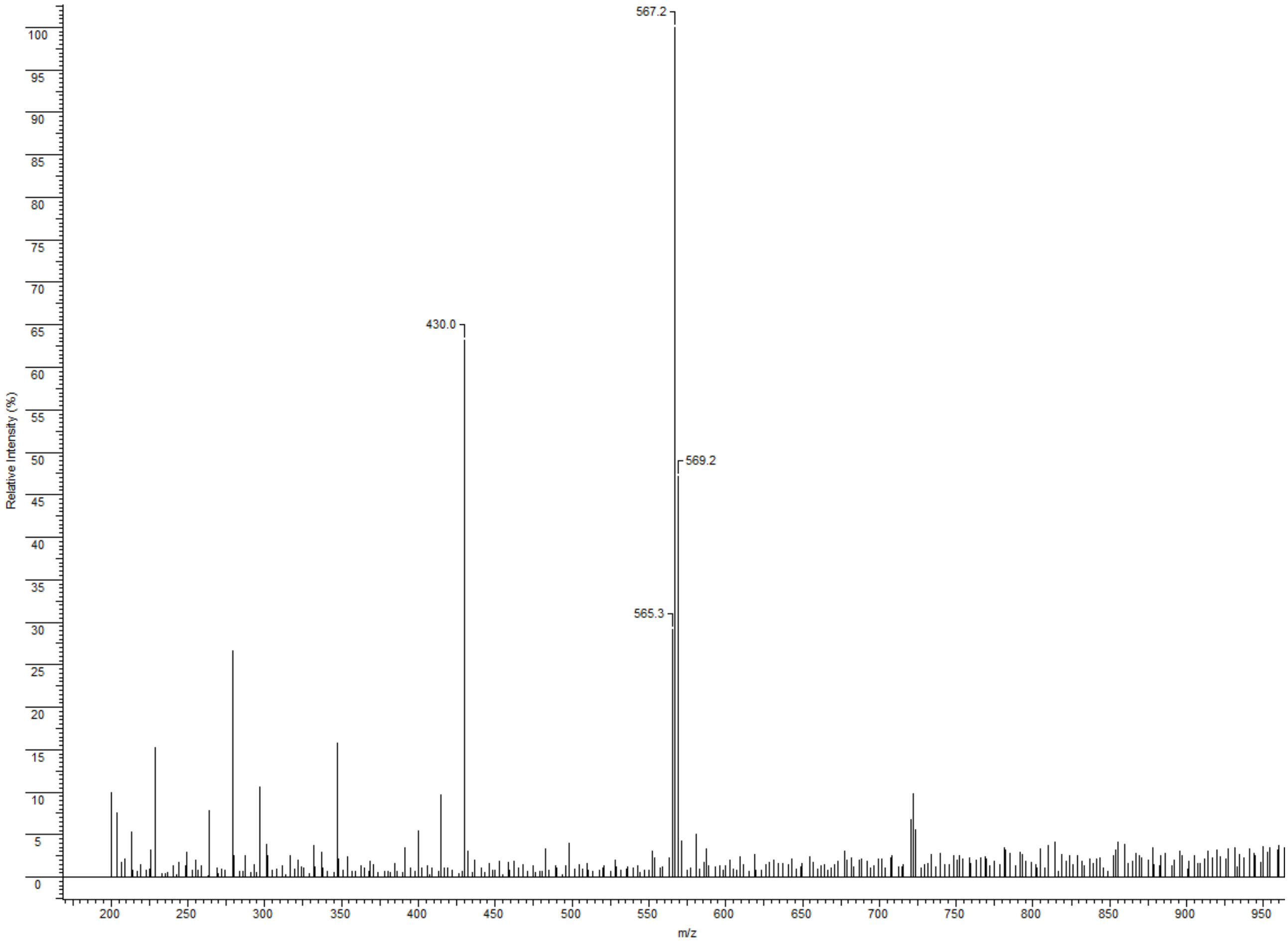

Figure S63: mass-spectrum (ESI) of compound **22**.

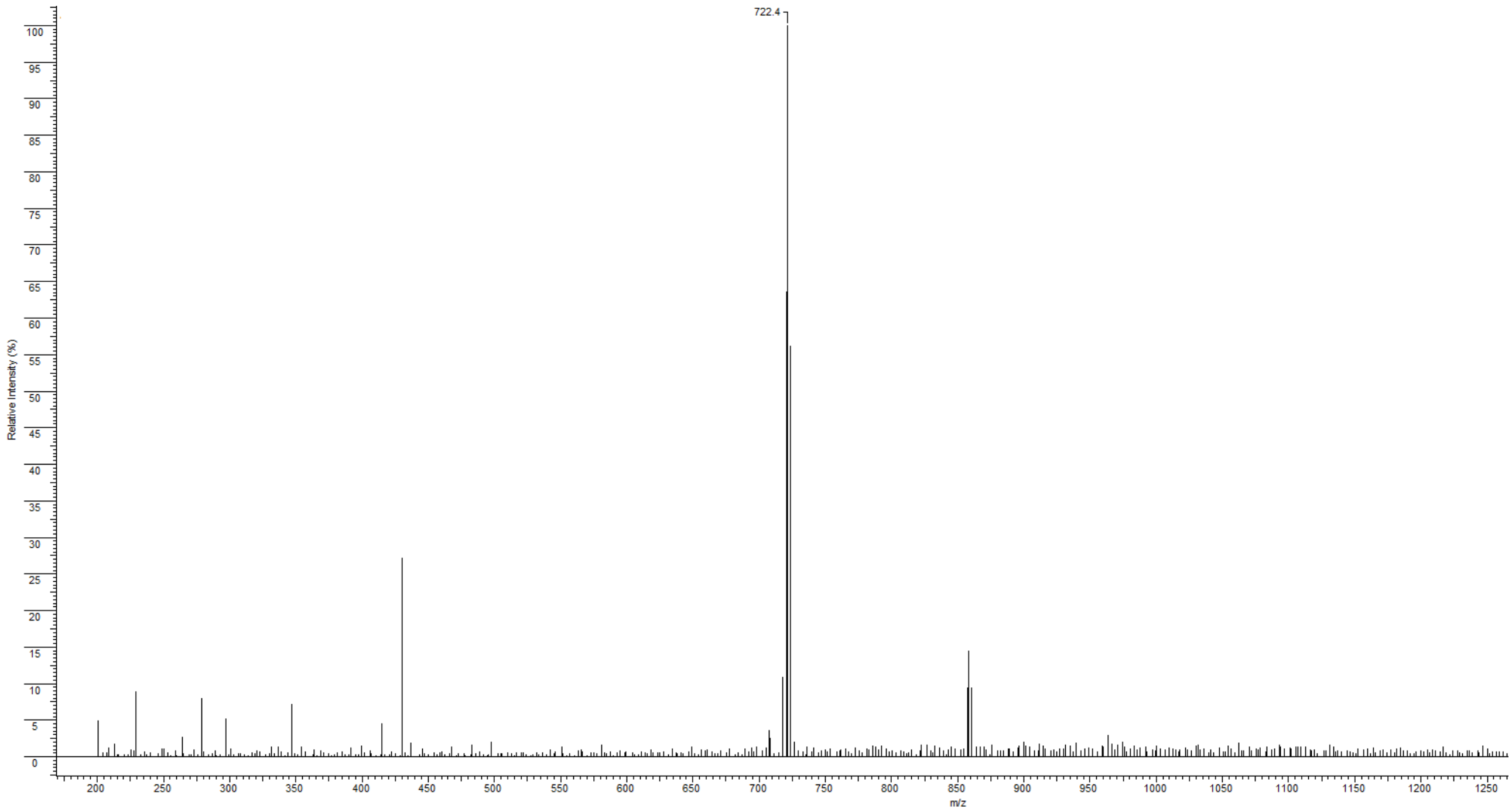

Figure S64: mass-spectrum (ESI) of compound **23**.

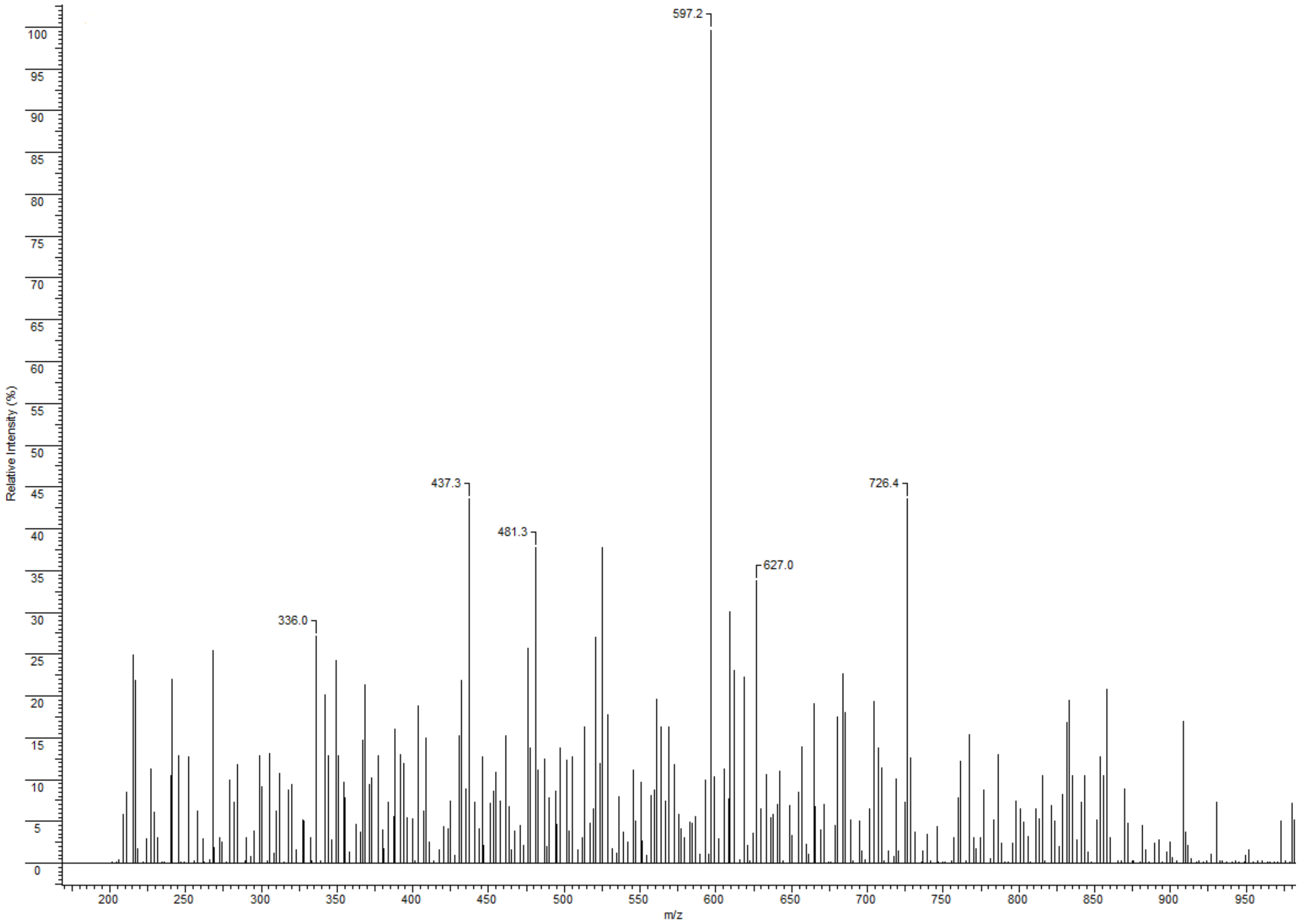

Figure S65: mass-spectrum (ESI) of compound **24**.

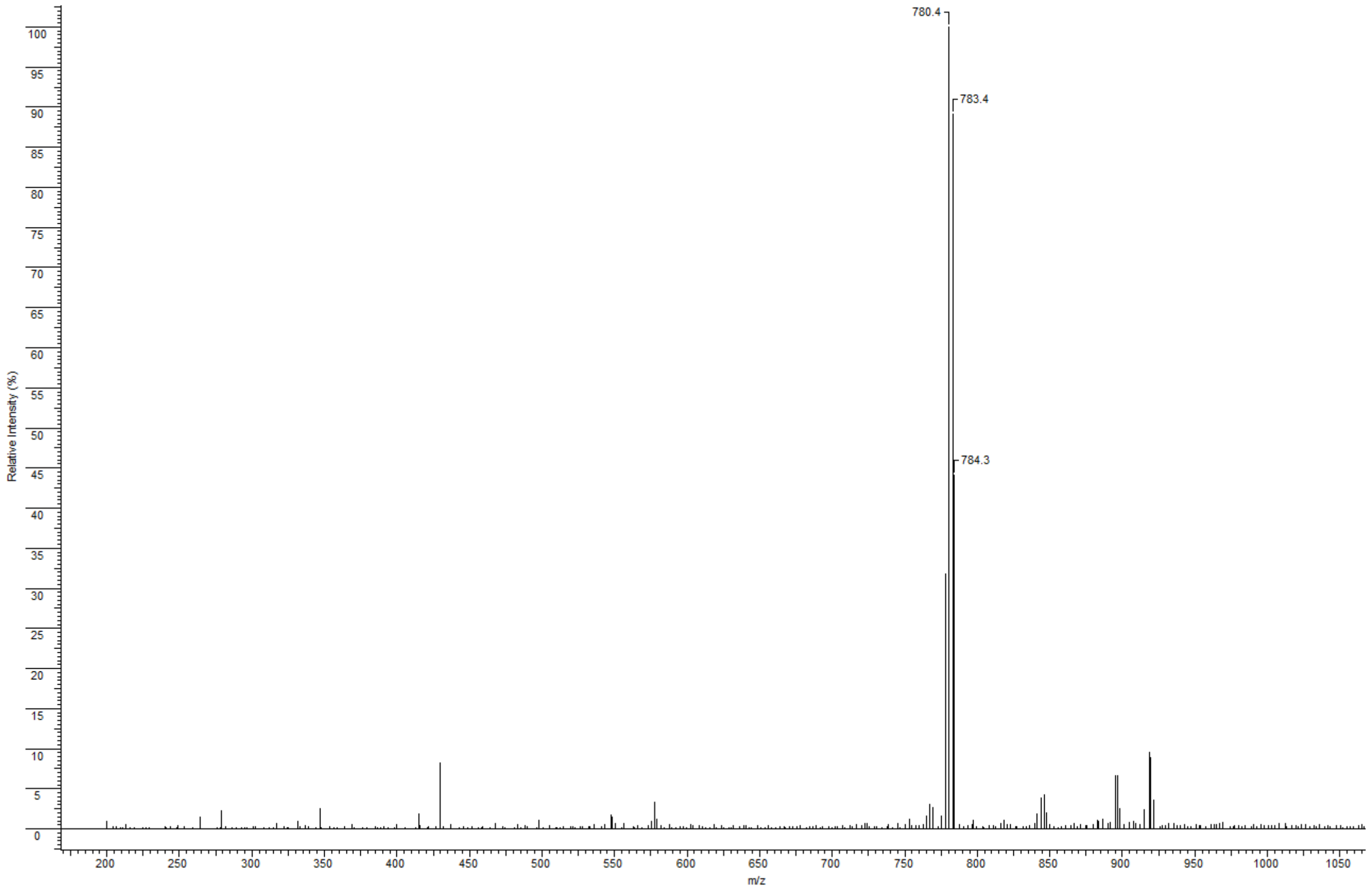

Figure S66: mass-spectrum (ESI) of compound **25.**

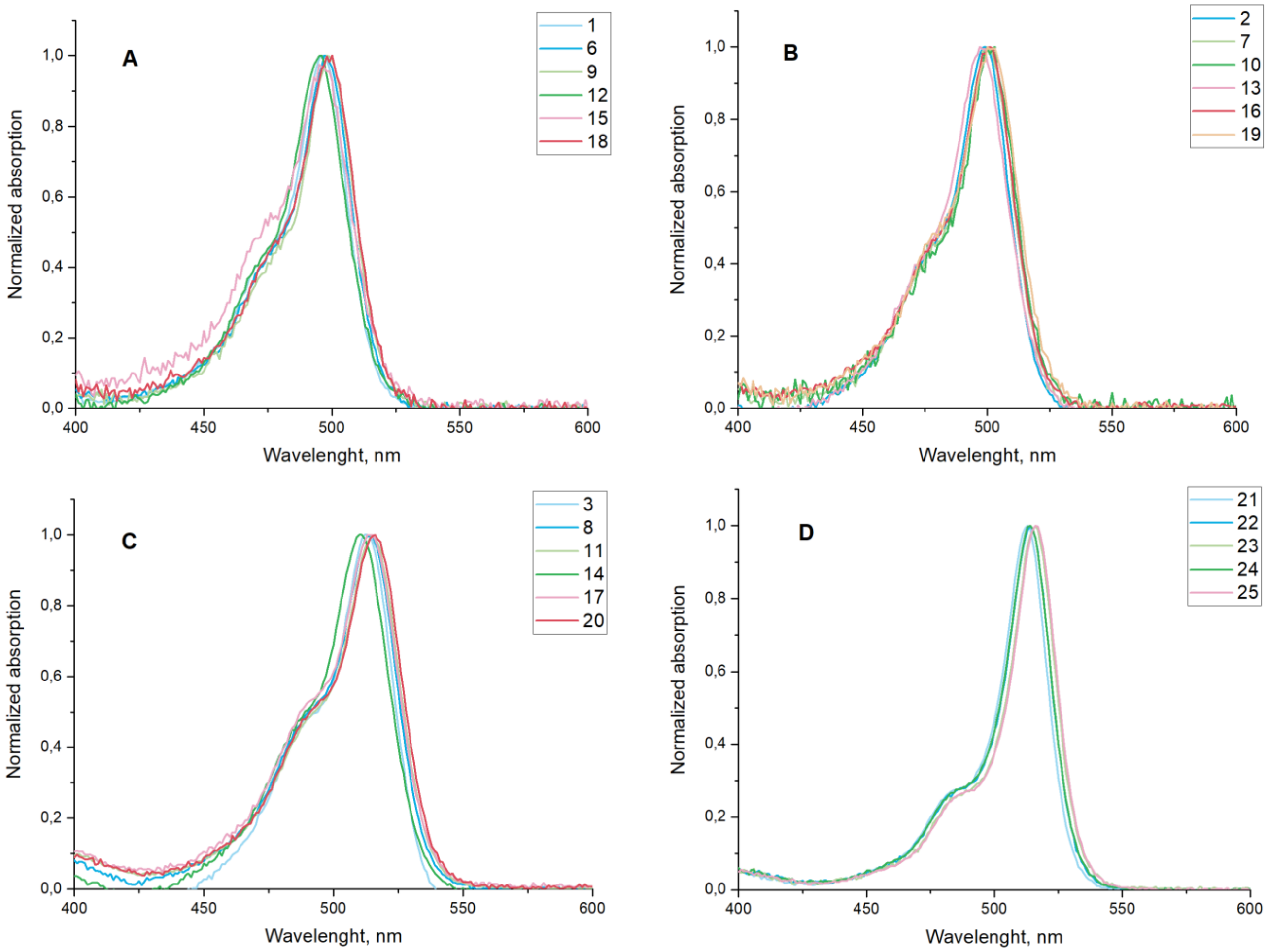

Figure S67: Normalized absorption spectra of compounds **1–3**, **6–25** in acetonitrile.
